# Emerging and reemerging infectious diseases: global trends and new strategies for their prevention and control

**DOI:** 10.1038/s41392-024-01917-x

**Published:** 2024-09-11

**Authors:** Shen Wang, Wujian Li, Zhenshan Wang, Wanying Yang, Entao Li, Xianzhu Xia, Feihu Yan, Sandra Chiu

**Affiliations:** 1 Key Laboratory of Jilin Province for Zoonosis Prevention and Control, Changchun Veterinary Research Institute, Chinese Academy of Agricultural Sciences, Changchun, 130000 China; 2 College of Veterinary Medicine, Jilin University, Changchun, Jilin China; 3 College of Veterinary Medicine, Jilin Agricultural University, Changchun, Jilin China; 4 Division of Life Sciences and Medicine, University of Science and Technology of China, Hefei, 230027 Anhui China; 5 Key Laboratory of Anhui Province for Emerging and Reemerging Infectious Diseases, Hefei, 230027 Anhui China; 6 Department of Laboratory Medicine, the First Affiliated Hospital of USTC, Division of Life Sciences and Medicine, University of Science and Technology of China, Hefei, Anhui China

**Keywords:** Infectious diseases, Biological models

## Abstract

To adequately prepare for potential hazards caused by emerging and reemerging infectious diseases, the WHO has issued a list of high-priority pathogens that are likely to cause future outbreaks and for which research and development (R&D) efforts are dedicated, known as paramount R&D blueprints. Within R&D efforts, the goal is to obtain effective prophylactic and therapeutic approaches, which depends on a comprehensive knowledge of the etiology, epidemiology, and pathogenesis of these diseases. In this process, the accessibility of animal models is a priority bottleneck because it plays a key role in bridging the gap between in-depth understanding and control efforts for infectious diseases. Here, we reviewed preclinical animal models for high priority disease in terms of their ability to simulate human infections, including both natural susceptibility models, artificially engineered models, and surrogate models. In addition, we have thoroughly reviewed the current landscape of vaccines, antibodies, and small molecule drugs, particularly hopeful candidates in the advanced stages of these infectious diseases. More importantly, focusing on global trends and novel technologies, several aspects of the prevention and control of infectious disease were discussed in detail, including but not limited to gaps in currently available animal models and medical responses, better immune correlates of protection established in animal models and humans, further understanding of disease mechanisms, and the role of artificial intelligence in guiding or supplementing the development of animal models, vaccines, and drugs. Overall, this review described pioneering approaches and sophisticated techniques involved in the study of the epidemiology, pathogenesis, prevention, and clinical theatment of WHO high-priority pathogens and proposed potential directions. Technological advances in these aspects would consolidate the line of defense, thus ensuring a timely response to WHO high priority pathogens.

## Introduction

**Fig. 1:**
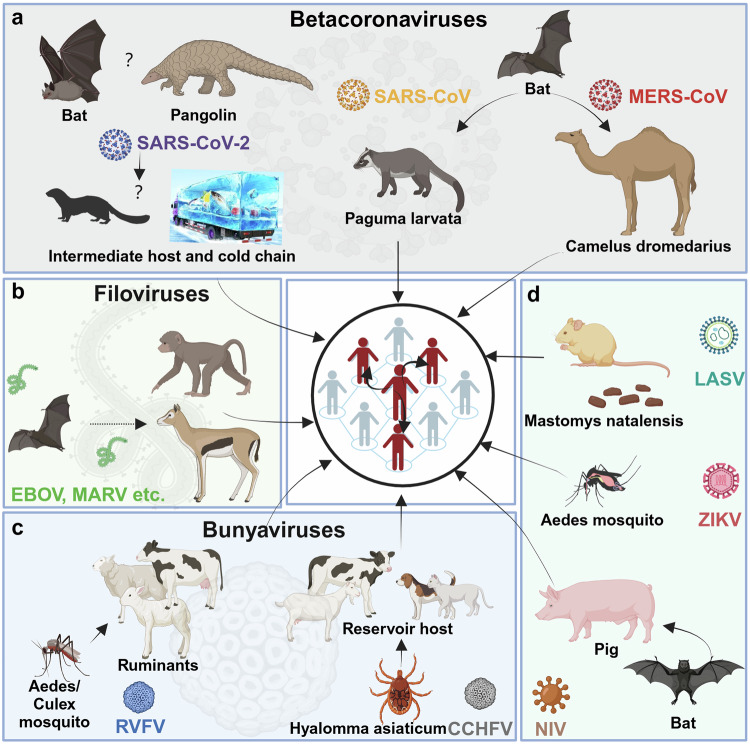
Transmission routes of high-priority pathogens to humans. **a** The source of severe acute respiratory syndrome coronavirus 2 (SARS-CoV-2) has not been identified. Bats and pangolins are presumed to be natural hosts, while transmission to humans may be mediated by intermediate hosts and cold chains.^935^ Severe acute respiratory syndrome coronavirus (SARS-CoV) and Middle East respiratory syndrome coronavirus (MERS-CoV) originate from bats and are transmitted to humans by Paguma larvata and Camelus dromedarius, respectively.^325^ **b** Filoviruses originate from bats and are transmitted to humans, such as nonhuman primates, by wildlife.^45^ **c** Rift Valley fever virus (RVFV) originates from Aedes mosquitoes and is transmitted to humans by ruminants.^936^ Crimean Congo hemorrhagic fever virus (CCHFV) originates from Hyaloma asiaticum and is transmitted to humans by ruminants and domestic animals.^551^ **d** Lassa fever virus (LASV) originates from Mastomys natalensis and is transmitted to humans by corresponding contaminants.^937^ Zika virus (ZIKV) is transmitted to humans via the bite of Ades mosquitoes.^938^ Nipah virus (NiV) originates from bats and is transmitted to humans via pigs.^939^ (Created in BioRender)

Over the past few decades, humanity has experienced novel and increasingly frequent waves of emerging and re-emerging infectious diseases, for which timely and effective countermeasures are lacking. Notably, the Ebola virus disease (EVD) outbreak in West Africa occurred between 2013 and 2016 and caused more than 11,000 deaths.^1^ In fact, many viral hemorrhagic fevers with high morbidity and mortality rates, including pathogens from Filoviridae, Arenaviridae and Bunyaviridae, which are associated with Marburg virus disease (MVD), Lassa fever (LF), Crimean-Congo hemorrhagic fever (CCHF) and Rift Valley fever (RVF), should be noted.^2–6^ Moreover, emerging beta-coronaviruses (Beta-CoVs) constitute a large group of highly transmissible respiratory pathogens associated with waves of outbreak. Severe acute respiratory coronavirus (SARS-CoV) emerged in 2003, Middle East respiratory syndrome coronavirus (MERS-CoV) emerged in 2012, and severe acute respiratory coronavirus 2 (SARS-CoV-2) emerged in 2019.^7–9^ More recently, SARS-CoV-2 infection has led to billions of cases and millions of deaths by 2024. In addition, Nipah virus (NiV), together with Zika virus (ZIKV), are causative agents of lethal encephalitis, which results in a cluster of associated neurological disorders, of which Zika virus disease is characterized by neonatal malformation (Table 1).^10,11^ In response, the WHO has launched a blueprint list of priority diseases to accelerate research and development (R&D) efforts for pathogens with the potential to cause future public health emergencies, depending on whether and how the pathogen is transmitted to humans, the extent of medical countermeasures available, and the severity and fatality rate of the corresponding disease.^12,13^ The most recent blueprint issue by WHO in February 2018, presented the most priority diseases, including EVD, LF, MVD, CCHF, Middle East respiratory syndrome (MERS) and severe acute respiratory syndrome (SARS), Nipah and henipaviral diseases, RVF, Zika, and “Disease X”, a yet unknown disease (Fig. 1) (Table 1).^14^

**Table 1:** WHO high-priority pathogens and corresponding diseases

Virus family	Virus	Receptor	Fatality rate	Clinical symptoms	Reference
Filoviruses	Ebola virus	NPC1	25–90%	Fever, fatigue, vomiting, sore throat, diarrhea, internal/external bleeding, conjunctivitis, uveitis, hypovolemic shock, multiorgan failure	^188^
	Marburg virus	NPC1	24–88%	Fever, headache, malaise, diarrhea, vomiting, lethargy, orchitis, conjunctivitis, iritis, retinitis, uveitis, increased intraocular pressure, multiorgan failure	^2^
Arenavirus	Lassa virus	Matriglycan	1–3%	Fever, sore throat, vomiting, malaise, hemorrhagic, neurological complications like hearing loss, conjunctivitis, cataract, retinal fibrosis, uveitis, iritis	^3,4^
Coronaviruses	SARS-CoV	hACE2	~10%	Fever, malaise, respiratory symptoms, pneumonia, headache	^7–9^
	MERS-CoV	hDPP4	30–40%	Asymptomatic to severe pneumonia, fever, cough, hemoptysis, diarrhea, vomiting, renal failure	^7,9^
	SARS-CoV-2	hACE2	0.35–3.85%	Pneumonia, fever, cough, fatigue, headache, shortness of breath, sore throat, multiple complications like loss of taste or smell	^7,891^
Bunyaviruses	CCHFV	LDLR	~30%	Headache, chills, photophobia, myalgia, hemorrhage, fever, multiple complications, backpain, joint pain, vomiting, severe bruising, uncontrolled bleeding	^5,560^
	RVFV	–	~1%	Hemorrhagic fever, malaise, meningitis, retinopathy, backpain, and dizziness, neurological complications like seizures, uveitis, retinitis, vasculitis	^6^
Paramyxovirus	Nipah virus	EphrinB2	40–75%	Encephalitis, fever, headache, vomiting, respiratory and neurologic, complications like seizures, pupillary abnormalities, oculomotor palsies, nystagmus	^10^
Flavivirus	Zika virus	–	~0.1%	Asymptomatic in most cases, mild symptoms including fever, rash headache, joint pain seen in some cases, microcephaly in fetus	^11^

*SARS-CoV* severe acute respiratory syndrome coronavirus, *MERS-CoV* Middle East respiratory syndrome coronavirus, *SARS-CoV-2* severe acute respiratory syndrome coronavirus 2, *CCHFV* Crimean Congo hemorrhagic fever virus, *RVFV* Rift valley fever virus, *NPC1* Niemann-PickC1, *LAMP1* lysosome-associated membrane protein 1, *hACE2* human angiotensin converting enzyme 2, *hDPP4v* human dipeptidylpeptidase-4, *LDLR* low density lipoprotein receptor, – not applicable

In R&D efforts, appropriate animal models are stepping stones that help provide preliminary insights into the epidemiology and pathogenesis of these high-priority diseases and support preclinical evaluation of preventive and therapeutic approaches. Common small laboratory animals, such as mice, hamsters and guinea pigs, are generally less susceptible to these WHO high-priority pathogens.^15–18^ Novel techniques, such as immunodeficiency, receptor-transgenic/transduction, virus adaptation and humanization, have been actively used to establish competent infection models. Both traditional and emerging vaccine platforms, including inactivated vaccines, live attenuated vaccines, protein subunit vaccines, viral vector vaccines and nucleic acid vaccines, are actively being developed. Owing to the interdisciplinary collaboration between immunology, structural biology, multiomics, and artificial intelligence (AI), antibodies and small molecule drugs, which exhibit improved targeting, tolerance, and stability, are being developed as popular therapies for infectious diseases.

In this review, we summarize the overall profile of the epidemiology, pathogenesis, and rational application of animal models, vaccines, antibodies, and small molecular drugs for the aforementioned priority diseases. Moreover, we prospect directions and novel techniques that may supplement, replace, or guide prevention and control strategies, such as the use of organoids and AI. Overall, comprehensive knowledge of global trends and cutting-edge technologies could accelerate breakthroughs in the prevention and control of WHO blueprint priority pathogens.

## Filovirus diseases

### Etiology, epidemiology, and pathogenesis of filovirus diseases

The Filoviridae family includes a large group of filoviruses that cause lethal viral hemorrhagic fever (VHF). With a genome size of approximately 19 kb, filoviruses are nonsegmented single-stranded negative-sense RNA viruses. The eight subgenomic mRNAs encode seven structural proteins, including nucleoprotein (NP), glycoprotein (GP), matrix protein VP40, viral proteins VP24, VP30, and VP35, and polymerase L.^19^ The Ebola virus genus comprises 6 different species: Zaire virus (EBOV), Reston virus (RESTV), Sudan virus (SUDV), Bundibugyo virus (BDBV), Tai Forest virus (TAFV), and Bombali virus (BOMV).^20^ The amino acid sequences of SUDV and BDBV GP vary from that of EBOV by 50 and 30%, respectively.^21^ GP is the only structural protein assembled on the surface of the virion and is responsible for viral attachment, entry, membrane fusion and release. Additionally, GPs are main targets for triggering the immune response.^22^ GP harbor two disulfide-linked furin cleavage sites and can be cleaved into GP1 and GP2.^23^ The GP1 subunit of GP is composed of the receptor binding domain (RBD), two highly glycosylated domains, a glycan cap that shields the receptor binding site (RBS), and a mucin-like domain (MLD) with many N- and O-linked glycosylation sites. Axl, TIM-1, DC-SIGN, integrins, and C-type lectins are attachment factors.^24–26^ Filovirus infection of initial innate immune cells, such as monocytes, macrophages, dendritic cells, hepatocytes, and endothelial cells, is dependent on the expression of C-type lectins, while infection of other cell types later in infection appears to depend on other attachment factors.^27,28^ It is known that membrane-anchored C-type lectins and EBOV GPs interact mostly through the MLD.^25,29,30^ After GPs bind to attachment factors, the virus particles are absorbed into endosomes via micropinocytosis.^31–33^ GP removes the glycan cap and MLD, exposing the RBD, following protease processing in late endosomes and/or lysosomes at low pH. This results in the formation of GPCL, which can bind the intracellular receptor Niemann-Pick C1 (NPC1).^34,35^ The GP2 subunit consists of a stalk domain, a transmembrane (TM) domain that anchors GPs to the viral membrane, and an internal fusion loop (IFL) that contributes to the viral fusion with target cell membranes. Notably, the antagonism of GP2 with tetherin proteins during membrane fusion and the spatial shielding effect of GP exist in filoviruses.^36,37^ GP shields the transmembrane protein Fas and interferes with its spatial shielding effect, which helps to protect MARV-infected cells from premature death.^37^ The shielding effect of GPs varies among strains, and the spatial shielding of host proteins by GPs of MARV Angola is more pronounced than that of Musoke GPs, suggesting that GPs may play a role in the specific pathogenicity of the virus.^38^ VP35 was reported to block the phosphorylation of endogenous STAT1 and suppress the nuclear translocation of STAT1 to facilitate viral replication.^39^ Further evidence has demonstrated that the structural protein VP40 effectively assists in thwarting the host immune response to IFN.^40^ The main product of the GP gene, known as the secreted glycoprotein, sGP, is a 364-amino acid protein that contains 295 amino acids from the N-terminus of GP but lacks the MLD and GP2.^41^ Significant linear and conformational amino acid epitopes that are accessible for antibody binding are shared by sGP and GP. sGP can antagonize the host immune response by producing nonneutralizing antibodies.^42^ Furthermore, interactions between sGP and neutrophils may disrupt the physical interactions that FcgR IIIB and complement receptor 3 share, preventing neutrophil function.^43^ By promoting the restoration of endothelial barrier function, sGP is also believed to intensify anti-inflammatory effects.^44^

Filoviruses originate from bats and are transmitted to humans by wildlife, such as nonhuman primates.^45^ Filoviruses spread through broken skin or mucous membranes in the eyes, nose, or mouth in contact with contaminated blood or body fluids, objects, and infected fruit bats or NHPs. Domestic pigs in China and the Philippines are hosts of RESTV.^46,47^ Identification of the natural host becomes considerably more challenging due to the striking similarity between human-to-human transmission of MARV and that of EBOV and episodic outbreaks. Apart from the primary natural host of MARV, the North African fruit bat (Rousettus aegyptiacus) and other pteropod bats such as the South African hoofed bat (Hipposideros caffer) have also been demonstrated to be natural reservoir hosts.^48,49^ Different species of the Ebola virus genus exhibit distinct epidemiological characteristics. Over 34,000 human cases and 15,000 deaths have been reported for Ebolavirus infections. SUDV resulted in 779 cases and 412 fatalities.^50^ Only one documented human case of TAFV occurred in Côte d’Ivoire in 1994. In contrast, RESTV did not cause illness in human infections. The largest Ebola outbreak to date, which was associated with EBOV-Makona, resulted in more than 28,600 cases and 11300 fatalities between 2014 to 2016 in Beria, Guinea, and Sierra Leone. More recently, SUDV caused 164 cases and 77 fatalities in Uganda from 2022 to 2023. In 1967, MARV was first identified in Germany. Since then, MVD outbreaks have occurred more than 15 times and causing 474 cases and 379 deaths.^51^ In recent years, imported cases of EVD have been reported in Europe, Asia, and the Americas, which emphasizes the urgent need to develop medicinal countermeasures.

EVD can lead to a wide range of diseases, ranging from asymptomatic infection to severe disease in humans and other primates, depending on the virus species, which might be due partly to genetically determined differences in innate immune responses to the viruses.^52^ It is characterized by the rapid onset of symptoms such as fever, fatigue, muscle pain, headache, sore throat, vomiting, diarrhea, rash, and multiorgan failure, as well as hemorrhagic manifestations in patients in the terminal stages.^1^ The incubation period of EVD, the time from viral infection to symptom onset, is 2–21 days. Additionally, decreased white blood cell (WBC) counts, platelet counts, and elevated liver enzymes are found in laboratory tests. Similarly, MVD progresses rapidly with fever, headache, malaise, muscle aches and pain. Watery diarrhea, abdominal pain, cramping, nausea, and vomiting occur during the symptomatic period. In some cases, diarrhea can persist for one week. Many patients experience severe hemorrhage within 7 days post infection (dpi), which is closely associated with fatal outcomes. Death usually occurs 8–9 days after the patient becomes symptomatic. MVD is a virulent zoonotic illness with an overall case fatality rate of approximately 50%.^53^

Severe filovirus disease can be attributed to a complex of pathogenetic mechanisms that allow the virus to invade and suppress innate and adaptive immune responses, infect and kill a wide variety of cell types, and elicit strong inflammatory responses and diffuse intravascular coagulation, producing a syndrome resembling septic shock. Specifically, after filovirus entry, the primary target of filovirus is antigen presenting cells (APCs), including dendritic cells, macrophages, and monocytes, which cause cell degeneration and necrosis,^52,54^ followed by rapid viral replication, resulting in clotting, blockage of blood vessels, and retention of tissue fluid, blood macromolecules, and cells. When the virus invades endothelial cells located in internal organs such as the heart, blood vessels, liver and kidney, it causes small pores in blood vessels and organs, and blood components flow through these pores. Therefore, the onset of filovirus hemorrhagic fever is mainly characterized by massive bleeding. An important organ for filovirus replication is the liver, where the virus preferentially targets lymphoid tissues.^55^ Simultaneously, a large amount of synthesized envelope glycoproteins leads to cell necrosis and induces immune suppression and damage, which are also the main reasons for the high mortality of filoviruses. In contrast, studies in nonhuman primates have shown that blocking certain host responses, such as the coagulation cascade, can result in reduced viral replication and improved host survival.^56^

### Animal models for filovirus diseases

Given that limited medical countermeasures are available for filovirus diseases, preclinical animal models, including mouse, hamster, guinea pig, ferret, and nonhuman primate models, have been actively developed and applied to accelerate breakthroughs in medical countermeasures. In this section, animal models for filovirus diseases are reviewed in detail (Table 2).

**Table 2:** Animal models of filoviruses

Species	Approaches/animals	Pathogens	Dose	Route	Lethality	Signs of Disease	Strengths/Weaknesses	References
Mouse	Neonatal mice	EBOV	1 PFU	IP	100%	No	No need for additional sensitive approaches/Not applicable for evaluation of immune correlates	^59^
	Mice adapted virus	EBOV Mayinga	10^2^ PFU	IP or IC	100%	Ruffled fur, malaise, weight loss, and hemorrhage	Target cells and tissue tropism comparable to humans/Only sensitive to IP injection, additional mutants introduced by adaptation	^799^
				SC	0–100%			
	IFN-α/βR^−/−^ mice	maEBOV	10^3^ PFU	IP or SC	100%	Lethargy, weight loss, anorexic and piloerection	Lethality to wild type viruses, valuable for the pathogenesis study/Mimicking limited clinical features of infection, barrier conditions needed, not applicable to vaccine evaluation	^58,70^
		EBOV Mayinga	10^3^ PFU	IP				
		EBOV E718	10 TCID_50_	IP or Aerosol				
		SUDV Boneface	10^3^ PFU	IP				
		MARV Popp	10 TCID_50_	IP or Aerosol				
	STAT1−/−mice	maEBOV	10^3^ PFU	IP or SC	100%	Ruffled fur, malaise, weight loss	Lethality to wild type viruses, valuable for the pathogenesis study/Inconsistent clinical performance for different subtype of virus infections, barrier conditions needed, not applicable for vaccine evaluation	^58,69,71^
		EBOV Mayinga	10^3^ PFU	IP	0			
		EBOV Kikwit	10^2^ PFU	IP	40%			
		SUDV Gulu			100%			
		BDBV			20%			
		MARV Angola			80%			
	SCID mice	maEBOV/EBOV/SUDV	10^3^ PFU	IP or SC	100%	Weight loss	Natural susceptibility to specific isolates/Inconsistent susceptibility to different subtypes or isolates	^58^
		gpaMARV Ravn						
		MARV Musoke	10^3^ PFU	IP	100%	Ruffled fur, weight loss, anorexia, hunched posture	Shorter time to death than other SCID mice model/Lack typical biochemical parameters	^72,73^
		MARV Ravn						
		MARV Ci67						
		maMARV Angola	2.72 TCID_50_	IP	100%	Weight loss		
	SCID mice	maMARV-Ravn	10^3^ PFU	IP	100%	Weight loss, ruffled fur, hunched posture, lethargy	Exhibited most clinical hallmarks in humans/Time consuming, additional mutants introduced by virus adaptation	^57,73^
		maMARV Angola	10^2^ TCID_50_					
	Humanized mice	EBOV Mayinga	10^5^ TCID_50_	IP	100%	Ruffled fur, weight loss, fever, and hunched posture, hypothermia, and moribundity	Recapitulating disease severity of different EBOV subtypes in humans/Lack of disease hallmarks	^76^
		EBOV Makona	10^3^ FFU	IN	92.86%			^77^
		SUDV Gulu			71.43%			
		BDBV Bundibugyo			28.58%			
		TAFV Pauleoula			20%			
		RESTV			19.2%			
		EBOV Makona		IM	56%			^79^
		MARV-Angola			25%	Weight loss, anemia	Reveal how MARV modulate specific components of the immune system/Poor disease severity	
Syrian hamster	Hamster	maEBOV	10^3^ FFU	IP	100%	Ruffled fur, malaise, severe coagulation disorders	Displaying most clinical hallmarks of EVD/Virus adaptation needed	^82^
	STAT2^−/−^hamster	MARV RAVV	10^5^ PFU	IP	0	Ruffled fur, lethargy, hunched posture, and weight loss	Susceptibility to wild-type viruses/barrier conditions needed, not applicable for vaccine evaluation	^84^
		MARV Musoke			100%			
		MARV Voege			100%			
		MARV Angola			80%			
	Hamster	haMARV	10^3^ PFU	IP	100%	Weight loss, fever, rash, coagulation abnormalities, and hemorrhagic	Recapitulating most clinical hallmarks in humans/Virus adaptation needed	^85^
Guinea pig	Guinea pig	gpaEBOV Mayinga	10^4^ PFU	SC/IP	100%	Fever, weight loss, diarrhea, anorexia, ataxia, hemorrhage, ceased eating, dehydration	Similar disease features to humans/Additional mutants induced by virus adaptation	^87,89^
		gpaSUDV	10^3^ TCID_50_	IP	100%	Weight loss, coagulation disorders		^91^
		gpaMARV Angola/Ravn	5000 PFU	IP	100%	Weight loss, fever, and hypothermia	Differentiated pathogenicity among MARV strains/Additional mutants induced by virus adaptation	^93^
Ferret	–	EBOV Kikwit	10^3^ PFU	IN	100%	Hypothermia, rash, hemorrhage, weight loss, depression, diarrhea, dehydration, nasal and ocular discharge, dyspnea, hunched posture, and altered gait	Uniform lethality with wild type viruses, low dose needed, susceptible to multiple challenge routes/Inconsistent clinical performance for different subtype of virus infections compared with humans, lack of apparent disease following ocular challenge, lack of disease hallmarks	^96^
		SUDV Gulu						
		BDBV						
		rgEBOV-C07	1 PFU	IM				^97^
		EBOV Makona-C07	200 TCID_50_	IM or IN	100%	Rash, fever, weight loss, and coagulation disorders		^98^
		BDBV	159 TCID_50_	IM				
		EBOV	13.3 PFU	Oronasal	100%	Rash, fever, weight loss, lethargy, unkempt appearance, dyspnea, diarrhea, and coagulation disorders		^99^
			76.6 PFU	Oral				
			76.6 PFU	Ocular	0	No		
		SUDV Boneface	1260 TCID_50_	IM or IN	100%	Fever, weight loss, malaise, anorexia, dyspnea, absence of urine, diarrhea, and coagulation disorders		^100^
		RESTV		IM				^103^
				IN				
		gpaEBOV	10^4^ PFU	IP	100%	Weight loss, anorexia, fever, hemorrhages and rash		^892^
NHPs	Rhesus monkey	EBOV	10^3^ FFU	IM	67–100%	Fever, multiple organomegaly, pancytopenia, and coagulation disorders	Naturally, susceptible model/Mimicked limited physiological features of infection, expensive and ethical issues	^83,107–109,112^
		SUDV Gulu	10^3^ FFU	IM	91%	Fever, depression, rash, anorexia, dyspnea, dehydration, shock and multiorgan failure	Recapitulate the disease course seen in human, aerosolized exposure model/Expensive and ethical issues	^116^
		SUDV Boniface	500 PFU	Aerosol	100%			^117^
		BDBV 200706291	10^3^ PFU	IM	40%	Fever, anorexia, macular rash, depression	Models for immune signatures/Expensive and ethical issues	^119^
		MARV Angola/Musoke	10^3^ FFU	IM	100%	Hemorrhagic	Varied susceptibility to differently virulent strains/Expensive and ethical issues	^120,893,894^
	Rhesus monkey	MARV-Ozolin/Ravn/Angola	10^3^ FFU/PFU	IM/Aerosol	0–100%	Fever, lymphadenopathy, anorexia, malaise, edema, dehydration and rash	Varied susceptibility to differently strains, aerosolized MARV exposure model/Expensive and ethical issues	^120,121,893,894^
	Cynomolgus macaque	EBOV Kikwit EBOV Mayinga	10^4^ PFU	Conjunctival	40%~100%	Weight loss, fever, anorexia, and hypothermia	Mucosal (Conjunctival) exposure model/Expensive and ethical issues	^128,133^
				Oral	40%~60%	Ruffled fur, Weight loss, fever, anorexia, malaise hypothermia, petechia, dehydration, rash	Mucosal exposure model, obvious gastrointestinal symptoms in IN exposed group, recapitulating the hallmark features of human disease/Do not reflect the difference in virulence between strains, expensive and ethical issues	^128,133,134^
			64 PFU	IN	100%			
			74 PFU	IM				
			10^4^ PFU	IM				
		EBOV Makona	10^4^/100 PFU	IM/Oral/IN	100%	Malaise, fever, weight loss, rash, lymphadenopathy, anorexia, motor dysfunction, coagulopathy, and hypothermia	Recapitulating the hallmark features of human disease, Mucosal exposure model/Do not reflect the difference in virulence between strains, expensive and ethical issues	^129,132,134^
		SUDV Gulu	10^3^ PFU	IM	100%	Fever, weight loss, malaise, diarrhea, dehydration, bleeding, petechia, anorexia, reduced stool output, rash	Rapid systemic disease/Significant difference in clinical signs among infected macaques	^136,895^
		RESTV	5 × 10^3^ PFU	SC	83%	Anorexia, fever, weight loss, nasal discharge, hemorrhages.	Predict interspecies transmission potential of RESTV/Not recapitulating the hallmark features of human RESTV disease	^145^
		MARV Angola	10^3^ FFU	IM	100%	Severe MHF	Varied susceptibility to differently virulent strains/Expensive and ethical issues	^120,131,150^
		MARV Musoke			50%	Severe MHF		
		MARV Ozolin			0	Fever, hunched posture, and anorexia		
	African green monkey	EBOV	10^3^ PFU	Aerosol	100%	Fever, anorexia, dehydration, lymphadenopathy	Aerosolized model/Lack of rash and behavioral changes	
		SUDV Boniface	500 PFU	Aerosol	100%	Fever, anorexia, malaise, depression, rash, dyspnea, reduced urine and fecal output, dehydration	Aerosolized model/No pronounced changes in hematology	^117^
		EBOV Kikwit	10^6^ TCID_50_	Aerosol	92%	Fever, ruffled fur, malaise, dyspnea, anorexia, hemorrhage	Aerosolized model/Rapid onset of symptoms and death	^152,153^
	Marmoset monkey	EBOV Kikwit	10^3^ PFU	IM	100%	Fever, weight loss, anorexia, depression, and reduced stool output	Small NHP model/Lack of widespread intravascular coagulation	^152,153^
		MARV Musoke						^152^
	Baboon	gpaEBOV	100 PFU	SC	100%	Weight loss, hyperthermia, anorexia, adynamia, ataxia, abasia, rash, diarrhea, dyspnea, and hemorrhages	Recapitulating the hallmark features of human disease/virus adaptation needed	^155^
Surrogate model	Neonatal Mice	rVSVΔG-ZEBOV-GP	10^3^ TCID_50_	SC	100%	Tremors, widened stance, ataxia, seizures and paresis and/or paralysis	Model for neurologic and ocular symptoms, models in BSL-2 conditions/Data cannot be directly extrapolated to humans	^156,158^
	Hamsters	rVSVΔG-ZEBOV-GP	10^3^–10^6^ PFU	IP	33–100%	Weight loss	Models in BSL-2 conditions, model for entry of EBOV or MARV depends on glycoproteins/Detailed pathogenesis of the disease different from EBOV and MARV infection	^159^
		rVSVΔG-MARV-GP	10^5.5^–10^7.5^ PFU		25–100%			

*EBOV* ebola virus, *PFU* plaque forming unit, *IP* intraperitoneal, *IC* intracutaneous, *SC* subcutaneous, *IFN* interferon, *SUDV* Sudan virus, *MARV* Marburg virus, *STAT* signal transducer and activator of transcription, *BDBV* Bundibugyo virus, *TAFV* Taï Forest virus, *RESTV* Reston virus, *FFU* focus-forming unit, *IN* intranasal, *IM* intramuscular, *TCID*_*50*_ 50% tissue culture infective dose, *ma* mouse-adapted, *scida* SCID mouse-adapted, *ha* hamster-adapted, *gpa* guinea pig-adapted, *rg* reverse-genetics, *BSL-2* biosafety level 2, – not applicable

#### Mice

Mice are the most frequently used animal models in preclinical studies. Mice are economical, abundant, well characterized, and easy to manipulate. The wide availability of biochemical reagents and immunological tools further supports the application of mouse models. However, wild-type immunocompetent mice are resistant to filoviruses. Filoviruses replicate poorly and are eliminated in a short period of time in mice. Consequently, filovirus infection in mice did not result in symptomatic or lethal outcomes.^57,58^ In response, newborn mice and immunodeficient mice were established to obtain an effective infection model. In combination with an immunodeficient strategy, mouse-adapted approaches have also been adopted to establish lethal models.

#### Neonatal suckling mice

Neonatal suckling mice are susceptible to multiple viruses due to their immature immune system. EBOV can cause fatal infection in newborn mice after intracranial (IC) or intraperitoneal (IP) injection.^59^ A viral load as low as 1 plaque-forming unit (PFU) could be detected in newborn mice.^60^ However, due to the incomplete immune system of newborn mice, this model is not applicable for pathogenesis studies or vaccine evaluation.

##### Mouse adapted model

Successive passages of wild type (WT) EBOV in mice yielded mouse-adapted Ebola viruses (maEBOVs), which can cause symptomatic infection in wild-type mice. Uniform disease and lethality were achieved only by IP injection rather than intramuscular (IM) or subcutaneous (SC) injection.^61^ When maEBOV was intraperitoneally injected into adult BALB/c, CD-1, or C57BL/6 mice, the animals died at approximately 5–6 dpi, which resembled EVD in NHPs. Further studies revealed that the median lethal dose (LD50) of maEBOV was 0.03 PFU.^62,63^ To investigate the genetic determinants of virulence, recombinant maEBOV and wtEBOV viruses were constructed using reverse genetic approaches. Recombinant viruses harboring NP and VP24 mutations were found to be lethal in mice and resistant to type I IFN in vitro.^63^ Although the VP35 protein of EBOV was found to block type I IFN responses in vitro through multiple mechanisms, it did not affect the virulence of maEBOV in mice.^64^ MaEBOV replicated rapidly in mice, with serum viral titers as high as 10^9^ PFU/ml. Systemic dissemination of the virus can lead to widespread infection and necrosis of the liver, spleen, and other organs in mice. Histopathological and biochemical data showed liver and kidney damage in mice, similar to what has been observed in NHPs.^65^ Massive lymphocyte apoptosis, which is a marker of poor prognosis in patients with EBOV infection and NHPs, was observed in maEBOV-infected mice.^62,65,66^ TNF-α, IFN-γ, IL-8, MIP-1 α, MIP-1 β and proinflammatory cytokines such as MCP-1 were also produced in a pattern similar to that of wtEBOV-infected NHPs.^67,68^ Moreover, lymphocyte activation during lymphoblastoid formation, increased CD44 expression on the surface of T cells, and increased lymphocyte numbers in the blood at later stages of infection were observed in mice. The above results showed that, compared with infection in NHPs, infection in mice caused a similar pathogenesis in maEBOV cells. Like NHPs, mice are suitable models for the study of filovirus-induced coagulopathy. Consequently, anticoagulant treatment of mice infected with maEBOV could demonstrate the role of coagulopathy in EVD pathogenesis. Although the maEBOV-infected mouse model resembled the disease of wtEBOV-infected NHPs in many respects, there are also some differences. Mice were sensitive to maEBOV injected via the IP route.^62^ In addition, fibrin deposition was not observed in tissue sections after maEBOV infection in mice, whereas in NHPs, D-dimers appeared in plasma as a result of fibrin deposition and breakdown, and fibrin deposition could be observed in the spleen of infected animals and other tissues where viral replication occurred.

##### Immunodeficient mouse models

Interferon receptor (IFN-α/βR^−/−^) or cytoplasmic signal transducer and activator of transcription-1 (STAT-1) protein knockout mice are susceptible to filovirus infection. Both types of mice progressed to fatal infection with wtEBOV.^58,69^ Further studies revealed that infection with EBOV Mayinga was lethal in IFN-α/βR^−/−^ mice, whereas infection with EBOV Kikwit did not cause mortality, suggesting that different responses to type I IFNs occur among different isolates of EBOV. Similarly, Sudan virus (SUDV) has been shown to cause differences in lethality in type I IFN-deficient mice.^58,70^ These results suggest that the type I IFN response is critical for the pathogenesis of filovirus infection. When STAT-1^−/−^ mice were infected with five different wild-type filoviruses, SUDV and MARV caused 100% and 80% lethality, respectively. EBOV, BDBV, and TAFV caused 40%, 20%, and no mortality, respectively.^71^ Similarly, MARV-Musoke-infected STAT1^−/−^ mice developed lymphopenia and died within 7 days.^69^ Serial passages of MARVs in SCID mice resulted in mouse-adapted Angola (ma-Ang) and RAVV (ma-RAVV) strains, which were able to infect and cause lethal disease in adult immunocompetent BALB/c mice.^57,72,73^ The single IP challenge route resulted in lethal disease. BALB/c mice infected with ma-RAVV became lethargic and hunched, while no evidence of hemorrhagic symptoms or maculopapular rash was noted, all animals succumbed to infection within 8 days. Moreover, ma-MARV-Ang and ma-RAVV infection in BALB/c mice led to multiorgan failure.^57,73^ This systemic infection was similar to that observed in MVD patients. A total of 11 amino acid mutations were introduced into ma-MARV-Ang compared to wtMARV-Angola; these mutations were distributed in VP40, VP35, GP, VP30, and VP24 of MARV.^74^ Key amino acid changes attributed to lethal disease in BALB/c mice have not yet been clearly defined. The VP40 matrix protein of the MARV Musoke strain and Ravn strain has been proven to antagonize IFN-α/β and IFN-γ signaling by inhibiting the activation of the cellular tyrosine kinase Jak1 in primate cells. However, neither MARV nor RAVV VP40 effectively inhibited IFN signaling in mouse cells. VP40 from maRAVV inhibited IFN signaling in a species-dependent manner.^75^ Two (V57A and T165A) amino acid changes that accumulate in VP40 are responsible for efficient IFN signaling antagonism by RAVV VP40 in mouse cells. wtEBOV can cause fatal infection in SCID mice lacking B and T cells, but the course of disease is prolonged compared to that in other lethal mouse models.^58,72^ After challenge with filoviruses, SCID mice gradually develop progressive weight loss and hypokinesia and subsequently die at 20–25 dpi.^57^ Compared to common mice, immunocompromised mice enabled rapid evaluation of candidate medical therapies using WT filovirus strains without the need for adaptation. However, this model has a high unit cost, requires sterile conditions, and lacks normal immune functions, which hamper the investigation of immune correlates of protection or pathogenesis. Consequently, this model is not applicable for widespread usage.

##### Humanized mice

Humanized mice were obtained using genetic engineering approaches in which a human-like environment was established. Bird et al. generated a humanized mouse model of EBOV infection by implantation of human immune cells (Hu BLT). Hu BLT mice developed EVD upon wtEBOV infection. Infection with high-dose EBOV results in rapid, fatal EVD characterized by high viral loads, alterations in key antiviral immune cytokines and chemokines, and severe histopathological changes. Dose- and donor-dependent clinical features were observed in Hu BLT mice infected with low-dose EBOV Mayinga and Makona isolates.^76^ Similarly, HLA-A2–transgenic, NOD–scid–IL-2γ receptor–knockout (NSG-A2) mice were used to compare the pathogenesis of EBOV and RESTV. Compared to EBOV, RESTV was markedly less pathogenic and killed 20% of infected mice due to exacerbated inflammation and viral replication in the liver. Interestingly, different case fatality rates of Ebolavirus species in humans were recapitulated in humanized mice. Specifically, among the strains of Zaire Ebola virus tested, huNSG-A2 mice were significantly less susceptible to the Makona virus strain than to the Mayinga virus. These results suggested that humanized mice could be a model for the pathogenicity of emerging filoviruses.^77^ The use of collaborative cross (CC) mice further recapitulated EBOV-related disease phenotypes. Exposure of CC mice to maEBOV has yielded a wide variety of outcomes, ranging from complete resistance to lethal disease.^78^ MaEBOV-infected CC mice exhibited typical lesions. As the disease progressed, the CC mice exhibited prolonged blood coagulation, internal hemorrhage, coffee-colored blood, splenomegaly, hepatic discoloration, and a soft texture. Compared to C57BL/6J mice, CC mice exhibit significantly greater thrombin and prothrombin times, which emphasizes that the host genetic background plays a role in disease development. To address the shortcomings of immunodeficient mice and obtain a unique opportunity to study the interactions of filoviruses with human immune cells in vivo, an immunodeficient mouse strain in which the Rag2, γc, and CD47 genes were knocked out was generated using the bone marrow, liver, and thymus (BLT) methods. This model produced human dendritic cells, monocytes, monocyte-derived macrophages, natural killer cells, B cells, and T cells. When these triple knockout BLT (TKO-BLT) mice were IM inoculated with MARV-Angola, symptoms started at 16 dpi and ultimately resulted in partial morbidity.^79^

Overall, mouse models of filoviruses exhibit viremia and a high viral burden in the spleen, liver, blood, and multiple organ tissues. Lymphopenia, thrombocytopenia, renal dysfunction, and liver damage were recaptulated.^73,80,81^ Fluctuations in blood glucose, albumin, globulin, and alanine aminotransferase (ALT) levels were also noted and are consistent with the findings of other models of filovirus infection. As in NHP models, both proinflammatory and anti-inflammatory cytokines are frequently produced in mice, together with lymphocyte activation, increased T-cell CD44, and increased circulating lymphocytes, indicating dysregulation of the immune system.^62,73,80^

#### Syrian golden hamsters

Hamsters are insusceptible to wild-type filoviruses, and virus adaptation is required to establish a sensitive infection.^62^ Hamsters infected with maEBOV developed clinical signs, such as ruffled fur and hypokinesia, and died of the disease at 4–5 dpi. Severe coagulation disorders and thrombocytopenia were also observed in infected animals in the late stages of disease.^82^ High virus titers were detected in the heart, liver, spleen, lungs, kidneys, brain, and blood. Moreover, maEBOV infection caused histopathological changes, including inflammatory cell infiltration, cell necrosis, and apoptosis, which were mainly restricted to the lymphoid organs and liver. Liver lesions, including disseminated hepatocyte degeneration and necrosis, moderate neutrophil counts, slight infiltration of macrophages, and spleen damage, characterized by lymphocyte necrosis and a marked reduction in white marrow, were noted.^28^ However, fibrin deposition was not observed in liver sinusoids.^83^

When STAT2^−/−^ hamsters were inoculated with 10^5^ PFU of MARV-associated musoke via the IP route, they developed clinical signs at 5 dpi, including a scruffy coat, lethargy, a hunched posture, irregular breathing, orbital tightening, nasal discharge, abnormal gait, weight loss, hyperreflexia, and head tilt, and succumbed to the disease at 12 dpi.^84^ High titers of MARV were detected in the blood, kidneys, spleen, liver, lymph nodes, and heart, while moderate titers were detected in the brain.^84^ Splenitis, hepatitis and massive release of cytokines were observed at 6 dpi, indicating a dysregulated immune response. Neither maculopapular nor punctate rashes were found in the infected animals.^84^ Notably, hamsters infected with haMARV, which was obtained by serial passaging of MARV Angola three times in Hartley guinea pigs and then serial passaging five times in hamsters, developed a maculopapular rash with visible petechiae on the face, chin, chest, abdomen, extremities, and severe coagulation disorders, consistent with what has been observed in humans. On autopsy, the liver was covered with necrotic lesions and neutrophilic infiltrates, while the spleen was infiltrated with neutrophils and macrophages, with fibrin deposits in the red marrow.^85^ Unfortunately, the lack of commercial reagents for accurately monitor host immune responses limits the use of hamster models in the study of the pathogenesis and medical countermeasures of EVD.

#### Guinea pigs

Both outbred and inbred guinea pigs are insusceptible or less susceptible to filoviruses and are characterized by transient illness.^86^ To overcome this issue, a lethal model of Strain 13 guinea pigs was established by serial passages of EBOV-Mayinga 4 times.^87^ After viral adaptation and inoculation, guinea pigs presented obvious clinical signs, such as weight loss, anorexia, fever, and dehydration, and died at 8–11 dpi without apparent hemorrhage. Viremia was detected in multiple organs. Abnormal blood biochemical parameters, which reflect liver and kidney damage, were observed. By necropsy, swollen lymph nodes, pale, friable livers, and slightly enlarged spleens, as well as pathological changes, including fluid accumulation in the small intestine and cecum, bruised spots on the surface of the kidneys, distal gastric erosions, and enlarged adrenal glands, were observed. Fibrin deposition occurs in interstitial fibroblasts and endothelial cells in various tissues in the late stage of EBOV infection.^87^ Serial passage of the EBOV strains 7–9 times was uniformly lethal to Hartley guinea pigs.^88,89^ Diarrhea and intestinal hemorrhage presented in the late stage of the disease.^89^ The typical disease features of guinea pigs, such as early infection of macrophages and dendritic cells, apoptosis of bystander lymphocytes, increased levels of proinflammatory cytokines, and abnormal coagulation, are highly consistent with those observed in humans.^90^ Similarly, Hartley guinea pigs showed signs of disease and uniformly succumbed at 9–14 dpi after infection with Gpa-SUDV.^91^

When inoculated with whole blood from MVD patients, Hartley guinea pigs showed reduced appetite, weight loss, fever, and lethargy during the incubation period of 4–10 days. Subsequently, the animals gradually recovered.^92^ After adaptation to Hartley guinea pigs, whole-blood guinea pigs were adapted (GPA) to MARV, causing uniform lethality, febrile illness, and clotting abnormalities. GPA MARV-infected Hartley guinea pigs succumbed to the disease at 6–9 dpi, and infectious viruses were detected in the liver, kidneys, lungs, and spleen. Compared to GPA MARV Ang pigs, GPA MARV-Ravn-infected guinea pigs presented more obvious weight loss, greater body temperature, and fewer tissue lesions.^93^ Strain 13 guinea pig-adapted MARV variants, namely GPA-MARV Musoke and GPA-MARV Ravn, were also developed.^94^ Both adapted variants caused viremia and clinical signs in Strain 13 guinea pigs. However, only GPA-MARV Ravn was uniformly lethal in Strain 13 guinea pigs.^94^ Compared to mice and hamsters, guinea pigs are timid and stressed, which poses obstacles in housing and handling; additionally, they have a high unit cost and lack reagents to characterize aspects of immune responses, indicating that they are more suitable as a secondary animal model for confirming experimental results and trends from mouse/hamster studies.

#### Ferrets

The domestic ferret (*Mustela putorius furo*) is a member of the genus *Mustela* in the *Mustelidae* family.^95^ Ferrets are naturally susceptible to EBOV. EBOV-infected ferrets exhibited obvious fever and weight loss at 3–4 dpi and died at 7 dpi. Other clinical signs, such as progressively worsening depression, diarrhea, dehydration, nasal and ocular discharge, labored breathing, hunched posture, and altered gait, were also observed. The most common lesions in ferrets were lymphohistiocytic, neutrophilic necrotizing hepatitis, and necrotizing splenitis.^96^ To investigate the pathogenicity of EBOV, EBOV-Makona was rescued by reverse genetic approaches, and the resulting strain was known as rgEBOV-C07. rgEBOV-C07 was highly pathogenic in ferrets. All animals succumbed to the disease at 6–7 dpi after infection with 0.1 PFU of rgEBOV-C07 via the IM route. In the nonlethal challenge groups, symptomatic infections persisted until 15 dpi. The LD_50_ of rgEBOV-C07 in ferrets was 0.015 PFU.^97^ Biochemical results in infected animals revealed increases in liver markers, ALT, alkaline phosphatase (ALP), and BIL; renal markers, BUN, and CRE; and decreased levels of ALB, indicating liver damage, kidney dysfunction, and edema. Additionally, ferrets recapitulated disseminated intravascular coagulation, with prolonged activated partial thromboplastin time (APTT) and thrombin time (TT), increased fibrinogen levels, and decreased prothrombin activity percentage (PT). Multiple organ injuries with uncontrolled virus replication were observed in infected animals, including the kidneys, liver, spleen, and lungs.^98^ There are also investigations of mucosal challenge routes, including oronasal, oral, and ocular inoculation. Animals administered at target doses of 1, 10, or 100 PFU via the oral-nasal or oral route died at median times of 152, 136, and 126 h, respectively. All animals infected via the ocular route survived for 28 days; however, when the study was terminated, the ferrets presented with mild or no symptoms.^99^

When ferrets were challenged with SUDV at a dose of 1000 TCID_50_ via the IM or IN route, signs of illness were observed at 4 dpi and included fever, weight loss, viremia, multiple-organ dysfunction, viral shedding, and death. SUDV infection induced a decrease in the serum ALB and calcium concentrations and an increase in the globulin, ALP, ALT, and amylase (AMY) levels. In plasma, increased fibrinogen, APTT, and TT and decreased PLT and PT% were observed, indicating disseminated intravascular coagulopathy.^100^ In another study, typical symptoms, including hemorrhage and rash, which are also observed in humans, were observed in ferrets.^96^ When the ferrets were challenged with BDBV, common signs such as fever, weight loss, hypothermia, euthanasia, viremia and virus shedding, rash and liver, and renal and pancreatic damage were observed. Compared with EBOV- and SUDV-infected animals, ferrets infected with BDBV died of disease within a mean period of 8–9 days, indicating a prolonged disease course.^96^ The disease course observed in BDBV-infected ferrets was similar to that found in NHPs.^98^ Despite asymptomatic infections in humans, RESTV is highly lethal to both humanized mice and NHPs, which are at potential risk of introducing mutations and causing the emergence of the human-pathogenic RESTV.^77,101,102^ As in cases of EBOV and SUDV infection, RESTV infection in ferrets was associated with similar signs of disease, including common signs of disease and abnormal hematological parameters. alterations in plasma biochemistry markers, viremia, viral shedding, and histopathological changes in multiple organs.^103^ In comparison, TAFV does not cause lethal infection in ferrets.^104^ Additionally, infection with MARV or ravn virus (RAVV) did not cause obvious signs in adult or naive ferrets.^105,106^ The above results demonstrated that ferrets are a naturally susceptible animal model for filovirus infection and recapitulated some disease parameters in humans.

#### Nonhuman primates

NHPs are considered the gold standard models for multiple pathogens due to their similar physiological characteristics and immune regulation. Preclinical rhesus monkeys and cynomolgus macaques are the most frequently used models of EBOV infection. In addition, African green monkeys, marmoset monkeys and baboons were included.

##### Rhesus monkeys

Rhesus monkeys (RMs) can precisely imitate the clinical features of human EVD, and are characterized by hemophagocytic and lymphohistiocytosis/macrophage activation syndrome.^64^^,107^ After intramuscularly injecting 10^3^ PFU of EBOV, the RMs were febrile, which typically started at 2–4 dpi, peaked at 41 °C, and then drcreased sharply before death. Additionally, infected RMs exhibited anorexia, dehydration and decreased activity and subsequently lost more than 10% of their initial weight. Skin rashes were noted in all RMs at 4–7 dpi. Most animals succumbed to EBOV infection at 5–9 dpi.^83,108^ In some cases, diarrhea, bleeding from nose puncture points, gums, the rectum, and the vagina were observed in infected animals.^83,108^ Persistent intraocular Ebola virus RNA was associated with severe uveitis in a convalescent rhesus monkey.^109^ Blood analysis of infected animals revealed significant reductions in hemoglobin and hematocrit, together with a decrease in C-reactive protein (CRP) and increases in fibrinolytic degradation products, plasminogen activator inhibitor-1, and tissue type plasminogen source activators. Additionally, increased leukocyte counts, decreased platelet counts, lymphocyte counts, CD8^+^ T-cell counts and natural killer (NK) cell counts and extensive bystander cell apoptosis in peripheral blood monocytes and lymphoid tissues were observed.^66,110–112^ In the late stage of EVD, aspartate aminotransferase (AST), alanine aminotransferase (ALT), alkaline phosphatase (ALP), gamma-glutamyltransferase (GGT), serum creatinine (SCre) and blood urea nitrogen (BUN) concentrations increased, while the total serum protein concentration decreased.^111,113^ In addition, increased serum concentrations of proinflammatory cytokines and chemokines were detected.^83,114^ During necropsy, lymph node enlargement accompanied by congestion, bleeding and edema was noted. The mesenteric lymphatic tissue was congested with erythema, and duodenal bleeding was observed. The liver was enlarged and fragile, with a grid shape and rounded edges. Multifocal or concomitant bleeding was observed in the bladder. Bleeding, congestion, and fibrin deposition were found in the liver, kidneys, and spleen.^115^ SUDV- or BDBV-infected RMs exhibit histopathological manifestations similar to those observed in EBOV infection.^116^ The disease course in patients with SUDV infection was slightly later than that in patients with EBOV infection. When infected with SUDV at a dose of 10^3^ PFU, the RMs succumbed to the disease at 7–10 dpi, with an average of 8.3 ± 1.3 days;^116^ in some cases, the disease duration was 11–15 days or even 17 days.^117^ An equal dose of BDBV caused 40% of the RMs to die at 13–19 dpi. A large number of virions were detected in the liver, lung, and spleen of EBOV-infected RMs, whereas in SUDV-infected RMs, few virions were detected in only the liver.^118,119^ After inoculation with MARV-Angola via IM injection at a dose of 10^3^ PFU, RMs developed fever, lymphocytopenia, leukocytosis, anorexia and rash at approximately one week, and a few groups of individuals developed thrombocytopenia at 7 dpi.^120,121^ All animals succumbed to the disease at 7–8 dpi. Liver damage and elevated alanine transaminase (ALT), aspartate transaminase (AST), total bilirubin, and gamma-glutamyl transpeptidase (GGT) levels were observed. Increased D-dimer concentrations and decreased C-protein activity indicate coagulation dysfunction. Viremia was detected at 3 dpi and peaked at 6 dpi. MARV was highly expressed in the spleen and liver, as well as monocytes, macrophages, and fibroblasts from other organs. Pathology revealed reticular liver and liver discoloration, hepatocyte degeneration, and necrosis.

##### Cynomolgus macaque

Cynomolgus macaque (CM) is another NHP model that accurately recapitulates the hallmarks of filovirus infection in humans.^122,123^ Compared with RMs and African green monkeys (AGMs), CMs were more susceptible to EBOV and SUDV and presented shorter survival times, earlier onset of viremia and greater viral loads.^116,117^ Host transcriptional characteristics are correlated with clinical signs and corresponding organ damage, particularly in severe EVD patients.^124,125^ The distinct transcriptional responses to virus infection in NHPs may explain the differences in disease manifestations and viral replication among primates. The significant upregulation of IRF1, BST-1/2, TLR4, and BCL6, which play roles in limiting virus spread, resulted in a delayed disease course in RMs. In challenged CMs, the significant downregulation of genes, including CD3G, CD3E, ZAP70, CD8B, and IL7R, which are indicators of T-cell loss, and CCNY, CHD9, SHPRH, and TPI1, which play roles in cell division and nucleic acid metabolism, indicated more severe cell injury and stress, which predict low-level neutralizing antibody responses and T-cell-mediated antiviral responses.^126,127^ In a study investigating mucosal exposure to EBOV in CMs, IN-exposed CM models were found to be uniformly lethal and correlated with significantly delayed times to death compared to exposure via the IM route.^128,129^ The prolonged time from challenge to death in IN-exposed animals accurately reflects the time frame in humans.^128^ The clinical manifestations and gross pathological features of the infected animals were similar between these two exposure routes and included weight loss; increased rectal temperature, GGT, and BUN; and decreased serum ALB and RBC counts, except for less dramatic and delayed increases in ALP and ALT and more variable viremia in the IN group. Gastrointestinal (GI) tract pathology, which recapitulates gastrointestinal symptoms observed in humans, was more frequently observed in the IN-exposed group than in the control group.^128^ The differences in disease course between the two groups may be due to the lower percentage of permissible initial target cells in the upper respiratory tract than in muscle tissue.^129^ Low doses of EBOV-Kikwit or Mayinga via the IM or aerosol route cause severe clinical signs and uniform lethality in CMs.^130,131^ However, except for low-level virus replication, no clinical manifestations of EVD were observed in macaques infected with EBOV-Makona at a dose of 10 PFU via the oral or conjunctival route. A high dose of EBOV-Makona was required for the oral or conjunctival route to produce a lethal disease in CMs.^132^ Oral challenge of CMs with EBOV Kikwit resulted in an overall mortality rate of 50%. Animals challenged with a target dose of 10^2^ PFU or 10^4^ PFU of the virus via the conjunctival route showed 40% and 100% mortality, respectively.^133^ Infected animals developed clinical signs, including weight loss, fever, and hypothermia. In addition, persistent viral loads in the eye were observed in NHPs challenged via the conjunctival route. The above results paved the way for research into the transmission of EBOV disease, including early mucosal infections and the establishment of persistent viral infections from NHPs. In addition to the exposure route, strain-dependent clinical courses were also investigated in CMs.^134,135^ The time from exposure to death was 2 days later in the EBOV-Makona-infected cynomolgus macaques than in the EBOV-Mayinga-infected animals. Compared to the systemic rash observed in EBOV-Mayinga-infected animals, the rash observed in EBOV-Makona-infected animals was restricted to the arms, legs, chest and face. A decrease in liver enzyme levels and a greater increase in IFN-γ levels were detected in the EBOV-infected group.^134^ After exposure to the SUDV strain Gulu, CMs succumbed to the disease at 7–8 dpi, with detectable viral RNA and infectious particles.^136^ Common manifestations as well as hallmark symptoms, such as rash, vomiting, diarrhea, hemorrhage, gastrointestinal ulceration and multiple-organ failure, were observed.^137^ In humans, high levels of proinflammatory cytokines and chemokines are observed during acute SUDV infection and are correlated with disease severity.^138,139^ Accordingly, upregulated expression of IP-10, IL-6, MCP-1, MIP-α, and MIP-β was detected in SUDV-infected CMs from patients with middle- and late-stage disease.^116,136^ Furthermore, increased ALT, AST, ALP, GGT, BUN, and CRE levels were indicative of liver and kidney injury. The level of activated platelet-produced sCD40L could reflect the repair of damaged endothelial cells by platelets.^140^ The insertion of an extra uridine residue at the glycoprotein (GP) editing site during the passage process results in a mutant virus with an 8U residue. Different clinical signs were observed in cynomolgus macaques challenged with 7 U or 8 U of EBOV/SUDV.^141,142^ The “7U” virus produced secreted nonstructural GP (sGP), which appears to play a role in immune escape, whereas the “8U” virus produced GP. Decreased sCD40L levels were observed in severe patients, while high levels of sCD40L were observed in survivors. An increase in GP was detected, which was consistent with the increase in viral RNA and infectious particles in exposed animals that succumbed to disease.^143^ Therefore, sCD40L and sGP could be novel biomarkers for characterizing EBOV-challenged models. The mortality rate of BDBV was 66–75% in cynomolgus macaques.^144^ Macaques challenged with BDBV via the IM route succumbed to the disease at 10–11 dpi, which was longer than that observed in EBOV-infected macaques. Clinical signs included maculopapular rash; increased PT, aPTT, ALT, ALP, and BUN; and decreased ALB. RESTV naturally infects cynomolgus macaques and caused an outbreak in the Philippines in 1996. Infected macaques developed clinical signs, including fever, weight loss, and abrupt anorexia at 4–5 dpi, followed by viremias at 5–6 dpi. The period from exposure to death was 8–14 days.^145^ Moreover, the biochemical parameters of RESTV-infected macaques were similar to those of macaques infected with EBOV or SUDV.^146,147^ In addition, histopathological analysis revealed injuries in the spleen, liver, and kidney.^148^ Taï Forest virus caused 60% lethality in NHPs.^149^ After TAFV infection, cynomolgus macaques develop fever, reduced responsiveness, weight loss, anorexia, and viremia and reach the euthanasia criteria at 10–12 dpi. After exposure to MARV-Angola, cynomolgus macaques exhibited typical clinical signs of MVD and succumbed to the disease at 8–9 dpi. Infectious viruses and viral RNA were first detected at 3 dpi and increased rapidly until euthanasia. There were significant increases in ALT, AST, BUN, and creatine (CRE) levels.^150^ Additionally, decreased lymphocyte and platelet counts and prolonged PTT were also observed in exposed macaques.^143^

##### African green monkeys

In African green monkeys, EBOV initially infects monocytes and macrophages and subsequently spreads to hepatocytes, adrenal cortical cells, fibroblasts, and endothelial cells.^151^ Infected monkeys had depleted B-cell follicles and spleen lymphoid cells and died of the disease at 6–8 dpi. Autopsy revealed impaired microcirculation due to fibrin clumps and thrombi deposits in the organs and necrosis of the liver, spleen, and kidneys. After peritoneal infection with 10^4^ LD_50_ of EBOV, all African green monkeys died at 6 dpi with diarrhea and intermittent black stool, similar to what was observed in RMs. Infectious viruses were detected in the blood, heart, lungs, liver, spleen, adrenal glands, kidneys, mesenteric lymph nodes, and urine.^117^ Compared to cynomolgus macaques, African green monkeys are less sensitive to EBOV and do not produce the typical skin rash, which limits their application as an animal model for viral infection.

##### Other NHPs

When inoculated with 10 or 10^3^ PFU of EBOV via the IM route, marmoset monkeys developed severe systemic diseases similar to those observed in patients.^152^ Marmosets showed symptoms of anorexia and weight loss at 3 dpi, followed by depression, decreased stool, weight loss, and death at 4–5 dpi. Blood analysis revealed increased ALT, ALP, and GGT levels; decreased platelet counts; and increased neutrophil counts. Histopathological results revealed disseminated intravascular coagulation (DIC) and multifocal to concurrent hepatic necrosis. Like African green monkeys, marmosets lack the typical signs of EBOV infection.^153^

Baboons infected with EBOV presented abnormal blood parameters, increased hepatic vascular permeability, and impaired hepatic cell function.^151^ Most baboons suffer bleeding and vomiting, accompanied by bleeding from the rectum, vagina, skin, and mucous membranes.^154^ DIC appeared in infected baboons, with rapid hypercoagulation in the early stage of infection, peaking at 4 dpi and then decreasing to hypocoagulation. Before death, the lymphocyte and platelet counts decreased to 15.2% and 67%, respectively. Pathological examination of organs in the advanced stages of infection revealed numerous hemorrhagic sites of varying sizes in the liver and spleen, but no fibrin or thrombin in the vascular lumen and no extravascular deposition of fibrin. Ignatiev et al. infected three baboons with the guinea pig-adapted strain EBOV at a dose of 10^2^ PFU via the SC route.^155^ Viremia was detected at 3 dpi, but clinical symptoms were not observed until 6 dpi. The animals succumbed to the disease at 10–11 dpi. These animals experienced a period of initial hypercoagulability, followed by hypocoagulability at 7 dpi and recovery at 9 dpi.

Compared to other NHP species, cynomolgus and rhesus macaques are the best animal models available due to their susceptibility to EBOV infections. In contrast, African green monkeys and baboons infected with EBOV exhibit several typical disease features, such as abnormal coagulation.^151,155^ In contrast to those in small animal models in which infection was established solely by IP injection, the routes of infection in NHPs were more consistent with those in humans.

#### Surrogate models

Surrogate models are generally developed using viruses from the same family or genus with lower biosafety levels or model viruses. A surrogate model of EVD was established based on replication-competent recombinant vesicular stomatitis virus (rVSV) pseudotyped with the envelope glycoproteins (GPs) EBOV and MARV.^156,157^ In 3-day-old C57BL/6 mice, infection with 10^3^ TCID_50_ of rVSVΔG-EBOV-GP via the SC route caused transient viremia and neurological symptoms, such as tremors, widened stance, ataxia, seizures and paresis, as well as high viral titers in the eyes and brain. These neonatal mice died at 15 dpi.^156^ Severe retinitis was caused by infection of the inner layers of the retina by recombinant virus. When rVSVΔG-EBOV-GP was used to infect neurons in the granular and Purkinje layers of the cerebellum, it caused increasing foci of neurodegeneration and death. In addition, after treatment with the human polyclonal anti-EBOV-GP antibody SAB-139, decreased viral titers, microglial loss, cellular infiltration, and inflammatory responses in the central nervous system and increased survival rates were observed in infected mice.^158^ Hamsters intraperitoneally inoculated with rVSV/EBOV or rVSV/MARV showed disease signs and died within 4 dpi. Recombinant viruses were detected in multiple organs, including the liver, spleen, kidney, and lungs, of infected hamsters, indicating acute and systemic infection and resulting in fatal outcomes. The therapeutic effects of EBOV NAbs were validated in this model.^158,159^

### Medical countermeasures for filovirus diseases

Due to the high case fatality rate of filovirus diseases, preventive and therapeutic approaches have been widely investigated in preclinical trials. Among them, multiple vaccines and therapies for EVD have entered clinical trials, several of which have been approved while no medical responses have been approved for MVD. Here, we focus on approved or cutting-edge approaches for the prevention and control of filovirus diseases (Table 3).

**Table 3:** Representative medical countermeasures for filoviruses

Classification	Manufacturer	Name	Platform/Strategy	Stage	Efficacy/Benefic	References
Vaccines	Merck	Ervebo	Encoding the GP of EBOV based on VSV	Licensed	100% overall protective efficacy	^166^
	CanSinoBIO	Ad5-MakGP	Encoding the GP of EBOV based on Ad5	Licensed	Tolerate and immunogenic	^169–171^
	NIAID and Okairos	cAd3-EBO	Encoding the GPs of EBOV and SUDV based on ChAd3	Licensed	Tolerate and immunogenic	^174^
	Bavarian Nordic	MVA-BN Filo	Expressing GPs of filovirus based on MVA vector	Licensed	Long-lasting immune response	^176^
	Russia	rVSV +Ad5-EBOV	Prime-boost strategy of two viral vector vaccines	Licensed	Quickly awaken of immune memory and a robust immune response	^177^
Antibodies	Mapp Biopharmaceutical	ZMapp	Antibody cocktail contains of three different monoclonal antibodies	Licensed	Tolerated and showed promise efficacy	^191,193^
	Regeneron Pharmaceuticals	REGN-EB3 (Inmazeb)	Cocktail of three fully human monoclonal antibodies	Licensed	Reduce fatality rate in clinical cases	^192,195^
	Ridgeback Biotherapeutics	mAb114	Monoclonal antibody isolated from a survivor	Licensed	Binds to conserved region and was safe in clinical	^197^
	University of T exas Medical Branch	MR191-N	Isolated from MARV convalescent patient	Preclinical	80%-100% protective efficacy in NHPs	^201,202^
Small molecular drugs	Biocryst	Galidesivir (BCX4430)	Adenosine analog	Clinical	83%-100% protective efficacy in NHPs	^203^
	Toyama chemical	Favipiravir (T-705)	RNA polymerase inhibitor	Clinical	Protect against EBOV and MARV in animal models	^204,205^
	Gilead technology	Remdesivir (GS-5734)	Adenosine analog	Clinical	Showed protective efficacy in nurses and infants with EVD	^206–208^
Oligonucleotides	University of Washington	AVI-6003	Oligonucleotide analogs that inhibit mRNA	Clinical	Protective in NHPs and tolerate in humans	^209–211,896^
siRNAs	Tekmira Pharmaceuticals	NP-718 m	Interferes with mRNA translation	Preclinical	Protective in guinea pigs and NHPs	^212,213^

*siRNAs* small interfering RNA, *GP* Glycoprotein, *EBOV* Ebola virus, *VSV* Vesicular stomatitis virus, *Ad5* Adenovirus type 5, *SUDV* Sudan virus, *ChAd3* Chimpanzee adenovirus type 3, *MVA* Modified vaccinia virus Ankara, *MARV* Marburg virus, *NHPs* Nonhuman primates, *EVD* Ebola viral disease

#### Preventive vaccines for filovirus diseases

Three viral vector-based vaccines have been approved for prevention of EVD. Ervebo (V920) was developed by Merck and is also known as rVSV-ZEBOV. It is a recombinant VSV-based EBOV vaccine candidate in which the VSV G gene was replaced with that of Zaire Ebola virus (ZEBOV) to obtain a recombinant virus. According to preclinical studies, a single dose vaccination with rVSV-ZEBOV was safe, high immunogenic and fully protected both mice and NHPs against lethal challenge with EBOV.^160,161^ Innate antiviral responses induced by vaccination are responsible for rapid protection.^162^ Subsequently, the safety, immunogenicity and protective efficacy of rVSVΔG-ZEBOV have been investigated in several clinical trials. In human recipients, EBOV-specific antibodies appeared at 14 dpv, peaked at 28 dpv, and were maintained for more than 2 years.^163–165^ In a large-scale Phase III Guinea ring vaccination trial, substantial protection from EVD was achieved in rVSV-ZEBOV recipients. A single dose of 2 × 10^7^ PFU of rVSV-ZEBOV was tolerated and immunogenic in volunteers, corresponding to an overall protective efficacy of 100%.^166^ In 2019, rVSV-ZEBOV was approved by the European Medicines Agency (EMA) and has been licensed for emergency use by the FDA.^167^ In 2023, the expanded indications of Ervebo have been approved by the FDA. Now, Ervebo is applicable for individuals older than 12 months.

Directed against the epidemic strain in 2013–2016, Ad5-MakGP, which contains a recombinant Adenovirus type 5 (Ad5) expressing the GP of EBOV Makona strain, was developed by CanSinoBIO. In NHPs, a single dose of Ad5-MakGP provided sterile immunity and protected all animals from lethal challenge.^168^ In Phase I clinical trial, Ad5-MakGP was tolerated and immunogenic. To some extent, humoral and cellular immune responses are blunted by the presence of anti-vector immunity.^169,170^ Boosting immunization with Ad5-MakGP at 6 months after the primary immunization resulted in robust immune memory and humoral immune responses.^171^ Notably, tolerability, immunogenicity and immune response persistence varied among different races.^172^ For example, the duration of the immune response in African participants was shorter than that in Chinese participants. This phenomenon has also been observed in clinical trials of rVSV-ZEBOV.^164^ These results highlight the need to include ethnicity-related factors in clinical trials.

Based on chimpanzee adenovirus type 3 (ChAd3), a bivalent vaccine was constructed that encodes the GPs of EBOV and SUDV, termed cAd3-EBO. In parallel, compared with chimeric adenovirus type 63 (ChAd63) and (MVA) vaccine, cAd3-EBO induced superior immune responses and conferred uniform protection against EBOV challenge in macaques.^173^ cAd3-EBO entered Phase I clinical trial and was proven to be safe and immunogenic.^174^ A ChAd3-based monovalent vaccine encoding the GP of ZEBOV was also constructed, namely ChAd3-EBO-Z. In a Phase I clinical trial, after a single-dose vaccination, antibody titer induced by ChAd3-EBO-Z was slightly lower than those induced by rVSV-ZEBOV.^175^ MVA-vectored vaccine candidates expressing ZEBOV GP, SUDV GP and MARV-Musoke GP, termed MVA-BN-Filo, which confer long-lasting protection, were also investigated.^176^

Several heterologous prime-boost strategies that induce potent immune responses have also been developed. Based on the positive results obtained from rVSV-ZEBOV and Ad5-EBOV, a heterologous prime-boost strategy was developed based on these two vaccine platforms. In Phase I/II clinical trials, the heterologous prime-boosting vaccination rVSV-ZEBOV+Ad5-EBOV quickly induced the awaking of immune memory and a robust immune response.^177^ Moreover, this strategy alleviated the impact of anti-vector immunity. In December 2015, Russia approved the registration of these approaches. There have also been attempts to boost DNA vaccines with Ad5-EBOV. In cynomolgus macaques, vigorous cellular/humoral immunity and full cross-protection were achieved in vaccinated animals.^144,178^ Additionally, boosting cAd3-EBO with an MVA-vectored vaccine conferred long-lasting protection.^173^ When ChAd3-EBO-Z was boosted with MVA-EBO-Z, the levels of virus-specific antibodies and CD8^+^ T cells increased by 12 and 5 times, respectively. Virus-specific antibody responses in participants primed with ChAd3-EBO-Z remained positive at 6 months post immunization but were significantly lower than those in participants who received the MVA-EBO-Z booster.^175^ In addition, a prime-boost strategy involving ChAd3-EBO-Z and MVA-BN-Filo was shown to trigger immune responses that were maintained for over 12 months.^179,180^

Some studies have attempted to elucidate the correlations of immune protection of EBOV vaccines. Depletion of CD8^+^ cells in vivo abrogated the protection against the lethal challenge of EBOV, while passive antibody transfer from vaccinated animals to naive macaques failed to confer protection.^181^ These results indicated that CD8^+^ T cells play a major role in vaccine-induced immune protection against EBOV infection, but antibodies are not sufficient to confer protection. Overall, acute protection was strongly associated with antibody responses, while long-term protection required the generation of both effector and memory CD8^+^ T-cell responses and cytokines.

In a similar manner, vaccines for MVD were developed mainly based on VSV, cAd3, MVA and DNA platforms, which have been proven to be immunogenic in NHPs and are under investigation in clinical trials.^182^ DNA vaccines for filoviruses have shown good safety in NHPs and potent immune responses. However, in clinical trials, the immunogenicity and benefits of these vaccines are limited.^183,184^ DNA vaccines expressing MARV-Musoke GP and MARV-Angola GP were tested in cynomolgus monkeys. Although IgG responses were generated and protection was conferred, clinical symptoms were observed in all challenged animals.^185^ In response, the DNA-Adv prime boost strategy optimized the protective efficacy.

Multivalent vaccines for panfiloviruses are highly important for multiple pathogens overlap areas. rVSV vectored vaccines expressing different foreign proteins could be inoculated simultaneously without interference from each other.^186^ Consequently, VSV vectored vaccines were applied as multivalent vaccines for filoviruses. Tetravalent vaccines against SUDV, ZEBOV, Cote d’Ivoire Ebola virus (CIEBOV) and MARV have been developed.^149^ In cynomolgus monkeys, protection against the above four filoviruses was conferred. Similarly, tetravalent VSV-vectored vaccines expressing glycoproteins from LASV, EBOV, MARV and SUDV achieved 100% protection against hemorrhagic fever after two-dose vaccination.^187^

#### Therapies for filovirus diseases

During the outbreak of EBOV in West Africa, several potential therapies, including antibodies, small-interfering RNAs, convalescent plasma or whole blood, and small-molecule inhibitors such as favipiravir, were tested in clinical trials.^188^ Four investigational drugs, the monoclonal-antibody cocktails ZMapp and REGN-EB3, a single monoclonal antibody (mAb), MAb114, and remdesivir, a small-molecule antiviral drug, were given to hundreds of patients during the Ebola outbreak in the Democratic Republic of the Congo under the Monitored Emergency Use of Unregistered and Investigational Interventions (MEURI) framework and in a randomized clinical trial.^189,190^

There are two FDA-approved antibody therapies for EVD. ZMapp was optimized based on two previous antibody mixtures, one from MB-003 (human or human-mouse chimeric monoclonal antibodies c13C6, h13F6 and c6D8) and two from ZMab (mouse monoclonal antibodies m1H3, m2G4 and m4G7), which have been shown to reverse Ebola virus disease in rhesus macaques after challenge.^191^ Likewise, the ZMapp antibody cocktail contains three GP-targeting antibodies, two of which are GP-specific (c2G4 and c4G7) and one of which is a GP/sGP cross-reactive antibody (c13C6).^192^ During the 2013–2016 West African EVD pandemic, the WHO considered the use of investigational products in an effort to increase access to effective therapies for EBOV infections.^189^ Accordingly, after receiving consent from the appropriate authorities, ZMapp was first given to two American missionaries in Liberia in 2014. The missionaries had contracted EBOV infection and had fallen quite unwell while providing patient care. Both patients survived EVD, and decreased viremia was observed.^193^ In a randomized, controlled trial during the later stages (2015) of the outbreak, a high survival rate (78%) was observed in people treated with ZMapp.^194^

REGN-EB3 (Inmazeb) is a cocktail of three fully human monoclonal antibodies, REGN3470 (atoltivimab), REGN3471 (odesivimab), and REGN3479 (maftivimab), which bind to different glycoprotein regions.^192,195^ These three antibodies bind to nonoverlapping epitopes, including a potentially new protective epitope. REGN3471 binds nearly perpendicular to the viral surface, close to the GP structure’s head, and may even be exposed to the glycan cap. REGN3479 binds to a region between the promoters of GP1/GP2 at the trimer base, and REGN3470 binds to a region outside the glycan cap. REGN3479 is a neutralizing antibody that prevents viral entry. REGN3471 is a nonneutralizing antibody that activates antibody-dependent effector actions, which attract immune cells to the virus. REGN3470 combines both neutralization and effector functions. mAb114 (Ebanga, Ansuvimab) is an FDA-approved single mAb isolated from memory B cells of two patients who survived the EBOV outbreak in Kikwit in 1995.^196,197^ Recently, mAb114 has been the only protective antibody used as monotherapy in macaques. mAb114 combines both neutralization and effector functions. For optimal results, 50 mg/kg of each component of REGN-EB3 was delivered a single IV infusion^198^. During the 2018 Ebola epidemic, an umbrella trial was conducted in the Democratic Republic of Congo, and a polymerase inhibitor (remdesivir), two mAb combinations (REGN-EB3 and ZMapp), and a single human mAb (mAb114), were rolled out under the Monitoring Emergency Use of Unregistered Interventions (MEURI) protocol.^199^ The results showed that 66% of the 154 patients who received REGN-EB3 were still alive at 28 days.^199,200^ In addition, 65% of patients who received mAb114 survived. ZMapp and remdesivir had case fatality rates of 85% in the patient group with a high viral load, while REGN-EB3 and mAb114 had CFRs of 64% and 70%, respectively. Overall survival increased in all treatment groups when the viral load was low, with CFRs of 25%, 29%, 10%, and 11%, for ZMapp, rRemdesivir, REGN-EB3, and mAb114, respectively.

For MARV, a panel of neutralizing mAbs have been isolated from B cells of a MARV convalescent patient. These mAbs are thought inhibit the binding of the GP1 RBD to NPC1 receptors.^201^ Several mAbs have been demonstrated to be effective in mice and rhesus monkeys; these mAbs bind to the same major antigenic site on the MARV GP and some have been shown to cross-react with the RAVV GP. Of which therapeutic given of MR191-N conferred a 100% protective rate. In another study, MR191-N showed protective efficacy of 80 and 100%, against MARV and RAVV, respectively.^202^ Overall, mAb MR191-N is a promising candidate treatment for MARV.

Several small molecule drugs for treating EBOV and MARV have been tested. Galidesivir (BCX4430) is a potential broad-spectrum antiviral drug developed by Biocryst. It is a synthetic adenosine analog that inhibits viral RNA polymerase through a nonspecific RNA strand terminator, which blocks the replication of the RNA genome and inhibits virus propagation.^203^ BCX 4430 inhibited the replication of MARV in cells. In addition, in MARV-challenged cynomolgus monkeys, treatment with BCX 4430 resulted in a survival rate of 83–100%. No obvious symptoms of viral infection were found in the treated animals, and laboratory indices improved, while all animals in the control group died.^203^ The Phase 1 clinical study of this drug was finished in 2016, and the results have not yet been published. Favipiravir (T-705) was developed by Toyama Chemical in Japan and is another RNA polymerase inhibitor. This drug has broad-spectrum antiviral activity against a variety of RNA viruses, and has been approved for the treatment of influenza in Japan. In previous studies, T-705 was shown to be effective in protecting against EBOV in a mouse model.^204^ It was demonstrated that when T-705 was intravenously administered after MARV challenge, a survival rate of 83% was achieved in cynomolgus monkeys. Oral administration showed no protective efficacy.^205^ Remdesivir (GS-5734), developed by Gilead Technology, is a prodrug for adenosine analogs. This drug has been successfully used to treat EVD in NHPs and has been used in nurses and infants with EVD. It has been proven to provide protective effects.^206,207^ In rhesus macaques, GS-5734 protected animals in a dose-dependent manner, with a protective effect of 50–83%.^208^ Positively charged phosphodiester morpholino oligomers (PMOs) are a class of oligonucleotide analogs that inhibit mRNA through steric hindrance of translation, thereby inhibiting viral replication. Functionally, the ribobase is replaced by a structurally similar morpholino, and methylene phosphate diester bonds bind to mRNA, which in turn prevents mRNA translation, while the addition of piperazine residues to PMOs provides a positive charge that can enhance the interaction with negatively charged mRNA. Combinatorial PMO drugs, namely AVI-6003, which consists AVI-7287 and AVI-7288, which target the VP24 and NP mRNAs of MARV, respectively, were prepared. In NHPs, AVI-6003 provided complete protection. However, a subsequent study showed that ACI-7287 treatment did not improve survival or reduce the viral titer, although it inhibited the synthesis of NPs.^209,210^ In clinical trials, AVI-6003 was well tolerated.^211^ Based on the area under the curve of the NHPs and 24 h drug duration, the human protective dose was 9.6 mg/kg, while the protective dose supported by Monte Carlo simulation was 11 mg/kg.^210^

Small interfering RNAs (siRNAs) are emerging antiviral agents. siRNA interferes with mRNA translation by spatially blocking or triggering DNA/RNA double strand breaks. MARV NP targeting siRNA (NP-718 m) was identified. When encapsulated with lipid nanoparticles, NP-718 m inhibits MARV replication in vitro, enters cells through fusion with the endomembrane, and shows extensive protective effects on three Marburg virus strains (Angola/Musoke/RAVV) in guinea pigs.^212^ In rhesus monkeys, NP718-LNP protected all treated animals against the lethal challenge of MARV-Angola.^213^

## Lassa fever

### Etiology, epidemiology, and pathogenesis of Lassa virus

Lassa virus (LASV) is the causative agent of Lassa fever (LF) and it is a human pathogen of the Arenaviridae family that is transmitted to humans by the rodent reservoir Mastomys natalensis.^214^ LASV is an enveloped virion that contains two single-stranded RNA segments and each segment encodes two proteins.^215^ The NP encapsulates the viral genome segments, which are associated with the transcription of viral mRNAs and replication of genome segments for incorporation into progeny virions.^216,217^ As a surface protein, the glycoprotein complex (GPC) mediates the attachment and entry of virions.^218^ Specifically, after receptor-ligand recognition, LASV virions are internalized via endocytosis. GPC undergoes a conformational shift responds under the regulation of the acidic endosomal environment, causing its binding to the endosomal receptor lysosomal-associated membrane protein 1 (LAMP1).^219,220^ Subsequently, GPC undergoes additional conformational changes that mediate virus-endosomal membrane fusion and enable the release of LASV genome segments into the cytosol. The large (L) protein is an RNA polymerase that is involved in transcription, viral replication, and cap-snatching.^221^. The Zinc-binding (Z) protein serves as the matrix protein and is involved in viral assembly and budding. It is responsible for suppressing both viral and host cell translation and thus negatively regulates viral replication and transcription.^222^

LASV originated in Nigeria, first described in 1969, and subsequently spread to other West African countries.^223,224^ After selection for immune escape and region-associated genetic divergence, mutations accumulate, resulting in the formation of 7 lineages. Three distinct lineages (Lineages I-III) are found in Nigeria and Lineage IV is present in Sierra Leone, Guinea and Liberia.^225,226^ More recently, Lineages V, VI and VII have emerged in Mali, Côte d’Ivoire, Nigeria, Benin and Togo.^227^ According to serological studies, over 500,000 LASV infections and 5,000 deaths occur annually in West Africa.^228^ Imported cases were also documented in nonendemic countries, such as Germany and the Netherlands.^229,230^

Using the functional cellular receptor, alpha-dystroglycan, LASV effectively targeted macrophages, dendritic cells, and endothelial cells as focal points.^231^ LASV antagonizes interferons and subdues the immune system thus preventing their secretion of proinflammatory cytokines. Double-stranded RNAs targeting the exonuclease LASV inhibit IFN responses. This is achieved by assimilation of PAMPs, which helps LASV circumvent the host immune response. Immunosuppression of the host’s innate IFN response is accomplished by halting of interferon regulatory factor-3 (IRF-3) translocation.^232^ Subsequently, LASV can infect most human tissues, resulting in multisystemic malfunctions. The blood vessels are the most afflicted tissues and the LASV replicates in the cells of the reticuloendothelial system, culminating in capillary injury. Bleeding might be observed in the following organs: hepatocytes, intestines, myocardium, lungs and the brain.^233^ Unregulated cytokine expression could be another possible mechanism of LF pathogenesis. In the clinic, failure of multiple organs and shock are accompanied by elevated concentrations of proinflammatory cytokines, IFN- γ, and TNF- α. However, no increase in the levels of either cytokine was detected was observed in another study of lethal LF patients, indicating that IFN- γ and TNF- α concentrations are either increased only in a subset of infected individuals or within a brief duration that could entail continuous testing for detection.^234^ The malfunction of infected DCs culminates in the failure in the secretion of proinflammatory cytokine secretion, the upregulation of costimulatory molecules including CD40, CD80, and CD86, and the abysmal induction of T-cells growth.^235,236^ In another study, human DCs infected with Mopeia virus exhibited stronger induction of CD4^+^ and CD8^+^ T-cell responses than did patients infected with LASV.^237^ The repression of immunological reactions orchestrated by LASV contamination revealed ex vivo is similar in tandem with the outcomes of medical examination, indicating that the lethality result of LF is associated with reduced concentrations or a paucity of interleukin (IL) 8 and IFN inducible protein 10 (IP-10) in circulation.^234^

Lassa fever exhibits a variable clinical course. The incubation period for LF was 6–21 days. LF can progress slowly, and symptoms include general discomfort, fever, sore throat, cough, nausea, vomiting, diarrhea, myalgia, and chest pain.^3,4^ Missed continued/remittent fever, inflammation, and exudation of the eyes and conjunctiva are common symptoms. The vast majority of human infections are mild or asymptomatic, while in some cases multisystem diseases occur. The disease is particularly severe during pregnancy and usually leads to abortion. In severe cases, hypotension, shock, pleural effusion, hemorrhage, epileptic seizures, encephalopathy, facial disease, and neck edema were observed, accompanied by proteinuria and blood concentration. Temporary alopecia and dyskinesia may occur during the recovery period. A small number of patients can develop eighth cranial nerve deafness, and only half of patients can recover some function after 1–3 months. The overall case fatality rate of LF is 1–3%, and the hospitalization case fatality rate is close to 15%. Critical patients usually die within 14 days after disease onset.

### Animal models for Lassa fever

With respect to preclinical animal models of LF, wild-type mice and hamsters were insusceptible to LASV; thus, further approaches such as virus adaptation and immunodeficiency are needed. In contrast, inbred guinea pigs and NHPs are natural susceptibility models for LASV and have been extensively investigated. Additionally, surrogate models based on associated viruses have been developed. In this section, the aforementioned animal models for LF are discussed (Table 4).

**Table 4:** Animal models of Lassa fever

Species	Animal model	Strains	Dose	Route	Lethality	Signs of Disease	Strengths/Weaknesses	References
Rodents	Neonatal mice	–	100 TCID_50_	IP	No	No	Natural host/No clinical signs or lesions	^238^
	CBA mouse	Josiah	10^3^ PFU	IC	70–100%	Weight loss, immobility, scruffy fur, seizures, severe decubitus paralysis	Applied in drug evaluation/Immunodeficient and inapplicable to immune correlates	^239^
	HHD mouse	Ba366	10^6^ PFU	IV	22%	Ruffled fur, lethargy, high level viremia, elevated AST level, severe pneumonitis	Model for pathogenesis study/Immunodeficient and inapplicable to immune correlates	^246^
	STAT1^−/−^ mouse	Josiah	10^3^ PFU	IP	100%	Fever, weight loss, SNHL, viremia	Model for pathogenesis study/Barrier environment needed, MHC-related, age-associated, injection route-dependent pathogenicity, distinguishing characteristics of the human immune system, not applicable to live vaccine evaluation	^241,242,245^
		LF2384	10^5^ PFU		80%			
		LF2450	10^5^ PFU		50%			
	IFNAR^−/−^ mouse	Josiah/AV Ba366/Nig04-10	10^3^ PFU	IV	No	Weight loss, persistent viremia, ruffled fur, hypoactivity		^240–243^
	Chimeric IFNAR^−/−^ B6 mouse	Ba366/Ba298 NNig04-10/Nig-CSF	10^3^ PFU	IP	100%	Weight loss, vascular leakage		^242,244^
	Inbreed Strain13 guinea pig	Josiah	10^4^ TCID_50_	SC IP	>90%	Fever, weight loss, ruffled fur, hunched posture, pneumonia, pneumorrhagia	Obvious clinical symptoms, natural susceptibility/Distinguishing characteristics of the human immune system	^238,251,–253,256,258^
		Z-132		IP	100%			
		Soromba-R		IP	0~57%			
		Pinneo		IP	No	Mild to moderate disease		
		NJ2015	10^4^ PFU	SC	No	Fever, weight loss, conjunctivitis		
	Outbreed Hartly guinea pig	Josiah		SC IP	30~67%	Fever, weight loss, hypothermia neutropenia, lymphopenia, thrombocytopenia	Accessibility, obvious clinical symptom/viral adaptation required and addition mutants acquired	
		GPA-Josiah	10^3^ TCID_50_	IP	100%			
		LF2384	10^4^ PFU	IP	100%			
Nonhuman primates	Squirrel monkey	Bah	10^6.8^ TCID_50_	IM	25%	Anorexia, lassitude, depression, polydipsia	Small size and available NHP/Individuals heterogeneity	^273^
	Marmoset	Josiah	10^6^ PFU	SC	100%	Fever, weight loss, viremia, depression, anorexia	Small size, lower caging and feeding costs/variable clinical course	^271^
	Rhesus monkey	Josiah	10^6.1^ PFU	SC	50–60%	Cough, fever, weight loss, rash, hiccups, lethargy, aphagia, huddled posture, constipation, conjunctivitis, anorexia, decreased water intake, bleeding from the gums and nares	Accurately simulate clinical signs of human/ethical concerns, high cost, inaccessible	^262,263,314^
	Cynomolgus macaque	Josiah	10^4^ PFU	IM	100%	Fever, weight loss, lethargy, anorexia, rashes, facial edema, hunched posture, ruffled fur, piloerection, bleeding from puncture sites, dehydration, epistaxis, acute respiratory syndrome, neurological signs, deafness	Simulate sever cases of human infection/Ethical concerns	^253,264–270,289–292^
		Z-132	10^4^ PFU	IM	100%			
		Soromba-R	10^4^ TCID_50_	IM	66%			
Surrogate models	Inbreed Strain13 guinea pig	Pichinde virus	>3 PFU	SC	100%	Weight loss, ruffled fur, rapid breathing, hypoactivity, decreased appetite	Obvious clinical symptoms, natural susceptibility/difficult to obtain, potential difference between original strains	^277,278^
	Outbreed Hartly guinea pig	Pichinde virus/P18	100 PFU	IP	100%			^274^
	Outbred hamsters	Pirital virus	10^5^ TCID_50_	IP	>50%	Moribund, hemorrhage, pneumonia	Models for studying the mechanism of hepatic injury/Potential differences between Pichinde virus and LASV	^248^
	LVG/Lak hamsters	Pichinde virus	500 PFU	SC	0–100%	Not mentioned	Accessibility/Neonatal or Immunosuppression needed to render lethal infections in adult animals, less susceptible to Pichinde virus compared with MHA/Lak hamsters	^247^
	MHA/Lak inbred hamsters,		35/3.5 × 10^4^/3.5 × 10^6^ PFU	IP	100%		Susceptible to lethal virus infection in both newborns and adult animals/Potential differences between Pichinde virus and LASV	

*TCID*_*50*_ median tissue culture infective dose, *PFU* plaque formation unit, *IP* intraperitoneal, *IC* intracranial, *IV* intravenous, *SC* subcutaneous, *IM* intramuscular, *SNHL* sensorineural hearing loss, *NHP* nonhuman primate, – not applicable

#### Mice

Mastomotic Nasts are the natural hosts of LASV. LASV causes chronic asymptomatic infection in Natal mastomies despite the high virus titers detected in multiple organs.^238^ Due to asymptomatic infection, its use in viral pathogenesis or as a vaccine and therapeutic agent is limited. This model may be useful for basic transmission studies. Similarly, infection with LASV was not lethal for immunocompetent adult mice. Alternatively, LASV can cause lethal disease in suckling mice, SCID mice, IFNAR^−/−^ mice and STAT1^−/−^ mice.^58^

##### CBA mice

Intracranial infection of inbred CBA mice with LASV-Josiah resulted in disease manifestation.^239^ Infected CBA mice presented with scruffy fur, seizures, weight loss, immobility, severe decubitus paralysis, and death. This route of inoculation allowed the onset of signs of disease between 5 and 7 dpi, with 70–100% lethality within 7–12 days.

##### Immunodeficient mice

LASV caused a nonlethal acute infection in 129 Sv IFNAR^−/−^ mice, accompanied by persistent viremia.^240–243^ The infected mice lost weight and exhibited ruffled fur and hypoactivity by 11 dpi.^241–243^ The viral load was detected in multiple organs.^240^ Similar pathological changes and disease signs were found in these animals upon infection with a variety of LASV strains, including Josiah, AV, BA366, and Nig04–10.^240,241^ To establish a lethal model of LASV, chimeric IFNAR^−/−^B6 mice were established by irradiating and transplanting bone marrow progenitor cells from wild-type C57BL/6 mice into IFNAR^−/−^ mice. These IFNAR^−/−B6^ chimeric mice succumbed to LASV infection within 10 days^164,166^ This model recapitulates the abnormal hematological indices observed in LF patients, including an elevated AST/ALT ratio, FAS and FAS-L.^242,244^ Depletion of CD8^+^ T cells in IFNAR^−/−B6^ mice significantly increased the postinfection survival rate to 87.5% and reduced the FAS and FAS-L concentrations, as well as vascular leakage in the liver and lung despite persistent viremia, which indicated that T cells play a role in the pathogenesis of LF.^242^ In contrast, IFNαβ/γ^R−/−^ 129 Sv mice did not develop clinical signs of disease upon LASV infection apart from minor and transient weight loss.^243^

STAT1^−/−^129Sv mice are highly susceptible to LASV, which progresses to lethal disease accompanied by typical clinical manifestations, such as sensorineural hearing loss (SNHL).^241,245^ Mice intraperitoneally infected with 10^4^ PFUs of LASV-Josiah lost weight and died at 7 dpi.^241^ This model recapitulates the clinical outcome of patients infected with LASV-LF2384 and LASV-LF2350.^245^ Clinical signs of disease, disseminated viruses to multiple organs, prolonged viremia, and abnormal hematological indices and histologic results were observed after lethal infection with LASV-LF2384 in STAT1^−/−^129Sv mice. Notably, compared to that in IFNαβ/γ^R−/−^ mice, IFN signaling was not completely disrupted in STAT1^−/−^ mice. Partial knockout of the STAT1 gene results in the expression of a truncated form of STAT1 that can still mediate minimal T-cell responses. Since T cells contribute to the pathogenesis of LF, differences in IFN signaling may explain why STAT1^−/−^ mice are more susceptible to LASV than are IFN αβ/γ^R−/−^ mice.^242^ Notably, the STAT1^−/−^ model is the only available small animal model of SNHL. The clinical isolates LASV/LF2384 and LASV/LF2350 from the 2012 Sierra Leone outbreak caused deafness in survivors.^245^ A total of 10^5^ PFU of viral infection resulted in permanent hearing loss in all survivors. With the lower dose of LASV infection (10^4^ PFU), hearing loss was observed in 20% of the survivors.

##### Humanized HHD mice

C57BL/6 mice expressing human/mouse chimeric HLA-A2.1 instead of the normal MHC class I gene product (humanized HHD mice) were established as a model for LASV infection.^246^ HHD mice were susceptible to LASV-BA366 infection, for which the fatality rate was approximately 22%. LASV infection leads to the rapid onset of disease, which can include ruffled fur, lethargy, and elevated serum AST concentrations. High viral titers were detected in the liver, lung, and spleen, whereas lower titers were detected in the kidney. Histological examination revealed severe pneumonia with signs of pleural effusion, interlobular septal thickening, collapse of the alveolar space, and infiltration of monocytes and macrophages. The liver contains monocytes and macrophages with altered cellular distribution, orientation, and shape. The spleen also exhibited destruction of both the white and red pulp areas. Depletion of CD4^+^ T cells, CD8^+^ T cells, or both, resulted in significant differences in disease severity in this model. After infection, the serum AST concentrations in HHD mice lacking CD4^+^ and CD8^+^ T cells remained normal, whereas the AST concentrations increased in HHD mice. In addition, depletion of CD4^+^ T cells or CD8^+^ T cells resulted in a partial increase in the AST concentration. In all the groups, similar cases of high-titer viraemia developed, indicating that T cells did not have a substantial effect on viraemia. C57BL/6 mice lacking only CD4^+^ T cells were able to clear the viral infection, whereas C57BL/6 mice lacking only CD8^+^ cells exhibited persistent viremia. These findings were confirmed in MHC-I^−/−^ mice, which lack CD8^+^ T cells; despite high viremia, no clinical manifestations were observed post infection. Neither CD4^+^ nor CD8^+^ T-cell-depleted mice exhibited any significant histological changes in the lungs or spleen after infection or signs of disease, which underscores the role of T cells in LASV pathogenesis.

Overall, immunodeficient mice are susceptible to LASV infection but generally develop mild disease and survive infection. Chimeric IFNAR^−/−B6^ mice are susceptible to lethal LASV infection but require irradiation and transplantation of bone marrow progenitor cells from wild-type C57BL/6 mice. The findings from STAT1^−/−^ mice recapitulated the pathogenic potency of different LASV isolates and some LF disease signs, including hearing loss.

#### Syrian hamsters

Syrian hamsters were insusceptible to LASV. Pichinde virus, another member of the Arenaviridae genus, was used to establish an infection model in hamsters. Pichinde virus infection caused LF-like disease in Syrian hamsters, and the lethality of the virus varied depending on the strain. Newborn LVG/Lak outbred hamsters, together with newborn and adult MHA/Lak inbred hamsters, were up to 100% lethal to Pichinde virus. Both models produced antibodies against Pichinde virus. The main target organs were the spleen, liver, and kidney.^247^ Pirital virus, a nonhuman pathogenic New World mammarenavirus isolated from western Venezuela, also causes LF-like disease in Syrian hamsters.^248^ Infected hamsters developed severe disease and died at approximately 7 dpi.^248^ Histopathology revealed interstitial pneumonia, multifocal hepatic necrosis, and reduced and necrotic splenic lymphoid tissue. A proportion of the hamsters exhibited oral hemorrhage and coagulopathy.^248^ Although these surrogate viruses effectively infect hamsters, the genetic differences between LASVs should be fully addressed.

#### Guinea pigs

The guinea pig model has been extensively used to study the pathogenesis of LF and to evaluate potential treatments and vaccine candidates for this disease.^249^ The inbred Strain 13 guinea pigs are naturally susceptible model of LASV, while outbred Hartley guinea pigs require viral adaptation in vivo to achieve uniform lethality. The lethality of different strains in guinea pigs infected with LASV varied. Clinical signs in Strain 13 guinea pigs infected with LASV-Josiah include fever, anorexia, weight loss, humping, fur crumpling, and altered mental status.^250,251^ SC inoculation of two or more PFU of LASV-Josiah in Strain 13 guinea pigs resulted in death, while Hartley guinea pigs were relatively resistant and had a mortality rate of 30–67% following infection. More rapid viraemia and higher viral titers were observed in strain 13 guinea pigs than in Hartley guinea pigs. Virus titration in guinea pigs revealed that the highest titers were extracted from the lung and spleen, followed by the pancreas, lymph nodes, adrenal glands, kidneys, salivary gland, liver, and heart. Histopathological findings indicated mild to moderate interstitial pneumonia, acute necrotizing nephritis, mild myocarditis, and mild hepatitis. Conjunctivitis and conjunctival edema with ocular discharge were also observed.^172,174,175^ Infected animals did not generate neutralizing antibodies.^250^ The concentrations of LASV-Soromba-Rand and Z-132 were also assessed in Strain 13 guinea pigs. Following IP challenge with 10^4^ TCID_50_ of the virus, all the guinea pigs died, and the mortality rate in guinea pigs infected with Soromba-R was 57%. No obvious disease features were apparent in the surviving guinea pigs after Soromba-R infection. Histopathological changes were observed in the lungs, liver, and spleen, and these changes were identical to those observed in LASV-Josiah infection.^172,176^ LASV-NJ2015 and Pinneo were nonlethal in Strain 13 guinea pigs. Infected animals exhibited mild to moderate disease.^252^ Animals infected with LASV-NML-57 exhibited rapid increases in temperature and weight loss, while those infected with the isolate NML-33 had a high mortality rate of up to 90%.^253^ LF survivors exhibited sequelae, including polyserositis, visual distortion, vertigo, epididymitis, back pain, and partial or permanent hearing loss. The persistence of the virus in the smooth muscle cells of the tunica media of arteries in surviving guinea pigs suggested that guinea pigs may be used as a model for studying chronic LF infection in humans.^254^ Although no viral antigens were detected in the ears of the surviving guinea pigs, perivascular mononuclear cell inflammation was present in the ears and pars compacta, which led us to hypothesize that the inflammatory response was the primary cause of hearing loss.^255^ The similarity of the symptoms of LASV infection in Strain 13 guinea pigs to those in humans and the persistence of the virus in surviving guinea pigs further support the rational application of the guinea pig model.

To obtain a uniform lethal model, a guinea pig-adapted LASV was established by successive passages of LASV-Josiah.^238,256,257^ Guinea pigs intraperitoneally injected with 10^3^ PFU of guinea pig-adapted LASV (GPA-LASV) exhibited significant weight loss of 8–20%, up to the criteria for euthanasia. On average, death occurred at 15 dpi.^258^ Viral titers were detected in serum, spleen, liver, and lung.^252^ The histopathological manifestations of infection with guinea pig adapted LASV (GPA-LASV) were hepatocellular degeneration, lymphohistiocytic hepatitis, sinus histiocytosis, and interstitial pneumonia. Clinical isolates of LASV that cause lethal infection in Hartley guinea pigs have also been reported.^259^ A dose of 10^2^ PFU of LASV LF2384 was 100% lethal in guinea pigs. As in IF patients, guinea pigs presented thrombocytopenia, neutropenia, and lymphopenia. Unfortunately, nonspecific symptoms, such as respiratory and cardiac involvement, which differentiate these patients from human patients were noted.^252,260,261^

#### Nonhuman primates

##### Rhesus macaques

Rhesus macaques developed severe disease and prolonged viremia after LASV-Josiah infection.^262^ Symptoms of disease, including severe petechial rash, hiccups, lethargy, aphagia, huddled posture, constipation, conjunctivitis, anorexia, weight loss, decreased water intake/dehydration, facial and periorbital edema, bleeding from the gums and nares, cough, and a slight fever, appeared at 7 dpi.^262,263^ Fever persisted until death, or sudden hypothermia occurred before death. SC inoculation with 10^6.1^ PFU of LASV Josiah was lethal in 60% of the animals. Serologic analysis indicated that the serum concentration was altered at 10–12 dpi but was not correlated with viral clearance or animal recovery. Elevated AST, ALT, and blood urea nitrogen (BUN) levels with transient and moderate leukopenia were also noted. Hematocrit, hemoglobin, fibronectin, and red blood cell counts decreased. Viremia typically appeared at 4–5 dpi, with titers greater than 10^4^ PFU/mL in lethally infected monkeys, which was significantly greater than that in survivors. Viral loads were detected in the adrenal glands, liver, lung, pancreas, brain, bone marrow, kidney, lymph nodes, spleen, muscle, heart, thymus, testis, salivary gland, urine, CSF, and intestines. The highest titers were in the liver, spleen, and adrenal glands.

Gross pathological analysis indicated scattered petechial and visceral hemorrhage with the presence of mild to moderate pleural effusions. Liver and adrenal gland tissues presented necrosis, with regeneration of hepatocytes and slight infiltration of inflammatory cells. Interstitial pneumonia with edema, thickened alveolar septae, and pulmonary arteritis were present in the lung. Spleen samples indicated lymphocytopenia and the presence of viral antigens in the red pulp. Infected primates also develop mild to moderate interstitial and perivascular myocarditis and pericardial edema. Severe meningoencephalitis with significant perivascular cuffing was noted. Infiltration of erythrocytes and macrophages was noted in the small intestine. Lesions and a multifocal cortical interstitial mononuclear infiltrate were noted in the kidney. Seventy-eight percent of infected primates develop lesions in the central nervous system (CNS) with mild lymphocytic cuffing of the vessels of the brain, spinal cord, and meninges. Twenty percent of the primates suffered lymphocytic infiltration of the spiral ganglia, and mild chorioretinitis was also noted. The arterial lesions, vasculitis, meningoencephalomyelitis and skeletal myositis observed in this monkey model were rarely, if at all, noted in human LF patients. Overall, rhesus macaques are thought to be the most accurate model of human LF.

##### Cynomolgus macaques

Cynomolgus macaques closely simulate severe cases of LF in humans.^264^ Disease severity was largely dependent on the viral strain. The LASV lineage IV (Josiah/Z-132) and the lineage VII strains cause uniform lethality in cynomolgus macaques, whereas infection with lineage V strains (Soromba-R/AV) induces mild to moderate manifestations without lethal outcomes.^265–268^ Compared to those infected with lineage VII in macaques, lineage II-infected animals presented longer survival times and lower mortality rates.^267^ However, lineage III strains isolated from patients during the 2018 Nigerian outbreak showed differences in pathogenicity in cynomolgus macaques.^253^ Common symptoms in LASV-infected cynomolgus macaques include fever, epistaxis, weight loss, tachycardia, hypotension, and tachypnea, which are similar to those observed in severe human diseases.^269^ Notably, in infected macaques, LASV causes neurological diseases such as meningoencephalitis and neuronal necrosis. During necropsy, axillary and inguinal lymphadenopathy and congestion are observed in infected animals.^266^ Biochemical analysis revealed that infection with LASV increased AST, ALT, ALP and CRP in macaques.^265–268,270^ The inflammatory response and extent of viral replication are associated with the severity of the disease. Post infection, increased levels of cytokines and chemokines, such as IL-1β, IL-6, TNFα, MCP1, and MIP1β, were observed in a macaque model.^265–268,270^

##### Marmosets

The common marmoset (Callithrix jacchus) is a small anthropoid primate that generally weighs between 320 and 450 g and has been developed as another small NHP model of LF.^271^ Experimental infection of common marmosets with a low dose (1 × 10^3^ PFU) or high dose (1 × 10^6^ PFU) of LASV-Josiah resulted in systemic viral distribution, accompanied by a high viral RNA load in multiple tissues. Elevated liver enzymes, decreased plasma ALB levels, weight loss, and morbidity between 15 and 20 dpi were observed. Additionally, increases in AST, ALT, and AKP as well as a gradual decrease in platelet counts were found in both the low-dose and high-dose infection groups. An enlarged liver accompanied by mild inflammation and multifocal hepatic necrosis, an enlarged spleen with mild to moderate lymphoid depletion, and lung abnormalities characterized by hemorrhage in most lobes were confirmed in infected animals.

##### Squirrel monkeys

Virological and pathological studies of LFV have been performed on squirrel monkeys (Saimiri scirreus).^239,272^ The small size and availability of marmosets make them attractive alternatives to other NHPs. After intramuscular inoculation with 10^6.8^ TCID_50_ LASV-Bah, the monkeys developed common clinical symptoms, including anorexia, lassitude, depression, and polydipsia. Early viral lymphoreticulotropism, nephrotropism, hepatotropism, and viraemia were noted. At the endpoint, viral titers in target organs were associated with necrotic changes, such as splenic lymphoid necrosis, renal tubular necrosis, myocarditis, arteritis, and hepatocytic regeneration. In particular, the pathological findings in the liver and spleen were similar to those in humans.^272^ In convalescent monkeys, viral titers in multiple organs diminished slowly, and viraemia persisted for 28 days without antibody conversion. Renal and splenic regeneration occurred, and a new lesion, choriomeningitis, was present. However, compared to that in other nonhuman primates, the pathogenicity of LASV in squirrel monkeys was relatively low, with a mortality rate of 25%.^273^ Clinical symptoms showed heterogeneity among individuals, which hindered further application of the model.

#### Surrogate models

Due to the limitations of biosafety level 4 laboratories (BSL-4), surrogate models of LASV were established, which would be helpful for basic studies and the evaluation of safety at early stages of preclinical development on a case-by-case basis. Infection of guinea pigs and hamsters with alternate viruses can cause LF-like disease; therefore, surrogate models of LF have been established. Pichindé virus (PICV), a new world mammarenavirus isolated from cricetin rodents in Colombia, South America, has been applied in the establishment of models.^272^ Consecutive passages of PICV more than 4 times in guinea pigs produced a model with 100% lethality.^274^ Infected strain 13 guinea pigs showed reduced activity, fur ruffling, anorexia, weight loss, lethargy, and rapid breathing; viremia emerged at 2 dpi and increased steadily at 16 dpi until death.^275,276^ Histopathology revealed lesions in the liver, spleen, pancreas, lungs, and gastrointestinal tract, with scattered areas of necrosis observed in the lymphoid tissue and bone marrow.^276^ In contrast, Hartley guinea pigs infected with 3 × 10^2^ PFU of PICV CoAn 4763 exhibited a lethality rate of approximately 43%.^274^ Subsequently, 18 passages of PICV CoAn 4763 were generated in strain 13 guinea pigs, and infection with 10^2^ PFU of PICV P18 resulted in uniform lethality in Hartley guinea pigs. The virus was detected in multiple organs, with the highest titers in the adrenal glands, lungs, stomach, and liver, and lower titers in the brain.^277,278^ Newborn rats have also been investigated as a model for LF by intracerebral inoculation with lymphocytic choriomeningitis virus (LCMV). Infected animals showed acoustic startle reflexes. Infected animals had higher elicitation and inhibition thresholds and showed recruitment at intense stimulus levels. Histopathology revealed both cochlear and retinal degeneration. These results highlight the potential of this technique for treating severe polysensory neuropathy in rats.^279^ The LCMV WE strain has also been used to establish a surrogate model in rhesus macaques. Despite the lower biosafety requirement of LCMV, the extent to which this fungus mimics LASV infection is largely uncertain and warrants further investigation.^280^

### Medical countermeasures for Lassa fever

No vaccines or countermeasures have been approved for use in LF. Several vaccine candidates have achieved progress in animal models and moved into clinical trials. Ribavirin is the only off-label treatment.^281^ More recently, progress has moved towards the development of immunotherapeutic and small-molecule drugs (Table 5).

**Table 5:** Representative medical countermeasures for treating Lassa fever

Classification	Name	Platform/Strategy	Stage	Efficacy/Benefic	References
Vaccines	LASSARAB	Expressing LASV-G using attenuated RABV vector	Preclinical	Protect guinea pigs and mice; Fc mediated protection	^282,283^
	Live attenuated	Replacing IGR of LASV S segment or breaking codon bias	Preclinical	Protects guinea pigs	^284,285^
	ML29	Recombinant virus of LASV and Moopeia virus	Clinical	Sterile immunity and protects guinea pigs and NHPs	^286–291^
	VSVΔG/LASV GPC	Expressing LASV-GPC based on VSV	Preclinical	Robust and long-lasting immune response and 100% protection in NHPs	^252,292,293^
	YF17D/LASV-GPC	Expressing LASV GPC based on flavivirus YF17D	Preclinical	Protect guinea pigs but not NHPs	^294^
	MeV-NPmut/GPC and MeV-Z/GPC	Expressing LASV GPC + NP or GPC + Z based on MeV vector	Clinical	Protect NHPs from lethal challenge and nearly sterile immunity	^296–298^
	Vaccinia virus-NP /GPC	Expressing LASV GPC(GP1/GP2) or NP based on Vaccinia virus vector	Preclinical	Distinct results in rodents and NHPs	^299–301^
	RNA replicon	RNA replicon expressing GPC or NP of LASV	Preclinical	Protect guinea pigs	^302,303^
	INO-4500	DNA vaccine encoding LASV GPC	Clinical	Protect guinea pigs and NHPs	^305,306^
Antibodies	Arevirumab-3	Human antibody cocktail contains 8.9F, 12.1F, and 37.2D	Clinical	Protective in guinea pigs and NHPs	^53,258,320^
Small molecular drugs	Fapiravir	RdRp inhibitor	Preclinical	Improved situation in mice	^317^
	Ribavirin	Broad spectrum antiviral drug	Preclinical	Recommended to use in the early stage of the disease	^313–315^

*LASV* Lassa virus, *RABV* rabies virus, *IGR* gene interval, *GPC* glycoprotein precursor, *VSV* vesicular stomatitis virus, *N* nucleocapsid protein, *Z* zinc- binding protein, *RdRp* RNA dependent RNA polymerase, *NHPs* nonhuman primates

#### Preventative vaccines for Lassa fever

Inactivated vaccines for LASV have been developed based on rabies virus vectors. These vaccines were designed by expressing LASV GPC using the live attenuated RABV vector BNSP333. Compared to the LASSARAB live vaccine, inactivated LASSARAB induced robust and persistent humoral immune responses in mice and guinea pigs. Inactivated LASSARAB could protect guinea pigs and mice, and antibody-dependent cell-mediated cytotoxicity (ADCC) and antibody-dependent cell-mediated phagocytosis (ADCP) are the main mechanisms of protection.^282,283^

Live attenuated rLASV (IGR/S-S) was obtained by replacing the gene interval (IGR) of the LASV S segment. Compared to those of wild-type strains, the growth of strain on the cell decreased, the pathogenicity of the strain on the guinea pig decreased, and the strain could completely protect the guinea pig from lethal attack by LASV.^284^ After immunization, the serum LASV IgG titer increased, while the neutralizing antibody titer decreased, which could only be detected recently. RLASV (IGR/S-S) has been passed through cells for 15 generations and is genetically stable. Moreover, the team also attempted to modify the attenuated vaccine rLASV-GPC/CD by disrupting codon bias, which has similar protective effects.^285^

The recombinant MOPV/LASV(ML29) vaccine is a recombinant virus of LASV and Moopeia viruses that retains the nonpathogenic L segment of MOPV and the S segment of the LASV Josiah strain.^286,287^ Sterile immunity was achieved in guinea pigs and NHPs after immunization, and the protection rate was 100%.^288^ In addition, the ML29 vaccine still conferred 80% protection when it was administered 2 days after exposure.^289,290^ Using reverse genetics approaches to knock down the exon function of MOPV, the transmission and replication ability of MOPV were attenuated. The highly attenuated strain MOPEVAC_LAS_ included multiple mutations in MOPV NPs and the exchange of the GPC ORF sequences of LASV and MOPV greatly improved its immunogenicity. Single-dose injection of MOPEVAC_LAS_ conferred protection in nonhuman primates.^291^

Several viral vectored LASV vaccines have been investigated and shown to have potential benefits. VSV-based vaccines expressing LASV-GPC was constructed, namely VSVΔG/LASV GPC, a single intramuscular dose vaccination of which induced robust and long lasting cellular and humoral immunity in NHPs and conferred 100% protection.^292^ Cross-protection between different viral strains in guinea pigs and macaques has also been reported.^252,293^ A flavivirus vectored LASV vaccine was prepared by inserting LASV GPC between the E and NS1 genes of flavivirus YF17D, termed YF17D/LASV-GPC. YF17D/LASV-GPC was replication-competent and showed significantly reduced toxicity.^294^ It induced an immune response and protected 80% of guinea pigs against the lethal challenge of LASV. However, the genetic stability of the virus is not ideal, and the expression of GPC decreased after 5 generations. Recombinant viruses expressing GP1 or GP2 were constructed. The combination of the two recombinant viruses protected 83% of the guinea pigs from lethal challenge with LASV, but did not result in sterile immunity. However, these results in guinea pigs could not be replicated in NHPs.^295,296^ Two measles virus (MV) vector LF vaccines expressing LASV GPC + NP or GPC + Z were constructed. Both MeV-NPmut/GPC and MeV-Z/GPC were immunogenic in NHPs.^296^ Single dose vaccination with MV-LASV-NP + GPC can protect macaques from lethal challenge and nearly achieve sterile immunity.^297,298^ The recombinant vaccinia virus encoding LASV NP conferred 100% protection in Hartley guinea pigs from homologous challenge.^299^ In guinea pigs, the protection rates of recombinant vaccines expressing LASV NP or GPC were 94 and 79%, respectively, while the protection rates of recombinant vaccines expressing both GP and NP were lower. Interestingly, in NHPs, survival rates reached more than 90% when the GP1, GP2 and NP proteins were expressed simultaneously based on vaccinia virus, but none survived when these immunogens were expressed alone.^300^ These results indicate the different mechanisms involved in triggering the immune response and the mechanism of protection against LASV in rodents and NHPs.

Salvato et al. constructed a novel VLP vaccine by expressing LASV GPC and Z protein based on the MVA vector, termed GEO-LM01.^301^ A Venezuelan equine encephalitis virus (VEEV) RNA replicon expressing GPC or the NP of LASV was also developed, which completely protected inbred guinea pigs from lethal infection by homologous LASV.^302^ Two cistron RNA replicons that simultaneously express two GPCs from different lineages of LASV were also developed and can mediate a cross-reactive immune response in mice.^303^ However, three doses of RNA replicons are needed to achieve protection. Two doses of Ad5-LASV-NPs and Ad5-LASV-GPC could protect guinea pigs from lethal LASV challenge, and the activation of humoral immunity could be detected.^304^

An optimized DNA vaccine encoding LASV GPC can fully protect guinea pigs and NHPs from LF. Cellular immunity is the main protective mechanism.^305,306^ Subsequent studies have shown that after secondary immunization, 100% of NHPs produce neutralizing antibodies, and a large proportion of them produce LASV GPC-specific T-cell responses.^307^ A Phase I clinical trial of INO-4500 (NCT 03805984) is ongoing. Moreover, a broad-spectrum peptide vaccine against seven pathogenic arenaviruses that expressed conserved epitopes from LCM, Lassa, Guanarito, Junin, Machupo, Sabia, and Whitewater Arroyo viruses was reported.^308,309^

#### Therapies for Lassa fever

Convalescent plasma therapy (CPT) has been applied in the treatment of LF. In a case report, when plasma was injected in the early stages of the disease, a beneficial effect was observed.^310^ When CSP was given shortly after exposure to LASV, the cynomolgus monkeys and guinea pigs were protected.^311^ However, the protective efficacy of the passive transfer of CSP in human clinical trials is controversial.^312^

Ribavirin has been shown to reduce the mortality of high-risk LF patients with elevated liver enzymes from 55 to 5% on admission, but the drug is relatively expensive, and the premise is to treat LF within a week of disease duration.^313,314^ Overall, ribavirin is commonly used clinically for LF in the early stage of the disease, but it is not recommended for postexposure prophylaxis.^315,316^ Currently, data on the clinical efficacy of this drug are limited, and the efficacy of ribavirin is not obvious in patients with mild disease. When favipiravir (T-705) was combined with ribavirin, health of LASV infected mice was improved.^317^

LHF-535 was discovered after high throughput screening and structural optimization of benzimidazole derivatives. Ikenna G. Madu et al. determined that LHF-535 has subnanomolar potency against viral envelope glycoproteins in all Lassa virus lineages, but the sensitivity of glycoproteins of strains from lineage I is 100 times lower than that of other viruses.^318^ This decrease is mediated by the unique amino acid substitution V434I in the transmembrane region of the GP2 subunit of the envelope glycoprotein.

Several mouse monoclonal antibodies against LASV and Mopeia viruses were isolated by the hybridoma technique, most of which reacted with NP, GP2 or GP1. Glycoprotein-specific monoclonal antibodies have shown limited neutralization ability.^319^ Human mAbs against LASV glycoproteins were also isolated from B cells of LF survivors. Of these mAbs, half were able to bind the GP2 fusion subunit, a quarter recognized the GP1 receptor binding subunit, and the remaining quarter specifically recognized the protein complex. Fifteen monoclonal antibodies were able to neutralize the LASV pseudovirus.^53^ In guinea pigs, humAbs, 25.6A, 2.9D, 8.9F, 12.1F, and 37.7H conferred 100% protection.^258^ In cynomolgus monkey, 37.2D, 12.1F, 8.9F, 19.7E and 37.7H protected all animals. However, the virus was still detectable at 21 dpi in the 8.9F treatment group, which was attributed to the formulation of an escape strain of LASV, 8.9F(e). LASV, 8.9F(e) escaped the neutralizing effect of 8.9F by affecting the four-level epitope required for 8.9F binding, but was completely neutralized in vitro by a mixture of 8.9F, 12.1F, and 37.2D, as this cocktail binds to GPs from four separate branches of LASV. Treatment with this cocktail at 8 days post LASV treatment protected 100% of the rhesus monkeys. This antibody cocktail, called Arevirumab-3, is under clinical investigation.^320^

## Beta-coronavirus diseases

### Etiology, epidemiology, and pathogenesis of beta-coronavirus diseases

Pathogenic coronaviruses are associated with respiratory and intestinal infections in animals and humans. Representatively, SARS-CoV, MERS-CoV and SARS-CoV-2 are highly pathogenic to humans and are responsible for pneumonia and other respiratory symptoms.^321–323^ SARS-CoV, MERS-CoV and SARS-CoV-2 belong to the beta coronavirus (Beta-CoV) genus, coronavirus subfamily that belongs to the order Nidovirales.^324^ These viruses are enveloped RNA viruses, with a diameter of approximately 100–160 nm and the largest genome among RNA viruses.^325^ There are three surface proteins on the lipid membrane that encapsulate virus particles, namely the spike protein (S), envelope protein (E), and membrane protein (M). The S protein is responsible for receptor binding and cell lysis, and mediates viral invasion.^326^ SARS-CoV and SARS-CoV-2 primarily infect ciliated bronchial epithelial cells and type II lung cells via angiotensin converting enzyme (ACE2) as a functional receptor, while MERS-CoV infects undifferentiated bronchial epithelial cells and type II lung cells via dipeptidyl peptidase 4 (DPP4, also known as CD26) as a receptor.^327–329^ After receptor-ligand recognition of SARS-CoV/SARS-CoV-2 and host cells, transmembrane serine protease 2 (TMPRSS2) on the cell surface cleaves ACE2 and activates the S protein, thereby promoting virus entry. The viral genome utilizes host ribosomes to directly translate two types of polymeric proteins, pp1a and pp1ab, which are cleaved into 16 nonstructural proteins by two proteases, papain PLpro and the main protease Mpro, which are assembled into transcriptional replication complexes responsible for transcriptional replication of the viral subgenome and genome. These components will continue to assemble mature viruses in the endoplasmic reticulum and Golgi apparatus and be released from the cell membrane to initiate the next round of infection. In this process, the S protein, PLpro, Mpro, and viral RNA-dependent RNA polymerase (RdRP) are all key antiviral drug targets. In addition to the direct pathogenic effect of the virus, an excessive immune response is another pathogenic issue of SARS-CoV-2.^330^ The affinity between the RBD and ACE2 in the S1 subunit of the S protein of SARS-CoV-2 is 10–20 times greater than that of SARS-CoV, which could explain the stronger transmission ability of SARS-CoV-2.^331^ In contrast, MERS-CoV mainly infects the lower respiratory tract, promotes the replication and production of viruses in macrophages and dendritic cells, induces the production of proinflammatory cytokines, targets T lymphocytes, and leads to their apoptosis.^329^

There are multiple gaps in understanding the epidemiology, pathogenesis, and countermeasures of SARS-CoV and MERS-CoV. SARS-CoV and MERS-CoV were transmitted directly to humans from commercial civets and dromedary camels, and both viruses originated from bats.^325^ There are currently no approved or available prophylactic or therapeutic approaches, and potential vaccines are at an early preclinical stage. Consequently, treatment remains largely supportive in clinical. In contrast, the COVID-19 pandemic has accelerated the advances in animal models, vaccines, antibodies, and small molecule drugs, and a large number of candidate products have been approved or entered Phase III/IV clinical trials. However, the origin of SARS-CoV-2 has not yet been clearly documented. Due to the wide range of techniques and abundant clinical trials involved, we focused on sophisticated or advanced products for the prevention and clinical treatment of COVID-19. These attempts can also provide a reference for the prevention and control of SARS-CoV and MERS CoV.

### Animal models for beta-coronavirus diseases

To obtain in-depth insights and better prepare for medical responses, animal models of beta-coronavirus diseases, including naturally susceptible and artificially modified animal models, were generated. In this section, animal models for beta-coronavirus diseases are discussed in detail (Table 6).

**Table 6:** Animal models of beta-coronaviruses

Species	Animal model	Pathogens	Dose	Route	Lethality	Signs of disease	Characteristics (Strengths/Weaknesses)	References
Mouse	HFH4-hACE2-mice	SARS-CoV-2	3 × 10^4^ TCID_50_	IN	0	No	Applicable to pathogenesis study/No obvious signs of disease	^332^
	K18-hACE2/hDPP4 mice	SARS-CoV-2	10^5^ PFU	IN	50 ~ 100%	Encephalitis, pneumonia, anosmia, respiratory distress	Mimic severe disease, a model for anosmia/Nonspecific brain infection	^335–337^
		SARS-CoV	2.28 × 10^5^ PFU		100%			^333^
		MERS-CoV	10^5^ PFU		100%			^334^
	hACE2 knock-in mcie	SARS-CoV-2	4 × 10^5^ PFU	IN	0	Interstitial pneumonia	hACE2 and viral load in line with humans, intragastric infection/Brain infection	^338^
	hACE2 knock-in mice	SARS-CoV-2	4 × 10^5^ PFU	IT	–	Pneumonia	Model for ARDS, typical pathological changes in lung/-	^339^
	hCD26/DPP4 mice	MERS-CoV	10^6^ TCID_50_	IN	100%	Weight loss, pneumonia	Low dose lethal infection/Brain infection	^340–342,897^
	Humanized mouse	MERS-CoV	2 × 10^5^ PFU	IN	0	No	Humanization of DPP4 using VelociGene technology/No obvious signs of disease	^343^
	Humanized mouse	MERS-CoV	10^5^ PFU	IN	0	/	Expanded tropism for pathogens, human innate and adaptive immune system	^821^
	CRISPR–Cas9/mice adapted virus	MERS-CoV	5 × 10^6^ PFU	IN	50%	Weight loss, pneumonia	A model for ARDS, signs indicative of end-stage lung disease/Additional mutants introduced by virus adaptation	^344,345^
	Ad5-hACE2/hDPP4 transduce	SARS-CoV-2	10^5^ FFU	IN	0	Pneumonia	Rapid acquisition of susceptible animal models/mimic limited physiological features of infection, inaccurate receptor expression	^346,347,349^
		MERS-CoV	10^5^ PFU	IN	0			^348^
	Adeno-associated virus transduce	SARS-CoV-2	1–3 × 10^6^ PFU	IN	0	Weight loss, lung fibrosis	Recapitulated human immune response and chronic COVID-19 infection/high unit cost, infection limited to hematopoietic cells	^351^
	Mice adapted model	SARS-CoV	10^4.4^ TCID_50_	IN	100%	Viremia, overwhelming infection	High titer viral replication in lungs, dissemination of virus to extrapulmonary sites/Differences from the original strain	^353^
		MERS-CoV	10^6^ PFU	IN	0 ~ 100%	Weight loss		^354^
		MASCp6	1.6 × 10^4^ PFU	IN	0	Pneumonia, inflammatory responses Weight loss	Enable sensitive infection/None lethal model, differences from the original strain	^355^
		HRB26M	10^4.4^ PFU	IN	0			^356^
		MA10	10^5^ PFU	IN	10 ~ 90%		ALI, ARDS; aged-related disease severity/Differences from the original strain	^357^
		BMA8/C57MA14	10^5^ TCID_50_	IN	0 ~ 100%		Genetic and age-dependent severity/Differences from the original strain	^358^
Hamster	Wild type	SARS-CoV	10^3^ TCID_50_	IN	0	No	Rapid acquisition of susceptible animal models/Immunosuppression needed to sensitive infection, not applicable to extensive usage	^367,368^
	Immunosuppressed	SARS-CoV	10^3^ TCID_50_	IN	100%	Weight loss		^369^
	Wild type	SARS-CoV-2	8 × 10^4^ TCID_50_	IN	0	Rapid breathing, weight loss	Transmission between hamsters, mild to moderate infection, aged-dependent severity/None-lethal model	^370–373,898,899^
	Chinese hamster		10^5^ PFU	IN	0	Pneumonia and bronchitis	Prolonged period of weight lose/Milder bronchitis and pneumonia and shorter duration	^384^
	Roborovski hamster		10^5^ PFU	IN	0		Rapid and fatal course, systemic infection	^382,383^
	STAT2^−/−^ hamsters		2 × 10^5^ TCID_50_	IN	0		Model for pathogenesis study/Not applicable to study immune correlates	^386^
	Immunosuppressed		10^4^ PFU	IN	0 ~ 60%	Chronic infection	Low doses of virus and more severe and prolonged disease/Not applicable to extensive usage	^387^
	hACE2 transgene hamster	SARS-CoV-2	100/1000 PFU	IN	66.7–100%	Extensive respiratory and CNS infection	Severe and lethal infection models/ectopic expression of the hACE2	^381^
Ferret	Wild type	SARS-CoV	10^6^ TCID_50_	IT	0	Fever, lethargic, sneezing, conjunctivitis, pneumonia	Models for contact and aerosol infection/None-lethal model	^389–392^
	Wild type	SARS-CoV-2	10^5.5^ TCID_50_	IN	0	Common symptoms, loss of taste or smell, rash, red or irritated eyes, difficulty in breathing, chest pain	Acute bronchiolitis, transmit by direct or indirect contact; dose-dependent infection/Transient virus replication, no obvious clinical symptoms in young animals	^398,900^
Mink	Wild type	SARS-CoV-2	5 × 10^6^ PFU	IN	0	Severe pneumonia	Animal model for severe disease, related variants emerged, mild to moderate infection by IN infection route and severe infection by IT route/-	^406^
		SARS-CoV-2	4 × 10^5^ PFU	IN/IT		Lethargy, diarrhea, nasal and sneezing		^407^
NHPs	Rhesus macaques	SARS-CoV-2	4.75 × 10^6^ PFU	IN	0	Respiratory abnormalities	Mild clinical disease, viral replication and pathology/Lack of typical clinical symptoms and no ARDS observed	^411,413^
		MERS-CoV	7 × 10^6^ TCID_50_	IT + OR + IN + OC	0	Transient infection, pneumonia	Model for the lower respiratory tract infection and limited transmission/Transient infection, expensive and ethical issues	^409^
	Cynomolgus macaques	SARS-CoV-2	3.84 × 10^4^ PFU	IN + IT	0	Limited	Model for pulmonary consolidation and diffuse alveolar damage/limited clinical symptoms, expensive and ethical issues	^411,422,423,901^
		SARS-CoV	10^7^ PFU	IN/IT/IV/IG	0	Mild symptoms	Inflammatory cytokines and chemokines associated with ALI and ARDS, DAD,/Expensive and ethical issues	^415,417–421,902^
		MERS-CoV	10^6^ TCID_50_	IN + IT	0	No	Viral replication and pathology/Lack of disease hallmarks, expensive and ethical issues	^422^
	African green monkeys	SARS-CoV-2	3.84 × 10^4^ PFU	IN + IT	0	Respiratory abnormalities	Thrombocytopenia; type II pneumocyte hyperplasia and alveolar fibrosis; ARDS in aged animals/expensive and ethical issues	
	Baboons	SARS-CoV-2	1.05 × 10^6^ PFU	IN + IT	0	Respiratory abnormalities, fever	More severe clinical manifestations than AGMs and RMs, prolonged virus shedding/Expensive and ethical issues	^424,431^
	Marmosets	SARS-CoV-2	1.05 × 10^6^ PFU	IN + IT	0	No	Insusceptible to SARS-CoV-2	

*NHPs* nonhuman-primates, *SARS-CoV-2* severe acute respiratory syndrome coronavirus 2, *SARS-CoV* severe acute respiratory syndrome coronavirus, *MERS-CoV* Middle East respiratory syndrome, *PFU* plaque formation unit, *TCID*_*50*_ median tissue culture infective dose, *FFU* focus-forming unit, *IN* intranasal, *IT* intratracheal, *OR* oral, *OC* ocular, *IV* intravenous, *IG* intragastric, *hACE2* human angiotensin converting enzyme 2, *ARDS* acute respiratory distress syndrome, *DPP4* dipeptidyl peptidase-4, *ALI* acute lung injury, *AGMs* African green monkeys, *RMs* rhesus macaques, *DAD* diffuse alveolar damage, – not applicable

#### Mice

##### Transgene and humanized mouse model

Receptor knock-in mouse models were generated through transgenic approaches such as microinjection and CRISPR-Cas technology, which yielded precision knock-in or random transgenic plants. Using the lung ciliated epithelial cell-specific HFH4/FOXJ1 promoter, a high SARS-CoV-2 viral load in the lungs was detected in hACE2 transgenic C3B6 mice, and preexposure protection was accomplished.^332^ However, this endogenous promoter-derived transgenic mouse model was not lethal and showed no obvious signs of disease. The promoter of the cytokeratin 18 gene is expressed predominantly in epithelial cells. Transgenic mice expressing hACE2 driven by the promoter of the cytokeratin 18 gene were generated and termed K18-hACE2 mice.^333^ SARS-CoV-infected K18-hACE2 mice developed encephalitis and mild pneumonia. Infection with SARS-CoV-2 and MERS-CoV causes severe disease in the lung and, in some cases, in the brain.^333,334^ Evidence of thrombosis and vasculitis was detected in mice with severe COVID-19 pneumonia. Remarkably, K18-hACE2 mice support SARS-CoV-2 replication in the sinonasal epithelium, which is associated with pathology related to anosmia, a common feature of human disease.^335–337^ In addition, impaired lung function, including respiratory distress, markedly abnormal lung biomechanics, and labored breathing, was observed in K18-hACE2 mice. Moreover, SARS-CoV-2 caused systemic infection in K18-hACE2 mice, and the virus was detected in the nasal epithelium, trachea, lungs, heart, spleen, liver, kidneys, stomach, large intestine, small intestine and brain.^336^ With CRISPR/Cas9 knock-in technology, the mACE2 gene of the C57BL/6 mouse model was completely replaced with hACE2 (termed hACE2 mice).^338^ Viral loads, interstitial pneumonia, and elevated cytokine levels occurred in SARS-CoV-2-infected hACE2 mice. In hACE2 mice, the viral RNA load in the lungs was markedly greater than that in other hACE2 genetically engineered mice generated by pronuclear microinjection, and the distribution of hACE2 in various tissues was more in line with human conditions. Interestingly, intragastric infection with SARS-CoV-2 has been established in hACE2 mice. In addition, SARS-CoV-2-induced acute respiratory illness in transgenic hACE2 model mice exhibited typical pathological changes in the lungs.^339^ This approach is important for the evaluation of vaccines against severe acute respiratory distress syndrome (ARDS). Transgenic mice expressing hCD26/DPP4-based on the exogenous CAG promoter were also generated. This model was susceptible to MERS-CoV infection, resulting in continuous weight loss and death. Infectious viruses were detected in the lungs and brains of mice, coinciding with the activation of genes encoding antiviral and inflammatory mediators.^334,340,341^ In addition, viral RNAs were also detected in the heart, spleen, and intestine, indicating disseminated viral infection. Infected mice develop progressive pneumonia characterized by extensive inflammatory infiltration. The transgenic mouse model was highly susceptible to MERS-CoV, and the 50% infectious dose (ID50) and lethal dose (LD50) of the virus were estimated to be <1 and 10 TCID_50_, respectively.^342^ In contrast to the overwhelming infection observed in the mice challenged with a high dose of MERS-CoV, 10 TCID_50_ infections represented asymptomatic or mild MERS patients.

Based on rapid engineering technology involving the mouse genome, termed VelociGene, humanized mice for DPP4 were generated; these mice express human DPP4 in place of mouse DPP4 without cerebral infection.^343^ This approach preserved the proper expression regulation and protein tissue distribution of DPP4. MERS-CoV could replicate effectively in this model despite the absence of lethality. By combining CRISPR‒Cas9-based genome modification technology and serial passages of MERS-CoV, a mouse-adapted MERS-CoV strain that replicated efficiently in the lungs was established, which caused extreme weight loss, decreased pulmonary function, pulmonary hemorrhage, and pathological signs indicative of end-stage lung disease, particularly ARDS.^344^ Prophylactic and therapeutic countermeasures protected engineered mice against MERS-CoV-induced ARDS. When hDPP4-transgenic mice were generated by microinjection, they were sufficiently susceptible to MERS-CoV infection and exhibited weight loss, decreased pulmonary function, and increased mortality with minimal perturbation of overexpressed hDPP4 after MERS-CoV infection.^345^ In addition, progressive pulmonary fibrosis was observed.

##### Receptor-transduction model

Receptor-transduced models were established by transducing functional receptors in BALB/c and C57BL/6 mice, which facilitated virus entry and infection.^346–348^ Replication-defective adenoviruses are the most extensively used vectors for transduction. Signs such as pneumonia and virus replication in the lungs were observed in hACE2/DPP4-transduced models after Beta-CoV infection. Immunodeficient animals, such as IFNAR^−/−^ and STAT1^−/−^ mice, presented delayed virus clearance, while immunocompetent mice quickly cleared the virus.^349^ In IFNAR knockout mice transduced with AAV-hACE2, monocyte and macrophage recruitment were abolished, CD4^+^, CD8^+^ or NK cell activation was inhibited, and neutrophil and neutrophil accumulation were noted.^350^ Similar results were observed in IRF3/7^−/−^ mice. Ad5-hACE2-transduced STAT1^−/−^ mice are the most susceptible to SARS-CoV-2 infection. To recapitulate the sustained immunopathology of patients with severe COVID-19, Sefik et al. described a humanized MISTRG6 mouse model in which hACE2 was delivered by an adeno-associated virus.^351^ This model maintained an infection period of up to 28 days and exhibited key features of chronic COVID-19 infection, including lung fibrosis, an inflammatory macrophage response, a persistent interferon-stimulated gene signature, and T-cell lymphopenia. Additionally, this model recapitulates the innate and adaptive human immune response. Furthermore, SARS-CoV-2 was shown to replicate in human lung-resident macrophages and drive the disease.^352^ Undergone receptor recognition, human macrophages activate inflammasomes, release proinflammatory cytokines, and promote pyroptosis, thus contributing to immunopathology in severe disease. Conversely, inhibition of the NOD-like receptor family, pyrin domain containing 3 (NLRP3) inflammasome reversed chronic lung pathology, which confirmed the role of inflammasome activation and the corresponding inflammatory response in lung inflammation. Together with the phenomenon that blockade of inflammasome activation leads to the release of SARS-CoV-2 by infected macrophages, it was concluded that inflammasomes oppose host infection by SARS-CoV-2 by producing inflammatory cytokines and pyroptosis to prevent a productive viral cycle. These studies provided evidence that inflammatory macrophages are involved in early infection while driving immunopathology at later stages.

Overall, these viral vector-transduced approaches enabled rapid acquisition of available animal models for emergency needs, but such disease models can only mimic limited physiological features of infection. Another advantage of the Ad5 receptor transduction strategy is that it can be used in genetically deficient mice, facilitating rapid identification of host genes and pathways that play protective or pathogenic roles in disease. However, receptor expression after intranasal AD5 inoculation is restricted to the lungs and may not be targeted to the correct organ. Moreover, the use of anti-vector immunity limits the full application of this animal model.

##### Mouse adapted model

Mouse adapted models for beta-CoVs were established by serial passages of viruses of interest in mcie, which resulted in the accumulation of mutations that increased the virulence of mouse-adapted viruses and enabled viral replication in the lungs, viremia, and dissemination of the virus to extrapulmonary sites, accompanied by lymphopenia, neutrophilia, and pathological changes in the lungs.^353^ The overwhelming extent of viral infection in mice is attributed to lethality. The mDPP4 genomic region encompassing exons 10–12 was replaced with the corresponding genomic region from hDPP4 in hDPP4-KI mice, and a mouse-adapted model was established. After 30 serial passages in KI mice, the titers of mouse-adapted MERS-CoV in the lungs were more than 100 times greater than those of the starting virus, which caused weight loss and fatal infection with little involvement of extrapulmonary tissues.^354^ Compared with the parent virus, mouse-adapted MERS-CoV contains 13-22 mutations, some of which are in the S gene. These S protein mutations sensitized the virus to enter and rendered it more virulent than the parent virus in hDPP4-KI mice.

For SARS-CoV-2, MASCp6 was generated by six successive passages of SARS-CoV-2 in aged BALB/c mice. MASCp6 efficiently infected both aged and young BALB/c mice. It replicates efficiently in the lung and trachea, resulting in moderate pneumonia and inflammatory responses.^355^ A key substitution, N501Y, in the RBD was predicted to contribute to the enhanced infectivity of MACSp6 in mice. In addition, the mouse-adapted SARS-CoV-2 HRB26M strain efficiently infected the upper and lower respiratory tracts of young BALB/c mice and C57BL/6J mice.^356^ Subsequently, a lethal mouse-adapted SARS-CoV-2 MA10 strain, which causes acute lung injury (ALI) in young and aged BALB/c mice, was isolated after ten passages in young BALB/c mice. The epidemiological characteristics of COVID-19, as well as aspects of host genetics, age, cellular tropisms, elevated Th1 cytokines, and loss of surfactant expression and pulmonary function, are linked to the pathological features of ALI.^357^ Interestingly, SARS-CoV-2 MA10 did not cause any mortality in ten-week-old C57BL/6J mice. The process of adaptation introduces multiple point mutations into the viral genome that are responsible for increasing virulence; however, whether this artificially introduced genetic divergence compromises the relevance of the adapted viruses in the first place has yet to be fully elucidated. Lethal mouse-adapted SARS-CoV-2 strains achieved breakthroughs, with 100% fatality and clear mutation sites.^358^ All the mouse-adapted SARS-CoV-2 strains cause more severe disease in aged mice. A potential limitation of the mouse-adapted virus model is that lung disease following infection may rely on species-specific mutations of mouse-adapted strains derived from selective pressures. Such selective pressures may not recapitulate all aspects of human infection.

In mice, adenovirus-mediated transduction of huDPP4 resulted in transient expression of functional receptors in many cells and facilitated MERS-CoV replication in the lungs. A concern with this model is that cells that natively express DPP4 will be infected, and this broader infection of cell types may alter pathogenesis. Moreover, no signs of clinical disease were observed. In the transgene model, when the receptor is under the control of the chicken β-actin promoter, all murine cells express huDPP4, and this nonphysiological expression pattern leads to extensive brain infection and ultimately rapid succumb to infection.^340,359^ However, in hDPP4-Tg mice, the expression of human genes under the control of an endogenous human promoter was not lethal.^360^ There have also been huDPP4 knock-in attempts to replace the mDPP4 ORF with huDPP4 under the control of the endogenous mDPP4 promoter, which ensures correct physiological expression of the knock-in gene and thus provides a more physiological model of human disease.^343^ This lethal model was applied to investigate the host response to MERS-CoV infection.^361^ The depletion of CD8^+^ T cells protected the animals, while the depletion of macrophages exacerbated MERS-CoV-induced pathology and clinical symptoms. That is, the inflammatory response plays an important role in regulating MERS-CoV pathogenesis in vivo. In contrast, mouse models described by Cockrell et al.^344^ and Li et al. were susceptible to infection by serially passaged MERS-CoV, which induced severe lung pathology and diffuse alveolar damage (DAD).^354^ These mice could be good models for studying the pathogenesis of MERS-CoV. The pathogenesis of coronaviruses involves severe acute respiratory infection and immune deregulation; thus, modeling studies should be performed on all cell signals.^362^ Hence, validation of the animal model is crucial. Errors in animal experimental studies narrow the chances of potential drugs, repurposing or repositioning drugs and vaccines for successful translation to the clinic; moreover, resources are wasted. Thus, it is necessary to validate animal models using different criteria, for instance, face, construct, and predictive validation.

#### Syrian hamsters

The Syrian hamster (*Mesocricetus auratus*) is another small mammal model for beta-coronaviruses. Compared with mice, hamsters are naturally susceptible to SARS-CoV and SARS-CoV-2, which is attributed to the differential binding of the spike protein to ACE2 orthologs.^363,364^ Hamsters infected with SARS-CoV or SARS-CoV-2 exhibit severe pulmonary pathological changes associated with an inflammatory response.^365^ In contrast, hamsters are not susceptible to MERS-CoV due to the specificity of DPP4.^366^

SARS-CoV replicates substantially in the respiratory tract and causes pathological changes in the lungs of hamsters. Following IN inoculation, SARS-CoV replication peaked at 3 dpi, and the virus was cleared at 7 dpi. Replication of the virus in the respiratory epithelium at the early stages of infection is accompanied by cellular necrosis, while the inflammatory response corresponds to viral clearance.^367^ Moreover, SARS-CoV-infected hamsters elicited robust NAb responses and were protected against subsequent infection.^367^ In hamsters, a correlation between the level of SARS-CoV in the lungs and the extent of pneumonia was demonstrated.^368^ In addition, Schaecher et al. reported a model of cyclophosphamide-induced immunosuppression caused by SARS-CoV infection in Syrian hamsters. SARS-CoV-infected animals exhibit high morbidity and mortality at approximately 30 dpi.^369^

SARS-CoV-2 infection leads to mild to moderate symptoms in hamsters, including rapid breathing, weight loss, and alveolar damage. High expression of viral nucleocapsid proteins in the airway and intestine and high viral loads in the lung, spleen and lymph node atrophy were noted. SARS-CoV-2 can be efficiently transmitted from inoculated hamsters to naive hamsters by direct contact and aerosols, and infected naive hamsters exhibit similar pathological changes but no weight loss. All infected hamsters showed no mortality and recovered within 14 days.^370,371^ In addition, passive transfer of recovery serum to naive hamsters effectively inhibited viral replication in the lungs, even when serum was given two days after infection.^372^ Hamsters infected with SARS-CoV-2 developed olfactory impairment, similar to the anosmia observed in human patients. Moreover, late-stage SARS-CoV-2 infection in female Syrian hamsters induced cardiovascular disease, including myocardial interstitial fibrosis, ventricular wall, septal thickening, and changes in the serum lipid and metabolite profiles of the hamsters.^373,374^

The severity and mortality of SARS-CoV-2 infection in elderly hamsters were also compared.^375^ The extent of viral replication and the lung inflammatory response were age-dependent, and corresponded to proinflammatory cytokine expression, delayed viral clearance, and aggravated lung injury. Aged hamsters exhibited more pronounced and persistent weight loss, significant alveolar and perivascular edema, pulmonary coagulation abnormalities, and acute kidney injury. Proximal urinary tract damage and mesangial matrix expansion were observed in the kidneys of aged hamsters at the early and late disease stages, respectively. Compared to young hamsters, aged hamsters exhibited prolonged infection.^376,377^ Thus, aged hamsters are suitable models for evaluating the age-associated pathogenesis of SARS-CoV-2 infection. These data suggest that diverse patterns of innate immune response affect disease outcomes in different age groups of hamsters infected with SARS-CoV-2. Gender-dependent SARS-CoV-2 infection was also investigated in hamsters.^378,379^ Adult male hamsters infected with SARS-CoV-2 had greater morbidity, more pronounced weight loss, more severe lung damage, and a slower recovery than infected female animals, despite the absence of mortality. Male hamsters exhibit testicular damage, as indicated by dramatic decreases in sperm count and serum testosterone, and decreased testicular size, weight, and serum sex hormone levels several months after infection. Moreover, these is evidence that a minimum number of SARS-CoV-2 particles remain in the lungs of hamsters recovering from acute COVID-19. Together with persistent weight loss, viral RNA rebound in nasal washings, an early decline in the humoral immune response at 21 dpi, and persistent lung pathogenesis, these results suggest that hamsters could be a model of long COVID-19.^380^

There have also been attempts to establish an hACE2 receptor transgene model under the control of the K18 promoter. A low dose of 100 or 1000 PFU of SARS-CoV-2 resulted in a fatality rate of 66.7–100% in K18-hACE2 hamsters. In addition to severe lesions within the respiratory system, SARS-CoV-2 spreads to the CNS and causes neurological injury.^381^ K18-hACE2 hamsters represent a severe and lethal model of COVID-19 infection.

The Chinese hamster (Cricetulus griseus) is also susceptible to SARS-CoV-2. SARS-CoV-2 infection in Chinese hamsters is associated with lung damage and pneumonia.^382,383^ However, the progression of bronchitis and pneumonia was milder, and the duration of pneumonia was shorter in these patients than in Syrian hamsters. The advantage of using Chinese hamsters is that they experience significant weight loss over a prolonged period following SARS-CoV-2 infection compared to that of the Syrian hamster model.^384^ Like transgenic human ACE2 hamsters, Roborovskii hamsters develop severe respiratory disease after infection with SARS-CoV-2, accompanied by severe acute diffuse alveolar damage and hyaline microthrombi in the lungs. In addition to wild-type (WT) hamsters, several immunodeficient hamster models have been adapted.^385,386^ In addition to the high levels of viral RNA in the blood, spleen, liver, and upper and lower gastrointestinal tract, the lung pathology observed in STAT2^−/−^ hamsters was significantly attenuated.^386^ Moreover, RAG2 knockout (KO) hamsters infected with SARS-CoV-2 via the respiratory tract exhibited more obvious weight loss, an enormous viral load, and even death.^387^ It can be used as a model for severe disease. IL2RG knockout (KO) hamsters infected with SARS-CoV-2 exhibit chronic infection that lasts at least 24 days, resulting in disseminated, moderate to severe, chronic active interstitial pneumonia and active recruitment of neutrophils and macrophages.^385^

#### Ferrets

Ferrets permit the replication of SARS-CoV and SARS-CoV-2 but not MERS-CoV, which is associated with species restriction of the DPP4 receptor.^388^ SARS-CoV and SARS-CoV-2 replicate in the respiratory tract of ferrets and are transmitted through direct contact and aerosols. Ferrets infected with 10^3^ TCID_50_ of SARS-CoV via the IN route presented fever at 2–6 dpi and sneezed at 5–10 dpi. Both viral RNA and infectious virus were detected in the lung and nasal turbinates.^389,390^ In addition, lymphohistiocytic bronchointerstitial pneumonia is a typical histopathological lesion. Bronchial and bronchiolar hyperplasia and perivascular cuffing were observed in the lung tissue of SARS-CoV-infected ferrets.^391^ Simultaneously, increased lymphocytes and macrophages were also observed and were associated with the pulmonary vasculature and the connective tissue surrounding conducting airways.^392^

Compared to SARS-CoV, SARS-CoV-2 replicates mainly in the upper respiratory tract of ferrets. SARS-CoV-2 infection leads to common symptoms, including fever, cough, tiredness, loss of taste or smell, sore throat, headache, aches and pains, diarrhea, a rash on the skin and red or irritated eyes. Serious symptoms, such as difficulty breathing, loss of speech or mobility, and chest pain, were also observed. The virus mainly replicates in the respiratory tract, including the nasal cavity, trachea, bronchi, and lung lobes. Viral RNA and infectious virus were first detected at 3 dpi in nasal turbinate, soft palate, tonsil, and lymphoid tissues. Viral RNA showed transient tissue spread, and infectious antigens were detected only in the nasal epithelium and lymph nodes. Viral shedding was detected in nasal washes, saliva, feces, and urine of infected ferrets until 8 dpi.^393^ Histopathological findings included rhinitis and tracheitis associated with epithelial damage, as well as interstitial or suppurative pneumonia. However, viral RNA and pathology were not observed in most tissues at 14 dpi.^394^ Additionally, alterations in biochemical markers, including increased levels of GLU, ALB, and AST and decreased levels of BUN, CRE, and ALP, were observed in infected animals. However, no significant clinical signs were observed in young ferrets after infection via the IN or intratracheal (IT) route.^395^ Therefore, SARS-CoV-2 infection in young ferrets appears to be a good small animal model for asymptomatic human infection.^396^ Interestingly, the transmissibility of different SARS-CoV-2 variants varies among ferrets. The SARS-CoV-2 beta strain did not replicate in ferrets, whereas the WA1, alpha, and delta strains replicated in the respiratory tract of ferrets. WA1 viruses can be transmitted via direct contact but not via the air, while delta viruses can be transmitted via both the air and direct contact.^397^ The susceptibility of ferrets to SARS-CoV-2 infection was also dose dependent.^398^ After infection, viral RNA was detected in the nasal washes of ferrets in the high-dose (5 × 10^6^ PFU)- and medium-dose (5 × 10^4^ PFU)-infected groups at 1 dpi, and viral RNA shedding peaked at 2–4 dpi and 2–6 dpi, respectively. In contrast, in the low-dose group, 16.7% (1/6) of ferrets (5 × 10^2^ PFU) had detectable viral RNA in their nasal washes. Reduced activity was found in all ferrets at 9 dpi in the high-dose group and one day later in the medium-dose group. Histopathological features of high-dose ferrets included mild necrosis of epithelial cells and inflammatory cell infiltration in the nasal cavity, multifocal bronchopneumonia, interstitial pneumonia, and proliferation of type II pneumocytes. In the medium-sized group, mild multifocal bronchopneumonia and bronchopneumonia were observed in no more than 5% of the lung sections. Inflammatory cell infiltration in the liver portal areas was more severe in animals in the high- and medium-dose groups than in the animals in low-dose group. Consequently, a high dose of SARS-CoV-2 and aged animals may be needed in a COVID-19 ferret model.

Overall, only a small portion of the ferrets exhibited obvious clinical symptoms throughout the infection course. The susceptibility of ferrets to SARS-CoV-2 infection was age-dependent. Aged ferrets presented higher virus loads and longer shedding in respiratory secretions than young animals did due to higher expression levels of ACE2 and TMPRSS2, receptors of virus entry, in the upper respiratory tract.^399^ Moreover, the expression of type I interferons, activated T cells, and M1 macrophage response genes was strongly upregulated in aged ferret lungs, which is in agreement with what has been observed in patients with severe COVID-19.^400^ Overall, compared with young ferrets, aged ferrets more accurately mimicked disease features.

#### Minks

Mink (Neovison vison), a member of the Mustelidae, has previously been used as an animal model for SARS-CoV infection.^401^ During the COVID-19 pandemic, SARS-CoV-2 was transmitted to minks in the Netherlands, Denmark and other European countries. SARS-CoV-2 is highly transmissible in minks through direct contact and respiratory droplets.^402,403^ Importantly, SARS-CoV-2 variants have been found in minks, and relevant transmission from minks to humans has also been confirmed.^404^ Minks are highly susceptible to SARS-CoV-2 due to their functional receptor ACE2.^405,406^ Clinical manifestations of SARS-CoV-2 infection in minks were dependent on the challenge dose and virus strain. Minks intranasally inoculated with 5 × 10^6^ PFU of the original SARS-CoV-2 strain developed severe pathological injury in the respiratory tract and caused up to 20% weight loss.^403^ Minks intranasally infected with the SARS-CoV-2 Omicron variant at a dose of 4 × 10^5^ PFU developed mild to moderate clinical signs, including lethargy, diarrhea, nasal signs and sneezing.^407^ In contrast, mink intratracheally challenged with 10^6^ TCID50 of SARS-CoV-2 Omic mimicked the pathological features of severe COVID-19. Notably, the viral RNA concentration reached 7.15 log_10_ RNA copies/mL in nasal lavage fluid and 6.73 log_10_ RNA copies/mL in throat swabs at 1–2 dpi. In addition to the respiratory tract, viral RNA was also detected in the lungs, brains, and eyeballs of infected animals. Pathology revealed diffuse alveolar damage, extensive edema or fibrin exudation in the alveolar lumina, and infiltration of activated macrophages and neutrophils, recapitulating lung signatures observed in COVID-19 patients. Additionally, inflammatory responses were also observed in the gastrointestinal tract, liver, heart, and kidney.^408^

#### Nonhuman primates

##### Rhesus macaques and common marmosets

Rhesus macaques and common marmosets are susceptible to MERS-CoV infection. MERS-CoV caused transient lower respiratory tract infection in rhesus macaques, whereas a more severe disease course was observed in infected common marmosets.^409,410^ SARS-CoV-2 infection in rhesus macaques leads to mild clinical disease and abundant viral replication in the respiratory tract. Fever, weight loss, decreased appetite, and hypoxia are commonly reported symptoms. In some cases, abnormal blood indices, such as a decrease in platelet count, transient neutropenia, and lymphopenia, has also been reported.^411,412^ Histopathological lesions included pulmonary discoloration, consolidation, hyperemia, glass opacity, infiltrates, hemorrhage, scarring, necrosis, and interstitial pneumonia.^413^ Lesions in the liver and spleen were also noted. Although rhesus macaques most closely recapitulate human symptoms of COVID-19, some typical clinical symptoms, including acute respiratory distress syndrome, were not observed, which limits their application in detailed studies of COVID-19.

SARS-CoV mainly infects bronchial epithelial cells and type-1 and type-2 pneumocytes in the respiratory tract of cynomolgus macaques and causes multiple foci of acute DAD.^414^ Mild symptoms such as cough, mild breathing difficulties, reduced food and water intake and decreased activity were observed.^415,416^ Viral RNA was detected in both nasal swabs and oral swabs at 3–5 dpi. Respiratory and conjunctival SARS-CoV infections induced unifocal and multifocal pneumonia at 8–10 dpi.^417^ Although there are dramatic differences in the number of host genes regulated during infection with different SARS-CoV strains, the major genes associated with the inflammatory response are similar.^418^ Inflammatory cytokines and chemokines, such as IL-6, IL-8, IFN-γ, CXCL1, CXCL2, and CXCL10, which are associated with acute lung injury (ALI) and ARDS, were induced after infection in cynomolgus macaques.^419,420^ In addition, investigations of the challenge route have been performed in cynomolgus macaques. Challenge with the HKU39849 isolate of SARS-CoV via IN, intravenous (IV), or intragastric (IG) route did not affect the lower respiratory tract, whereas IT inoculation induced lung lesions, which are associated with the replication of SARS-CoV in alveolar cells, macrophages, neutrophils, and cytokine responses. In addition, high levels of IL-8 and TNF-α, which are elicited by activated alveolar macrophages and neutrophils and are responsible for lung lesions, were detected in the lungs and peribronchial lymph nodes of IT-inoculated macaques.^421^ IN and IT inoculation of cynomolgus macaques with MERS-CoV did not cause clinical signs. Viral RNA was first detected at 1 dpi and peaked at 1 and 2 dpi in nasal and throat swabs. Additionally, a low level of viral RNA was observed in rectal swabs at 2–3 dpi. MERS-CoV RNA was detected mainly in the respiratory tract of infected macaques. Foci pulmonary consolidation was characterized by mildly depressed areas in the lungs. Histopathological features in the lungs manifested as typical DAD, which was consistent with the replication of the virus in the lower respiratory tract.^422^

##### Cynomolgus macaques

SARS-CoV-2 replicates mainly in the upper and lower respiratory tracts, and viral infection does not cause lethal outcomes in cynomolgus macaques, which exhibit mild to severe pneumonia, fever, viral shedding, respiratory abnormalities, immune cell infiltration, or inflammatory response. Pathological changes mainly included pulmonary consolidation and diffuse alveolar damage.^423^ Recent studies have compared susceptibility to SARS-CoV-2 among cynomolgus macaques, rhesus macaques (RMs), common marmosets (CMs), and AGMs.^422^ After exposure to SARS-CoV-2 at a mean dose of 3.84 × 10^4^ PFU via the aerosol route, AGMs, RMs, and CMs presented respiratory abnormalities and viral shedding. After infection, CMs developed fever, and AGMs and RMs develop thrombocytopenia. Type II pneumocyte hyperplasia and alveolar fibrosis were more frequently observed in challenged AGMs and CMs. CMs developed consistent disease and exhibited the most severe clinical manifestations among these three macaques.^424^ SARS-CoV-2 was intratracheally and/or intranasally infected into macaques and mainly replicated in the upper and lower respiratory tract, causing pulmonary abnormalities, fever, and weight loss in both cynomolgus macaques and rhesus macaques, whereas fever was observed in only half of the infected common marmosets. Compared with the other two monkeys, rhesus macaques presented the highest levels of inflammatory cytokine expression and pulmonary pathology changes after infection.^411^ Therefore, differences in virus strain, dose, and route of challenge might cause significant differences in the clinical signs of infection in macaques.^425^ In addition, a head-to-head study showed that SARS-CoV-2-challenged cynomolgus macaques and rhesus macaques did not exhibit significant differences in weight loss or body temperature changes. In addition, these two species exhibited similar histopathological changes, including alveolar necrosis, type II pneumocyte hyperplasia, and interstitial lymphoid infiltrates.^426^ Overall, these two species responded similarly to SARS-CoV-2 infection.

The severity of SARS-CoV-2 infection in cynomolgus macaques was age-associated. After exposure to SARS-CoV-2, aged cynomolgus macaques presented greater viral RNA levels and longer infection periods than young animals. After inoculation with SARS-CoV-2 via IT or IN route, viral RNA was detected in nasal or throat swab samples, respectively, and the levels peaked at 1 and 2 dpi, respectively, in young cynomolgus macaques, whereas both types of RNA peaked at 4 dpi in aged animals. Additionally, a greater level of viral RNA in nasal swabs were observed in aged animals than in young animals.^422^ Decreased white blood cell and platelet counts were observed in both young and aged cynomolgus macaques. However, significant weight loss was not observed in these two groups.^427^ Moreover, proteomic and metabolomic analyses revealed that neutrophilia, lymphopenia and cytokine storms were significantly weakened in SARS-CoV-2-infected cynomolgus macaques, which was consistent with reports of clinical symptoms in patients with moderate COVID-19.^428^

In SARS-CoV-2-challenged African green monkeys, transient fever, decreased appetite, hypercapnia, lymphocytopenia and thrombocytopenia, elevated liver-related enzymes, and increased monocytes were observed.^424,429,430^ Like in other NHPs, viral pneumonia, severe pulmonary consolidation with hemorrhage and infiltration, extensive pulmonary lesions and gastrointestinal abnormalities were also observed in infected animals. Common histopathological lesions included pulmonary discoloration, opacity, bronchiolization, hyperemia, and pleural adhesions.^429^ Compared to rhesus macaques, African green monkeys exhibited more severe consolidation and edema in the lung lobes.^424^ In particular, ARDS, a common and often fatal characteristic that is difficult to replicate in other NHPs, was sustainably observed in aged African green monkeys. African green monkeys, especially aged animals, are useful models for severe COVID-19.^430^

##### Baboons

Compared with macaques, baboons are more susceptible to SARS-CoV-2 infection and exhibit more severe histopathological lesions, prolonged viral RNA shedding and substantially more lung inflammation.^431^ In contrast, marmosets are less susceptible to severe acute respiratory syndrome 2 (SARS-CoV-2) than macaques or baboons.^431^ Preclinical studies in the NHPs of patients with COVID-19 revealed immune correlates of protection and simultaneously provided remarkable predictive value for the outcome of clinical efficacy studies of COVID-19 vaccines.^432–434^ Moreover, studies in NHPs accurately reflected that protection against symptomatic COVID-19 infection would be easier to achieve than protection against viral replication in the upper respiratory tract.^435^ According to the NHP model, more viral breakthroughs were observed following beta VOC challenge than following homologous WA1/2020 challenge.^436^ Overall, rhesus macaques are the most common NHPs for COVID-19 infection because they are commercially available and manifest clinical symptoms quite well. Cynomolgus macaques usually exhibit pulmonary consolidation but show weak clinical symptoms. In contrast, African green monkeys generally exhibit severe symptoms, but their scarcity greatly limits their use. Nevertheless, natural protective immunity, such as innate, humoral, and cellular immune responses, can be induced in these NHPs.

### Medical countermeasures for beta-coronavirus diseases

Globally, no vaccines or therapies have been approved for SARS-CoV or MERS-CoV. Approved COVID-19 vaccines are based on both traditional and novel techniques. Here we provide insights into advanced vaccines and approved therapies for COVID-19, especially antibodies and small molecule drugs (Table 7).

**Table 7:** Representative medical countermeasures for beta-coronaviruses

Classification	Manufacturer	Name	Platform/Strategy	Stage	Efficacy/Benefic	References
Vaccines	Sinopharm/Sinovac Biotech	CoronaVac/ BBIBP-CorV	Whole virus inactivated vaccines	Licensed	50% protective efficacy	^903–905^
	CanSinoBIO	Convidecia	Encoding the S of SARS-CoV-2 based on Ad5	Licensed	57.5% protective efficacy; aerosolized	^476–478^
	Wantai Biological	dNS1-RBD	Encoding the RBD of SARS-CoV-2 based on IFV	Licensed	28.2% protective efficacy and applicable in boosting	^473^
	Johnson	Ad26-S	Encoding the RBD of SARS-CoV-2 based on Ad26 vector	Licensed	52.9% protective efficacy	^432,455,479^
	Russia	Ad26 + Ad5-S (SPUTNIK V)	Prime-boost strategy of two viral vector vaccines	Licensed	91.4% protective efficacy	^456^
	AstraZeneca	ChAdOx1-S	Encoding the S gene of SARS-CoV-2 based on ChAdOx1 vector	Licensed	70.4% protection protective efficacy	^456^
	Modena/BioNTech	mRNA-1273/ BNT162b2	Encoding the S gene of SARS-CoV-2 based on mRNA platform	Licensed	90% protective efficacy	^490,492,906,907^
	Clover Biopharmaceuticals	SCB-2019	S-Trimer	Licensed	67·2% protective efficacy	^443^
	Novavax	NVX-CoV2373	S-Trimer	Licensed	90.4% protective efficacy	^445,908,909^
	Zhifei Longcom	ZF2001	RBD-dimer	Licensed	75.7% protective efficacy	^449,450^
Antibodies	Genentech	Tocilizumab (Actemra)	Humanized mAb targeting IL-6R and inhibit subsequent signal transduction	Licensed	Improve survival and other clinical outcomes	^501,502^
	Eli Liliy and AbCellera	LY-CoV555 (bamlanivimab)	Human antibodies targeting S1	Licensed	Reduces viral load, disease symptoms, and hospitalization and emergency treatment risks	^505,910^
	Junshi Biosciences	LY-JS016 (LY-CoV016)				
	Regeneron Pharmaceuticals	REGN-COV2	RBD-targeting antibody cocktail	Licensed	Reduces the viral load and alleviates symptoms in non-hospitalized COVID-19 patients	^506,509,510^
	BriiBio	Amubarvimab/Romlusevimab	Human antibodies targeting S1	Licensed	Reduced the hospitalization and mortality risk by 80%	^515^
	China	4A8	nAbs targeting the NTD	Clinical	Did not compete with antibodies targeting other regions of the S protein	^517^
Small molecule drugs	Gilead technology	Remdesivir (GS-5734)	RdRp inhibitor	Licensed	Not sure efficacy of remdesivir in treating COVID-19	^532,533^
	Merck	Molnupiravir	RdRp inhibitor	Licensed	Higher proportion of viral RNA clearance and infectious virus elimination in clinical trials	^537^
	Junshi Biosciences	VV116	RdRp inhibitor	Licensed	Clinical benefits	^539^
	Pfizer	Paxlovid (Nirmatrelvir+Ritonavir)	Mpro inhibitor	Licensed	Reduce hospitalization and mortality rates in patients	^542^
	Incyte	Baricitinib	JAK signal pathways inhibitor	Licensed	Reduced mortality in hospitalized patients	^545^
	Kintor Pharmaceutical	Proxalutamide	Androgen receptor antagonist	Licensed	Higher recovery rate and lower all-cause mortality rate; reduced the rate of hospitalization by 91%	^548,549^

*S* spike, *SARS-CoV-2* Severe acute respiratory syndrome coronavirus 2, *Ad5* Adenovirus type 5, *RBD* Receptor binding domain, *IFV* Influenza virus, *Ad26* Adenovirus type 26, *ChAdOx1* Chimpanzee adenovirus type 1, *mAb* Monoclonal antibody, *IL-6R* IL-6 receptor, *NTD* N-terminal domain, *RdRp* RNA dependent RNA polymerase, *Mpro* Main protease, *JAK* Janus kinase

#### Preventive vaccines for beta-coronavirus diseases

##### Inactivated vaccines

Inactivated vaccines are traditional platforms that use radiation or chemical substances to inactivate pathogens of interest under eligible biosafety conditions. More than three inactivated COVID-19 vaccines have been approved. These vaccines are immunogenic, capable of inducing S protein or N protein-specific antibodies and nAbs and are safe in clinical trials.^437–439^ Inactivated vaccine platforms provide timely choices for safe vaccines during emergencies. Exposure to complete antigen epitopes of coronaviruses enables the inducing of immune responses other than those involving S protein. However, inactivated viruses lack pathogen-associated molecular patterns (PAMC) and therefore can not simulate the natural process of viral infection. Also, inactivated viruses cannot arouse mucosal immune response and thus show limited ability to block transmission.

##### Protein subunit vaccines

Subunit vaccines were developed by obtaining immunogenic proteins or peptides from pathogens. Compared with traditional approaches that obtain monomeric immunogenic proteins from eukaryotic expression system, novel techniques have facilitated antigen assembly and display in various forms.^440^ For example, the S protein with a stabilized trimeric form (S-Trimer) has been widely investigated in vaccine designs for SARS-CoV and MERS-CoV and has been proven to be safe and immunogenic.^441,442^ Clover Biopharmaceuticals developed an S-Trimer subunit vaccine named SCB-2019.^443^ Coupled with CpG/Alum adjuvants, an overall protective efficacy of 67·2% was reported in phase III clinical trials.^444^ NVX-CoV2373, which was developed by Novavax, is another S trimers-based COVID-19 vaccine tha has been approved.^445^ To develop a universal vaccine for both beta-CoVs. A dimeric form of MERS-CoV RBD was described, which significantly increased NAb titers compared to conventional subunit vaccine approaches. The structure guided design enabled the RBD dimer to fully expose receptor-binding motifs and yielded a stable version of RBD-dimer.^446^ Similarly, another tandem-repeat dimeric RBD protein-based COVID-19 vaccine, known as ZF2001, was developed and approved.^447^ In subsequent attempts, it was proven that antigens from novel variants, for example, the delta-omicron chimeric RBD-dimer, could better adapt to prevalent variants and elicite broader serum neutralization of SARS-CoV-2 variants.^448^ Additionally, these RBD-Dimer vaccines, such as, BQ.1, BQ.1.1, an XBB, have been shown to have broad neutralizing effects on SARS-CoV-2 variants. In clinical trials, a three-dose regimen of ZF2001 was found to be safe and responsible for a protective efficacy of 75.7% for at least 6 months.^449,450^ There are also reports of structure-based nanoparticle vaccines that display 60 copies of the SARS-CoV-2 RBD in a highly immunogenic array.^451^ Compared with inactivated vaccines, protein subunit vaccines are also safe, simultaneously, they repeatedly and adequately display of immunogenic proteins, which promises a broader spectrum of protection. However, these vaccines are also weak inducers of mucosal immune response.

##### Viral vector vaccines

Viral vector vaccines are replication-competent/deficient viral particles whose genomes have been modified to carry foreign genes encoding the targeted antigens for infectious disease without the involvement of hazardous pathogens. Viral vector vaccines can simulate the natural infection process of specific pathogens, thus triggering robust innate, mucosal, humoral and cellular immunity against infectious diseases and providing choices for pathogens that hamper control efforts using conventional vaccine approaches.^452^ Notably, due to the expression of diverse PAMPs, viral vectors have intrinsic adjuvant properties that effectively activate innate immunity in the absence of adjuvants.^453^ Several viral vector-based prophylactic vaccines have entered Phase III clinical trials or have been approved for use against beta-CoVs.^454–458^

In attempts to use a VSV vector for Beta-CoVs, single dose-vaccination with VSV-based vaccines induced robust and long-lasting immune responses.^459–462^ Notably, VSVΔG-based vaccine designs exhibit altered tropism, which enables mucosal delivery routes such as oral and intranasal vaccination and induces a mucosal immune response. Parainfluenza viruses (PIVs) were also hopeful viral vectors for Beta-CoVs. Several PIV vectors have been investigated, including human parainfluenza virus type 2 (hPIV2), human parainfluenza virus type 3 (HPIV3), parainfluenza virus type 5 (PIV5) and chimeric bovine/human PIV consisting of the bovine PIV3 (BPIV3) strain Kansas, in which the BPIV3 HN and F glycoproteins have been replaced by those of the human PIV3 strain JS (B/HPIV3).^463,464^ PIV-based vaccines were designed to expressing full-length prefusion-stabilized or conventional S protein of Beta-CoVs.^465,466^ A single IN dose vaccination of these replication-defective/competent constructs induced high levels of S-specific IgG and mucosal IgA antibodies in animal models.^467–470^ Several live-attenuated influenza virus (IFV) vectored COVID-19 vaccines have been developed. dNS1-RBD is an approved COVID-19 vaccine, in which the SARS-CoV-2 RBD gene was inserted in place of IFV NS1 by gene reassortment.^471,472^ dNS1-RBD induced rapid, long-term, and broad-spectrum immune responses, particularly local resident memory T cells in the respiratory tract.^472^ In clinical trials, IN inoculation of dNS1-RBD was tolerated in healthy adults;^457^ however, humoral and mucosal immune responses against SARS-CoV-2 were weak. The overall protective efficacy of dNS1-RBD was 28.2%.^473^

The Ad5-nCoV vaccine was designed to deliver the S gene of SARS-CoV-2, which is well tolerated in humans and induces S-specific antibodies and nAbs, and a T-cell response.^474,475^ In further attempts, heterologous boost immunization with aerosolized Ad5-nCoV following two-dose priming with an inactivated COVID-19 vaccine was proven to be safe and highly immunogenic compared to the homologous prime-boost strategy.^476–478^ Similarly, Harvard Medical School and Russia constructed an Ad26-vectored and Ad26 plus Ad5 vector COVID-19 vaccine.^432,455,479^ The University of Oxford developed a ChAdOx1-vectored COVID-19 vaccine.^480,481^ The protective efficacy of these Adv based COVID-19 vaccines is 50–70.4%.^456,458,482^ For adenovirus vector-based vaccines, preexisting antiviral immunity is the main obstacle to address. Poxvirus vectors have attracted increasing attention for their ability to induce long-lasting T-cell immune responses.^483^ One of the most paramount poxvirus vectors is modified vaccinia virus Ankara (MVA). Single-dose vaccination of MVA-vectored coronavirus vaccines expressing the S protein induced high levels of NAbs.^484–486^

##### Nucleic acid vaccines

Nucleic acid vaccines can be further divided into DNA vaccines and mRNA vaccines. mRNA vaccines are more popular due to their simple production process/industrialization, flexibility to be edited, and ability to induce a better immune response.^487^ mRNA-1273 and BNT162b2 were wildly distributed during the COVID-19 pandemic. These mRNA vaccines are modified and delivered via lipid nanoparticle systems. Compared with traditional vaccines, mRNA vaccines efficiently translate the encoded mRNA in-vivo, and enable cell-free and scalable productions.^488^ Both mRNA-1273 and BNT162b2 encode the perfusion-stabilized S protein, which induces robust SARS-CoV-2 nAbs and achieves protection in airways in animal models.^433,489–491^ In clinical trials, they were highly immunogenic and offered a protective efficacy of more than 90%.^490,492^ Lipid nanoparticle-encapsulated mRNA (mRNA-LNP) encoding the RBD of SARS-CoV-2 was also developed (termed ARCoV).^493^ In Phase I clinical trial, ARCoV was shown to be safe and immunogenic, which warrants further large scale clinical testing.^494^

#### Therapies for Beta-coronavirus diseases

##### Passive serum therapy

Passive serum therapy has been clinically applied for treating SARS, MERS, and SARS-CoV-2.^495^ Prior to the successful development of effective drugs, CPT has become an active clinical intervention method for the treatment of COVID-19. There have been reports of COVID-19 patients with a good prognosis who underwent CPT. CPT showed ability in antiviral, immunomodulatory, anti-inflammatory, and antithrombotic, making it a potentially effective approach for treating viral infections. On the other hand, it should be noted that CPT can cause moderate to severe transfusion-related reactions, including fever, allergies, life-threatening bronchospasm, acute lung injury, and increased circulatory load, in patients with kidney and cardiovascular disease. Patients undergoing CPT should be continuously monitored to ensure a timely response to possible adverse reactions.^496^ Additionally, there are still administrative and technical barriers to the application of CPT, including the availability, screening, approval, collection and monitoring of blood donors and the allocation of a sufficient number of laboratory facilities.

##### Neutralizing antibodies

Compared with plasma therapy, neutralizing antibodies show better specificity and safety, and can be applied in both prevention and treatment.^497,498^ Antibodies can be classified according to their source, structure, and method of acquisition. Neutralizing antibodies are important therapeutic approaches for treating COVID-19, particularly severe cases of COVID-19 in clinical. Antibodies bind to antigens through Fab, blocking the entry of pathogens into cells. Fc can also cause ADCC, ADCP, and complement-dependent cytotoxicity (CDC), and in some cases, induce antibody-dependent enhancement (ADE). There are also antibodies that target dysregulated immune responses, termed nontargeted monoclonal antibodies.

Nontargeting mAb drugs usually act as immune modulators in the treatment of severe cases. Potential targets, including interleukin-6 receptor (IL-6R)/IL-6, tumor necrosis factor, and human epidermal receptor 2 (HER2) have been described. Previous studies have shown that IL-6 is a proinflammatory cytokine, and its release can induce a series of downstream proinflammatory responses, which serve as an important pathway for inducing cytokine storms in COVID-19 patients.^499,500^ In response, tocilizumab was designed to alleviate cytokine storms in COVID-19 patients. Tocilizumab is a recombinant humanized mAb that can specifically bind to the interleukin-6 receptor (IL-6R), thus inhibiting subsequent signal transduction.^501^ The FDA approved tocilizumab because of its potential benefits in clinical trials to improve survival and other clinical outcomes.^502^ However, the therapeutic efficacy of tocilizumab is controversial, since some clinical trials have suggest that tocilizumab does not result in a significantly better clinical outcome or lower mortality.^503,504^

SARS-CoV-2 specific NAbs mainly target four regions within the S protein, including the N-terminal domain (NTD), RBD, stem helix region and fusion peptide region.^497^ S1-targeting NAbs work by blocking receptor-ligand recognition. LY-CoV555 (bamlanivimab) was the first FDA-approved NAb developed by Eli Liliy and AbCellera. Compared to placebo, a single intravenous injection of LY-CoV555 was associated with limited benefits. In another study, combination therapy with LY-CoV555 and LY-CoV016 (JS016) significantly reduced the viral load, disease symptoms, and hospitalization and emergency treatment risks in mild and moderate COVID-19 patients.^505^ REGN-COV2 is another RBD-targeting antibody cocktail developed by Regeneron, that is composed of the REGN10987 and REGN10933 antibodies. REGN-COV2 was obtained by matching the antibody genes from the VelocImmune mice with those from the convalescent patients.^506^ REGN10933 and REGN10987 which are ideal partners against the development of escaped virus mutants in response to selective pressure from a single antibody, which were selected as non-competing highly-potent binding to the RBD of SARS-CoV-2,.^507^ Preclinical studies have shown that REGN-COV2 can reduce the viral load and associated damage in the lungs of nonhuman primates in rhesus monkeys and golden hamsters.^508^ According to Phase III clinical trials, REGN-COV2 reduces the viral load and alleviates symptoms in nonhospitalized COVID-19 patients.^509,510^ JS016 (etesevimab) was developed by Junshipharma. Initially, CA1 and CB6 were isolated from COVID-19 convalescent patients.^511^ Among them, CB6-LALA, also known as JS016 mAb, showed stronger neutralizing activity, and L234A and L235A were introduced into the Fc segment to remove the ADCC effect. In rhesus monkeys, CB6-LALA showed therapeutic and preventive effects. In this phase 3 trial among high-risk ambulatory patients, bamlanivimab plus etesevimab led to a lower incidence of hospitalization and death and accelerated the decline in the SARS-CoV-2 viral load.^512,513^ Amubarvimab/Romlusevimab (BRII-196 plus BRII-198) are mAbs approved for the treatment of COVID-19 by the National Medical Products Administration of China.^514^ In Phase III clinical trial, BRII-196 plus BRII-198 treatment reduced the hospitalization and mortality risk by 80%.^515^ However, the results of the phase III clinical trial of the ACTIV-3 clinical study showed that there was no significant difference between the protection rate of the combination therapy of mAb and the placebo for severe hospitalized patients with COVID-19, and this result was the same as that of GlaxoSmithKline’s neutralizing antibody against COVID-19. However, BRII-196 plus BRII-198 showed no efficacy in improving clinical outcomes among adults hospitalized with COVID-19.^516^

4A8 is one of the earliest identified nAbs targeting the NTD.^517^ Among the five structural loops in the NTD (N1–N5), 4A8N3 interacted with N3 and N5. Similarly, other NTD-targeting mAbs, such as COV2-2676, COV2-2489, 4–8, and 5–24, also recognize structural loops.^518,519^ Importantly, since NTD-targeting antibodies do not compete with antibodies targeting other regions of the S protein, antibody cocktails combining NTD-targeting antibodies with non-NTD-targeting antibodies are feasible. Interestingly, it has been reported that potent NTD-directed neutralizing antibodies appear to target a single supersite, which is defined as the strongly positively charged epitope in the NTD.^520,521^

In contrast to those in the S1 subunit, neutralizing epitopes in the S2 subunit are more conserved. S2-targeting NAbs can inhibit SARS-CoV-2 infection by preventing fusion mediated by S protein. Representatively, S2P6 is a humanized S2 subunit-targeting mAb that exhibits broad-spectrum neutralization of beta-CoVs.^522^ S2P6 bound to 14 residues in the S2 stem helix (SH) region, and these epitopes were conserved across beta-CoVs. The Fc effector functions of S2P6 also play a critical role in virus clearance. S2P6 can broadly neutralize beta-CoVs, with IC50 values of 1.4 μg ml^−1^ for SARS-CoV-2, 2.4 μg ml^−1^ for SARS-CoV, and 17.1 μg ml^−1^ for MERS-CoV. Apart from the SH region, S2 fusion peptides (FPs) are also highly conserved among coronavirus genera and corresponding NAbs, such as VN01H1 and VP12E7, have also been developed.^523^ Interestingly, this type of antibody binds S2 FPs without competition with S2 SH-targeting antibodies, highlighting the possibility of combining S2 SH and S2 FP bispecific antibodies.^523^ The synergistic effects of S2 FP-targeting and RBD-targeting nAbs have also been confirmed.^524^ Although S2-targeting NAbs showed inferior neutralizing potency compared with that of RBD-targeting antibodies, S2 has the potential to be a broad-spectrum neutralizing.^525^

Overall, NAb treatment significantly reduced the risk of hospitalization and/or death among nonhospitalized adults with mild to moderate SARS-CoV-2 infection at high risk for progression to severe disease, but showing limited efficacy in treating severe cases. Another concern for developing neutralizing antibodies is escape by variants. Representatively, the Omicron variant of SARS-CoV-2 escapes more than 85% of 247 human SARS-CoV-2 neutralizing antibodies.^526^

In addition to the use of antibody cocktails, another promising method is to engineer them into bispecific or even multispecific antibodies, which confer synergistic neutralization. In addition to increasing the threshold for the generation of neutralization escape mutants, the dual antibody has a cost advantage over the mAb cocktail strategy, and the complex formulation of the mAb cocktail usually increases production costs and affects yield. For example, different RBD class antibodies or RBD-targeting antibodies can be combined with NTD-targeting and/or S2-targeting antibodies. Such attempts have already shown the ability to avoid escape and enhance neutralizing ability.^527^ Overall, antibodies in RBD class 1 and class 2 in the NTD supersite are more prone to mutation and consequently lose their neutralizing activity. In contrast, antibodies targeting S2 epitopes are more conserved. Therefore, balance between the breadth and potency of nAbs is needed. On the other hand, comprehending the characteristics of these bnAbs could provide guidance for devising more effective vaccines focused on conserved viral epitopes for the development of broad-spectrum antibody therapies. However, clinical research on antibody drugs has focused mainly on preventive efficacy, while therapeutic applications are limited due to the lack of animal models that accurately simulate human infection.

Bispecific nanobodies against SARS-CoV-2 have also been reported.^365^ These nanobodies could reach the conserved and cryptic epitope of SARS-CoV-2. This small volume of bispecific nanoantibodies has a molecular size of only 27 kDa. Compared to traditional mAb drugs, which target the internal region of S trimer, nobodies can enter and bind tightly to the hidden epitope.^528^ Together with their high tissue penetration ability, bispecific nanobodies can be formulated as an inhalable preparation that shows improved lung targeting.

##### Small molecule drugs

Compared to monoclonal antibodies, small molecule drugs are more flexible in binding to target molecules.^529^ Antiviral strategies against SARS-CoV-2 were explored in several aspects, including direct inhibition of key viral proteins such as RdRp and Mpro, interference with host enzymes such as ACE2 and proteases, and blocking relevant immunoregulatory pathways.^530^

##### Targeting RdRp

Remdesivir was previously developed for the prevention of EBOV. It is the first FDA-approved drug for COVID-19, it functions as a nonobligate and inhibits RNA-dependent RNA polymerase (RdRp).^531^ Structurally, the RNA template is inserted into the central channel of the RdRp, where remdesivir mimics a nucleotide. It is covalently incorporated into the replicating RNA at the first replicated base pair, and terminates chain elongation, thus blocking further synthesis of SARS-CoV-2 RNA.^532^ In clinical, there is some controversy over the efficacy of remdesivir in treating COVID-19.^533^ In nonhospitalized patients, a 3-day course given of remdesivir were proven to be safe and was responsible for an 87% lower risk than a placebo.^534^ In COVID-19 patients with or without or conventional oxygen support, remdesivir reduced mortality. For patients with more respiratory support or acquired immunity, the cost-effectiveness of remdesivir remains to be further elucidated.^535^ Like remdesivir, molnupiravir is another RdRp inhibitor developed by Merck that was approved for the treatment of patients with mild to moderate COVID-19.^536^ As a mutagenesis inducer, Molnupiravir was associated with increased proportion of viral RNA clearance and infectious virus elimination in clinical trials.^537^ In another clinical study, early treatment with molnupiravir after the onset of symptoms reduced the risk of hospitalization.^538^ VV116 is an oral antiviral agent with potent activity against COVID-19 that has been approved in China. Among adults with mild to moderate COVID-19 who were at risk for progression, VV116 showed efficacy comparable to that of nirmatrelvir-ritonavir, with fewer safety concerns.^539^ Three other RdRp inhibitor drugs, favipiravir (AVIFAVIR), and azvudine have also been approved.^540,541^ Moreover, far more RdRp-targeting small molecules are under investigation.

##### Targeting Mpro

Paxlovid is a popular oral small molecule COVID-19 drug that is composed of PF-07321332 (nirmatrelvir) and ritonavir. Wherein nirmatrelvir corresponds to Mpro inhibition, while ritonavir further improves efficacy by slowing the metabolism of Nirmatrelvir and maximizing its therapeutic benefits. Compared to placebo, paxlovid can significantly reduce hospitalization and mortality in high-risk patients with moderate to severe COVID-19 by 89 and 88%, respectively, within 3 or 5 days of symptom onset.^542^

##### Targeting proinflammatory signal pathways

Baricitinib is an approved oral selective inhibitor of JAK signaling pathways. It blocks cytokine-induced JAK/STAT/APOL1 signaling.^543^ Baricitinib improved the clinical outcomes of patients with COVID-19, reshaped the immune landscape, and alleviated the immunosuppressive effects in myeloid cells, consequently restraining the immune dysregulation.^544^ In Phase III clinical trials, treatment with baricitinib in addition to standard of care reduced mortality in hospitalized COVID-19 patients although not significantly.^545^ In clinical practice, remdesivir has been approved by FDA for the treatment of mild to moderate COVID-19 patients while Baricitinib, was approved for the treatment of severe hospitalized patients with COVID-19.^546,547^ Combination treatment with the baricitinib and remdesivir was safe and superior to remdesivir alone for the treatment of hospitalized COVID-19 patients. In addition to the JAK-STAT pathway, other COVID-19 associated proinflammatory signaling pathways, such as the BTK, NF-κB and NLRP3 pathways, have also been identified as targets.^530^

##### Receptor antagonist

Proxalutamide is an approved androgen receptor antagonist against SARS-CoV-2.^548^ Compared with the placebo, proxalutamide was associated with a greater recovery rate and lower all-cause mortality at day 14 and day 28, respectively.^548^ In another study, proxalutamide treatment reduced the rate of hospitalization by 91%.^549^ Furthermore, the anti-COVID-19 effect of proxalutamine remains to be verified by clinical trials in other countries because it has not been widely accepted.

Currently, there are more than 30 small molecule candidates in the phase III/IV clinical trial stages that target distinct viral proteins, host cell components, and immunoregulatory pathways. To better guide the development of small molecule drugs, a better understanding of SARS-CoV-2 pathogenesis, viral replication/life cycle and key viral components involved should be obtained. Additionally, further insights into virus-host interactions, or host immune system dysregulation by viruses are needed. Notably, the COVID-19 pandemic has encouraged the discovery of more compounds from natural products, and these attempts have enriched pools of the natural backbones of potential small molecule drugs for beta-CoVs.

## Bunyavirus diseases

### Etiology, epidemiology, and pathogenesis of Bunyavirus diseases

CCHF is caused by Crimean-Congo hemorrhagic fever virus (CCHFV), which is a widespread tick-borne viral zoonotic disease with a human mortality rate ranging from 9% to 50%.^550^ CCHFV is a member of the genus *Nairovirus* in the *Bunyaviridae*. CCHFV has spherical viral particles with a diameter of approximately 80–100 nm, and its spikes consist of glycoproteins GN and GC, which are located on the surface of the cell membrane and are responsible for binding the viral particles to cellular receptors. The virions contain negative-sense small (S), medium (M), and large (L) genomic fragments enveloped by NP and RdRp that initiate transcription and genome replication in host cells.^551^ In addition, secreted glycoprotein 38 (GP38), which corresponds to the maturation of CCHFV particles, could also be a potential of therapeutic target.^552,553^ The first CCHF outbreak occurred in Crimea in 1944.^554^ Subsequently, CCHF disseminated in the Congo, Africa, the Balkans, the Middle East, and Asia.^555^ CCHFV is transmitted mainly through direct contact with infectious blood, tissue and mucous membranes; through the bite of infected Hyalomma species ticks; or through contact with livestock with viremia.^556^ CCHF patients go through four stages of the disease: latent, prehemorrhagic, hemorrhagic, and recovery. The prehemorrhagic phase presents fever, muscle aches, chills, photophobia, headache, and nausea; mild cases recover from this phase, while severe cases progress to a bleeding phase involving petechiae, internal organs, gastrointestinal tract, gums, and nose bleeding; fatal patients suffer from multiorgan failure.^557^ Leucopenia, thrombocytopenia and elevated liver enzymes are hallmarks of this disease. After the virus entry, a series of events, including a decrease in white blood cells, thrombocytopenia, and a decreased hemoglobin levels are induced.^558^ Moreover, coagulation abnormalities, characterized by prolonged prothrombin time (PT) and activated partial thromboplastin time (aPTT), detectable fibrin degradation products, and D-dimers appeared. Elevated levels of aspartate aminotransferase (AST) and alanine aminotransferase (ALT) indicate hepatic injury.^559^ Patients with severe CCHF develop lethal infections, including vascular leakage, multiorgan failure, shock, and hemorrhage. More recently, LDLR has been identified as an entry receptor for CCHFV. However, the pathogenesis of CCHFV is not well understood.^560^

Rift Valley fever virus (RVFV) is a mosquito-borne *phlebovirus* that causes febrile or hemorrhagic illness in ruminants and humans. RVFV is a single-stranded negative-sense RNA virus belonging to the genus *Phlebovirus* of the Bunyaviridae family. RVFV is a quasisymmetrical, icosahedral lattice with a diameter of 100 nm and an envelope decorated with surface glycoproteins.^561^ The glycoprotein precursors Gc and Gn, encoded by the M segment, are the primary targets for viral binding to cellular receptors and cell entry. The interaction of Gn with cell entry factors triggers endocytosis, and the low pH in the endosome enables Gc-mediated fusion of the virus with the membrane.^562,563^ Since its identification in Kenya in 1931, the disease has been repeatedly endemic in eastern and southern Africa and the Arabian Peninsula, causing enormous economic losses to society and posing a serious public health risk.^564^ The most recent RVF outbreak occurred in Kenya in 2020, resulting in 11 deaths. In human infections, the incubation period is approximately 2–6 days, followed by the sudden onset of fever, headache and muscle and joint pain. In human RVF, manifestations usually range from asymptomatic or self-limiting febrile illness; however, liver damage, hemorrhagic disease, encephalitis and neurological disorders are also observed in severe cases.^565^ Most patients recover quickly, but in severe cases, hemorrhagic fever and hepatic injury develop. Newborn lambs and goats are the most susceptible to RVFV, with a mortality rate of up to 90%. Rift Valley fever infection is first transmitted in livestock through mosquito bites but can also be transmitted vertically between animals. Human transmission occurs mainly through direct contact with the blood, excrement, meat, or secretions of infected animals and through the consumption of raw milk.^566^ RVFV infection leads to a remarked inflammatory response, including elevated proinflammatory factor levels, lymphocytic infiltration, neutrophilic infiltration, and a wide range of tissue damage and necrosis.^567,568^ Currently, no preventive or therapeutic approaches have been approved for CCHFV or RVFV. Preclinical attempts are ongoing and some of them have shown efficacy in animal models.

### Animal models for Bunyavirus diseases

The study of CCHF has been hampered by the lack of ideal animal models. Most animal models exhibit viremia but no clinical signs after CCHFV infection. To address this issue, neonatal mice, immunodeficient mice and mouse-adapted models were generated and shown to be susceptible to CCHFV infection (Table 8). IFNAR^−/−^ mice typically develop a rapid-onset fatal disease at 2–5 dpi prior to the development of adaptive immune responses. Additionally, CCHFV caused severe disease in STAT-1^−/−^ mice and in mice lacking both the IFN-I receptor and type II interferon (IFNAGR^−/−^). Additionally, the IFN-I receptor was blocked with the specific antibody MAR1-5A3.^569^ Administration of MAR1-5A3 produced transient IFN-I blockade in mice and resulted in consistent lethal/severe CCHFV infection.^570,571^ This model provides a convenient choice as an IFN-I receptor knockout animal in virtually any wild-type or transgenic mouse without the need for crossbreeding. Compared to genetically KO animals, the antibody-mediated IFN-I blockade model exhibited identical symptoms and mean time to death. A humanized mouse model was also generated by transferring human CD34^+^ stem cells into NOD-SCID-gamma (NSG)-SGM3 mice. These immunodeficient mice lack mature T cells, B cells, and NK cells and exhibit defects in cytokine signaling due to the lack of a common gamma chain. The infection of these mice with CCHFV produced neurological disease, prolonged infection time, and led to death at 13–23 dpi.^572^ A mouse-adapted CCHFV strain (MA-CCHFV) was generated via serial passages of CCHFV-Hoti in Rag2^−/−^ mice (deficient in adaptive immunity, recombination-activating-gene) and C57BL/6J mice. MA-CCHFV causes severe disease in WT mice through viral replication in multiple tissues, liver injury, and severe inflammation. Gender and age-associated disease severity were noted; female mice were largely resistant to severe disease, while young animals progressed to fatal outcomes. Both innate and adaptive immune responses have been proven to be necessary for protection.^573^

**Table 8:** Animal models of CCHFV

Species	Animal model	Pathogens	Max. Dose	Route	Lethality	Signs of disease	Strengths/weaknesses	References
Mice	Neonatal mice	IbAr 10200	50 LD_50_	IP	100%	Viraemia and death	Obvious symptoms/Lacking of full immune function	^911^
	IFNAR^−/−^ C57BL/6 mice	IbAr 10200	10^4^ TCID_50_	IP/IN/IM/SC	100%	Weight loss, ruffled hair, hunchback and lethargy	Highly susceptible to CCHFV and clinical signs similar to human patients/Lacking of important innate immune pathways	^569,570,912^
	STAT-1 knockout mice	IbAr 10200	10^3^ PFU	IP	100%	Lethargy, piloerection, and hunched posture, fever		^913^
	IFNα/β/γR^−/−^ mice	Ank-2 strain	100 TCID_50_	IP	100%	Weight loss, ruffled fur, depression and nasal/ocular discharge		^914^
	SGM3 humanized mice	Oman-199809166	10^4^ TCID_50_	IP	0	Minimal weight loss and recovered	Disease process similar to humans/Time consuming and expensive	^572^
		Turkey-200406546	10^4^ TCID_50_	IP	100%	Weight loss and severe neurological disease		
	Mice adapted, Male mice	Hoti	10^4^ TCID_50_	IP	0–100% (age-dependent)	Weight loss, piloerection, hunched posture, and lethargy	Immunocompetent animal model, gender and age-associated disease severity/Additional mutants introduced by virus adaptation	^573^
Hamsters	STAT-2 knockout hamsters	IbAr10200	10^4^ TCID_50_	IP/IM/SC	100%	Petechial rash, blood coagulation dysfunction, and biochemistry and blood cell count abnormalities	Applicable for pathogenesis study and recapitulated hallmarks of human disease/Immunodeficient and inapplicable for evaluating immune correlates	^915^
Nonhuman primates	Cynomolgus macaques	Afghan09-2990	10^6.2^ PFU	IV	0	Largely uniform and mild disease state	Immunocompetent animal model, similar to human transmission, organ pathology, and disease progression/not consistently lethal, inaccessibility and ethical issues	^576,916^
		Kosova Hoti	10^5^ TCID_50_	IV/SC/SC + IV	0–75%	Thrombocytopenia, hypoproteinemia, edema and epistaxis		

*IP* intraperitoneal, *SC* subcutaneous, *IV* intravenous, *IM* intramuscular, *IN* intranasal, *PFU* plaque-forming units, *TCID*_*50*_ median tissue culture infective dose, *CCHF* Crimean-Congo hemorrhagic fever, *CCHFV* Crimean-Congo hemorrhagic fever virus, – not applicable

Overall, the mouse models above reliably succumb to CCHFV challenge but poorly reflect disease hallmarks in humans. Therefore, a STAT-2 knockout hamster model, which presented as systemic infection and lethal disease, was established. Typical signs of disease, including petechial rash, orbital bleeding, coagulation dysfunction, and abnormal biochemistry and blood parameters, are observed in humans. Among the NHP models used in CCHFV studies, African green monkeys, baboons, and patas monkeys were not susceptible to CCHFV and presented no clinical signs or fulminant disease.^574^ However, CCHFV-infected cynomolgus macaques exhibit piloerection, anorexia, a hunched posture, fever, rashes and orchitis, as well as thrombocytopenia, hypoproteinaemia, edema and epistaxis, manifesting as a clinical shock syndrome accompanied by elevated liver enzymes ALT and AST. In addition, prolonged CCHFV infection in the testes of monkeys was noted. Concurrent latent tuberculosis (TB) in the lungs and liver granulomas were also observed in some CCHFV-infected macaques, which highlights the public health implications of these emerging pathogens in overlapping endemic regions.^575,576^

In BALB/c mice, peripheral infection with RVFV triggers signs of disease, including coat coarsening, lethargy, weight loss and death (Table 9).^577^ These clinical signs appeared within 7 dpi, and some mice even died at 3 dpi.^578^ RVFV-infected mice exhibited acute hepatitis and encephalitis. As in humans, RVFV primarily injures the liver in mice, triggering extensive hepatocellular damage.^577,579^ The condition of the infected mice rapidly deteriorated. Surviving mice exhibited liver injury and encephalitis.^580,581^ Disease in the central nervous system was alleviated in mice that received vaccine prophylaxis and effective drug treatment.^578^ Aerosol infection of BALB/c mice with virulent RVFV ZH501 increased the mean time to death from an average of 3 days to 5 days or even longer.^582,583^ After viral invasion into the brain, mice develop neurological symptoms such as hind limb paralysis or cage circling. Taken together, these findings can be used to investigate the mechanism of RVFV invasion into the nervous system while providing a basis for evaluating the development of vaccines and drugs that prevent RVFV from triggering neurological diseases and encephalitis. In rats, initial RVFV replication occurs in macrophages of the draining lymph nodes, after which the virus is transferred to the liver, where it becomes infected and leads to high viraemia.^584^ After experimental infection with RVFV-ZH501, Wistar-Furth and Brown Norway rats were sensitive, with an LD_50_ of 1–5 pfu given by the SC or aerosol route.^585,586^ Infected rats succumbed at 3–5 dpi due to severe fulminant hepatitis.^587^ Total hepatic necrosis is a typical lesion. Compared to those of Wistar-Furth rats, the mortality of ACI and MAXX rats after IP or SC infection is approximately 50% with high doses of RVFV.^588^ Neurological symptoms and encephalitis were observed. Clinical signs include hind limb paralysis, circling, weakness, head tilt, head tremors, and ataxis.^586^

**Table 9:** Animal models of RVFV

Species	Animal model	Strains	Max. Dose	Route	Lethality	Signs of disease	Strengths/Weaknesses	References
Mice	BALB/c mice	ZH501	10^3^ PFU	SC/IP	100%	Scuffled fur, lethargy, shivers, weight loss, hepatitis/hepatic necrosis, meningoencephalitis	Typical clinical symptoms/No hemorrhagic fever and ocular diseases	^577,578^
		ZH548	2 × 10^4^ PFU	IP	<100%			^579^
Rat	Sprague Dawley rats	ZH501	10^3^ PFU	SC/Aerosol/Conjunctival	70%	Uvea, retina, and optic nerve infection, inflammation in the posterior eye	Mimicking ocular disease, encephalitis, hepatic necrosis and pneumonia in human RVF/-	^917^
	Wistar-Furth	ZH501	10^5.7^ PFU	SC	100%	Hepatic necrosis, pneumonia, lethal encephalitis		^918^
	Lewis	ZH501	10^5.7^ PFU	SC	16%			
	MAXX rats				44%			
Hamster	Syrian hamsters	ZH501	5×10^3^ PFU	IP	100%	Progressive viremia and hepatic necrosis	Highly susceptible to RVFV/Lack of encephalitis	^590^
Ferret	Ferret	ZH501	10^6^ TCID_50_	IN/ID	IN: >70% ID: 0	Mild self-limited febrile illness and later-onset severe encephalitis.	Mimicking RVFV CNS disease in humans/No hepatitis	^564^
Nonhuman primates	Rhesus monkeys	ZH501	10^5^ PFU	IV	18%	Lethal hemorrhagic fever, anorexia, sadness, vomiting, and weakness	Highly susceptible to RVFV/Disease ranged from clinically inapparent to death	^919^
	Marmosets	ZH501	10^7^ PFU	SC/IV/IN/Aerosol	25–100%	Acute hepatitis, delayed encephalitis, and hemorrhagic illness	More closely resembles severe human RVF disease/Symptoms of disease are related to challenge route	^920^
	African green monkeys	ZH501	10^5.86^ PFU	Aerosol	80%	Viral meningitis, formed blood clots, and death	Mimic natural human infections/inaccessibility, ethical issues	^920^
	Cynomolgus macaques	ZH501	10^5.18^ PFU	Aerosol	0/18%	Fever	Insensitive to RVFV	^920^
Ruminants	Sheep	ZH501	–	SC	0–30%	Acute hepatic necrosis, ocular lesions, abortion in pregnant ewes	Natural hosts and susceptible animal, applicable to transmission study/Nonstandardized animal models	^921^
	Calves (<1 month)	ZH501	–	SC	10–70%	Acute hepatic necrosis, meningoencephalitis		^921,922^

*ID* intradermal, *IN* intranasal, *SC* subcutaneous, *IP* intraperitoneal, *IV* intravenous, *CNS* central nervous system, *RVF* rift valley fever, *RVFV* rift valley fever virus, *PFU* plaque formation unit, *TCID*_*50*_ median tissue culture infective dose, – not applicable

Ferrets infected with RVFV-ZH501 exhibited weight loss, hyperpyrexia, lymphopenia, hypoalbuminemia, and CNS diseases such as seizures and ataxia, which are associated with high viral RNA loads in the brain. In addition, mild increases in the AST, ALT, and total protein levels were observed in infected animals.^564,589^ Syrian hamsters are susceptible to RVFV infection, and even with minimal doses of the virus, susceptible animals die within 2-3 days. Niklasson et al. studied the protective effects of immunization in Syrian hamsters following IP infection with 5000 PFU RVFV; actively immunized hamsters and controls died within 4 days of virus attack, whereas passively immunized hamsters died 11 days after virus attack. Deaths are associated with massive hepatic necrosis, whereas late deaths are caused by encephalitis.^590^ The hamster model is applicable for evaluating the diagnosis, transmission, and medical countermeasures used against RVFV.^591^

Ruminants are natural hosts of RVFV and the severity of the disease varies between young and adult animals.^592^ RVFV infection in ruminants leads to abortion and death. Sheep are highly susceptible to RVFV, depending on the virus strain, breed, and age of the animals.^593^ Hepatic lesions developed in neonatal lambs infected with RVFV. Horizontal transmission to noninfected sentinel lambs was also observed. In adult lambs, RVFV infection causes transient pyrexia. Notably, RVFV infection led to uveitis in the Lambs, which progressed to lymphoplasmacytic endotheliitis and anterior uveitis that lasted 1 to 5 days. Hence, lambs may be a good model for studying RVFV-associated ocular pathology in humans.

### Medical countermeasures for Bunyavirus diseases

#### Preventive vaccines for Bunyavirus diseases

Platforms engaged in CCHFV and RVFV vaccine development include inactivated vaccines,^594^ subunit vaccines,^595^ virus-like particle vaccines (VLPs),^596,597^ viral vector vaccines^598–600^ and nucleic acid vaccines^23,57–60^ (Table 10). Compared with tissue-derived vaccines, cell culture-based vaccines are more immunogenic.^601,602^ Several live attenuated RVFV vaccines are used in livestock and have been shown to be immunogenic, but these vaccines are of great safety concern.^603^ Subunit vaccines for RVFV were developed using baculovirus system expressing the Gn ectostructural domain (eGn) and full-length Gc, which protected animals against viral infections when an ISA-25 VG adjuvant was applied.^604,605^ When ectodomains of Gn and Gc were produced using a Drosophila insect cell-based expression system, this subunit vaccine induced high levels of NAbs but showed no protective efficacy in STAT1 knockout Mice. This result suggested that NAbs were not sufficient to confer protection.^595^ This phenomenon was also observed for mRNA, DNA, and VLP vaccines, which suggests that cell-mediated Th1 response and a balanced Th2 response are responsible to the protection.^597,606^ VLPs for RVFV were constructed using the Drosophila Insect protein expression system. When RVFV was used in combination with the Stimune adjuvant, protection against RVFV was achieved in BALB/c mice.^607^

**Table 10:** Representative medical countermeasures for bunyaviruses

Classification	Name	Platform/Strategy	Stage	Efficacy/Benefic	References
Vaccines	Live attenuated vaccine	Live attenuated RVFV	Licensed (Veterinary)	Immunogenic but are of safety concerns	^601,603,923–927^
	Subunit vaccine	Expressing the eGn and full-length Gc of RVFV	Preclinical	Protect sheep	^604,605^
	Viral like particles	Formulate VLPs of RVFV based on Insect Protein Expression System	Preclinical	Complete protection in mice	^607^
	DNA vaccine	Encoding the M-segment GPC gene or expressing GPC and the NP of CCHFV	Preclinical	Immunogenic and protect mice and NHPs	^570,597,609^
		Encoding the Gn and Gc glycoproteins of RVFV	Preclinical		^612^
	mRNA vaccine	Encoding for the CCHFV N or glycoproteins (GcGn)	Preclinical	Complete protection against mice	^606^
	CAdVax-GnGc	Expressing RVFV GnGc based on complex adenovirus-vectored	Preclinical	Immunogenic and protect animals	^611^
	MVA/Adv-N	Deliver the N gene of CCHFV based on MVA or Adv vector	Preclinical	Partially protection in mice	^599,613^
	Replicon RNA	Expressing CCHFV N or GPC	Preclinical	Complete protection in mice	^614^
Antibodies	8A1, 11E7, and 30F7	Mouse antibodies targeting CCHFV Gn/Gc	Preclinical	Protect neonatal mice	^553,623^
	mAb-13G8	Mouse antibodies targeting CCHFV GP38	Preclinical	Protect IFNAR^−/−^ mice	^552,624^
	c13G8	Chimeric human-mouse antibody	Preclinical		
	ADI-37801/ 36121/36145	Human antibodies targeting CCHFV Gc	Preclinical	Complete protection in mice	^626^
	RVFV-268	Human antibodies targeting RVFV Gn	Preclinical	Prevents vertical transmission in rat/sterilizing immunity	^630,633^
	RVFV-268 + RVFV-140	Human antibodies targeting RVFV Gn	Preclinical	Effective at minimal doses and blocking both attachment and fusion	^632^
	R4, R12, R13, R16, R17, R19 and R22	Human antibodies targeting RVFV Gn/Gc	Preclinical	Complete protection in mice	^634^
Small molecular drugs	Ribavirin	Broad spectrum antiviral drug	Preclinical	Controversial efficacy	^616,617,913,915^
	Favipiravir or derivative (H44)	RdRp inhibitor	Preclinical	Reduced mortality rate and decreased viral loads in mice/NHPs	^616,618,619^
	Fluorocytidine and molnupiravir	RdRp inhibitor	Preclinical	Favorable therapeutic effects in vitro	^618,620^

*RVFV* Rift valley virus, *eGn* Gn ectostructural domain, *VLPs* Viral like particles, *GPC* Glycoprotein precursor, *NP* Nucleocapsid protein, *CCHFV* Crimean Congo hemorrhagic fever virus, *Adv* Adenovirus, *MVA* Modified vaccinia virus Ankara, *RdRp* RNA dependent RNA polymerase, *NHPs* nonhuman primates

A DNA vaccine expressing the M-segment glycoprotein precursor gene of CCHFV was developed, termed CCHFV-M10200. In both IFNAR^−/−^ mice and IS mice, M-segment DNA vaccine elicited strong humoral immune responses after three doses of vaccination. Animals were protected from weight loss against CCHFV challenge, with a protective rate of 60%.^570^ Further studies suggested that increasing the dose of CCHFV-M10200 improved the protective efficacy, with 100% protection against homologous CCHFV and 80% protection from heterologous strain. Additionally, they showed that nonstructural M-segment protein GP38 is an important immunogen that plays a role in protect against homologous CCHFV challenge.^608^ Two plasmids DNA vaccine expressing both GPC and NP was also reported. In mice and cynomolgus macaques, the GPC + NP DNA vaccine induced potent antibody and T-cell responses. Vaccinated animals were protected from viraemia and disease.^597,609^ A DNA replicon that encoding the N gene of CCHFV was constructed based on the Sindbis virus vector, and the resulting plasmid was named pSinCCHF-52S. pSinCCHF-52S generated a Th1 response in mice. However, the protective efficacy was not certain.^610^ These DNA vaccines are promising candidates, however, DNA vaccines require the in vivo electroporation-assisted delivery, and at least 3 doses are needed to confer a potent immune response/protection. The CAdVax-GnGc vaccine was found to elicit strong GP-specific IgG antibodies with 100% protection.^611^ A DNA vaccine encoding the Gn and Gc glycoproteins (pWRG7077-RVFV-NSm) was found to be highly immunogenic in BALB/c mice.^612^

In attempts to use the MVA and Adv vectors to deliver the N gene of CCHFV, no protection or partial protection was reported in mice.^599,613^ Alphavirus-based replicon RNA vaccines expressing either CCHFV N or GPC were constructed. Vaccination with RNA expressing the N alone could confer complete protection against clinical disease, and a combination of N and GPC afforded robust protection against disease and viral replication.^614^ Nucleoside-modified mRNA-lipid nanoparticles (mRNA LNPs), encoding CCHFV N or glycoproteins (GcGn) were constructed, both of them induced strong humoral and cellular immune responses and protected 100% of IFNAR^−/−^mice against lethal CCHFV infection, particularly hepatic injury. A comparison of the immune responses induced by CCHFV Gc and Gn antigens revealed that Gc protein was more immunogenic than Gn protein. Overall, genetic immunization is an attractive approach for CCHFV. Compared to DNA vaccines, mRNA-LNP vaccines combine the ability to induce an effective immune response, the safety of transient carriers, and the flexibility of genetic vaccines. These results support the development of a mRNA-LNP based vaccine against CCHFV.^606^

#### Therapies for bunyavirus

Ribavirin is an antiviral drug that has been used in patients with CCHF.^615^ However, the efficacy of ribavirin in animal models is controversial; it protected STAT^−/−^ mice and STAT2^−/−^ hamsters but failed to protect IFNAR^−/−^ mice.^616,617^ Compared with ribavirin, favipiravir or its derivative (H44) was protective in IFNAR^−/−^ mice, as indicated by a reduced mortality rate and decreased viral load.^616,618^ Similarly, favipiravir attenuated viremia and the viral load in tissues in an NHP model.^619^ Furthermore, the therapeutic efficacy of favipiravir in patients needs to be investigated. 2′-Deoxy-2′-fluorocytidine and molnupiravir showed favorable therapeutic effects in vitro, suggesting that these may be effective antiviral agents.^618,620^ In severe cases, patients experience dysregulation of the inflammatory response and cytokine storms; therefore, corticosteroids can be used at this stage.^558^

In the early stages, CPT is used for the treatment of CCHF patients.^621,622^ Although several successful cases have been reported, only a limited number of people have been treated with convalescent serum; thus substantial therapeutic efficacy is difficult to document. Both pre-GN and GC-specific mouse mAbs have been reported.^623^ Among them, GC-targeting mAbs have shown cross-neutralizing activity against multiple strains.^553^ Both nonneutralizing and neutralizing mAbs protect neonatal mice. Interesting, only nonneutralizing pre-GN(GP38)-specific mAbs, known as mAb-13G8, efficiently protected IFNAR^−/−^ mice, and IFN-I activity blocked mice.^624^ These findings suggested that GP38 is a potential target for CCHFV immunotherapy and demonstrated the role of ADCC and complement-mediated functions in protection against CCHF.^625^ However, this protection seems to be strain-limited, as passive protection by mAb-13G8 against heterologous CCHFV strains is poor.

Furthermore, several human anti-GP38 mAbs were isolated from a CCHFV survivor.^552^ These mAbs bind to other antigenic sites on GP38 compared to mAb-13G8. Using fluorescence-activated cell sorting, a panel of 361 rGn/Gc-reactive mAbs bound to six distinct antigenic sites in the Gc subunit. Neutralizing ability was associated with binding sites, of which fusion-loop targeting (Site 1) and domain II-targeting (Site 3) nAbs displayed the highest median potencies of approximately 70–80% against CCHFV clade IV.^626^ Pairwise screening revealed that combinations of the non-competing nAbs, ADI-37801 and ADI-36121/ADI-36145, afforded synergistic neutralization. By linking variable domains of synergistic nAbs, a bispecific antibody (bsAb) was obtained, which exhibited enhanced neutralization breadth and potency and in vivo antiviral potency. Notably, a single therapeutic dose of bsAb provided protection even when administered 24 h after the challenge. Subsequently, structural basis of pre-fusion Gc bound to the above synergistic neutralizing antibodies was resolved.^627^ ADI-36121 targets fusion loops while ADI-37801 binds to the host-membrane insertion surface (HMIS) and blocks Gc trimer formation. Together, they blocked membrane fusion. Structural information revealed the neutralization mechanism of anti-CCHFV antibodies and provided the molecular basis for developing CCHFV-specific medical countermeasures.

Allen et al. revealed a molecular basis for antibody-mediated neutralization of RVFV. The authors characterized a distinct region, the membrane-distal head domain of RVFV Gn, as a key site of vulnerability, and identified a class of rabbit monoclonal antibodies that protect RVFV in an animal model.^628^ These antibodies are predicted to prevent exposure to viral fusion loops and have shown protective efficacy in a murine model of RVFV infection. Based on single memory B-cell screening, two Gn-specific NAbs were isolated from a rhesus monkey immunized with recombinant human adenovirus type 4 expressing RVFV Gn and Gc.^629^ Both NAbs protected host cells from RVFV infection. According to docking models, these two NAbs might preclude RVFV glycoprotein rearrangement, thus hindering the exposure of fusion loops in Gc to endosomal membranes during virus invasion. A previous study reported the generation of glycoprotein Gn-specific neutralizing antibodies in individuals naturally infected with RVFV in Kenya.^630^ One typical mAb, RVFV-268, displayed excellent neutralization of RVFV and was mapped to the surface exposed residues of domain A on the Gn surface. After intraperitoneal injection, RVFV-268 rapidly reaches placental and fetal tissue, and prevents maternal and fetal infection in a dose-dependent manner. In vitro, RVFV-268 reduced viral replication in placenta explant cultures, and in vivo, RVFV-268 prevented vertical transmission in a rat model of congenital RVF. Passive transfer of the RVFV-268 mAb from parent to fetus 2 h prior to RVFV challenge or 24 h post challenge protected dams and offspring from RVFV infection. These results support the use of RVFV-268 mAb as a prophylaxis and post infection therapy to prevent RVFV infection and vertical transmission, thus protecting mothers and offspring.

In another study, two synergistic monoclonal antibodies targeting the glycoprotein Gn were identified.^631^ In therapeutic attempts, the neutralizing mAb Gn3 alone showed a moderate efficacy of approximately 58.3% when given pre or postchallenge. In contrast, combination therapy with nonneutralizing mAb Gn32 conferred complete protection when applied 30 min after the lethal challenge dose. The enhanced protective efficacy is probably attributed to cooperative neutralization effects, which warrants further study. More recently, a combination of dual-mechanism human monoclonal antibodies conferred protection against RVFV at low doses.^632^ Structural analysis and characterization revealed a prototypical potent Gn domain-A binding antibody that blocks attachment and an antibody that inhibits infection by abrogating the fusion process. Interestingly, according to a competitive assay, the Gn domain-A antibody does not directly block the receptor-ligand interaction of RVFV Gn. This study provides insights into combination therapy using representative mAbs from distinct classes of neutralizing mechanisms that act via both attachment and fusion to accomplish protection in low doses of this antibody.

Chapman, et al. isolated a total of 20 monoclonal antibodies (mAbs), including Gn-specific mAbs, hetero-oligomer glycoprotein complex (Gc+Gn)-specific mAbs and Gc-specific mAbs from human B cells.^633^ Gc-specific mAbs exhibited relatively lower neutralizing capacity than their counterparts. mAbs that bind to coexpressed full-length Gc-Gn proteins and Gn domain A-specific mAbs can inhibit the exposure of the fusion loop in Gc, thus inhibiting RVFV fusion to cells. Competition binding analysis with coexpressed Gc/Gn and mutagenesis library screening indicated that four competition groups for binding to viral antigen on the RVFV surface were recognized by neutralizing antibodies, with two sites of vulnerability for neutralization on Gn. Since mAbs targeting domain A caused partial inhibition of fusion while Gc+Gn-specific mAbs caused complete inhibition of fusion, a representative mAb, RVFV-268 was tested in a mouse model. RVFV-268 recognized three of the antigenic sites and reduced the rate of lethal hepatic disease in adult mice when it was prophylactically delivered or treated 2 or 4 days after infection. Sterilizing immunity against RVFV infection was achieved when the mAb was delivered 2 h prior to inoculation. Similarly, Wang et al. reported the isolation of monoclonal antibodies from antigen-specific single memory B cells in a convalescent patient.^634^ Among these antibodies targeting the RVFV envelope proteins Gn or Gc, Gn-specific monoclonal antibodies exhibited much greater in vitro neutralizing activity and protective efficacy in mice against RVFV infection. In cell interactions, they provided evidence that Gn monoclonal antibodies interfere with soluble Gn binding to cells, thus blocking the binding of Gn on virions to susceptible cells. Structural analysis of Gn complexed with Gn-specific monoclonal antibodies revealed two potential neutralizing hotspots on Gn domain I. These results highlight the potential of human antibody-based therapeutics and provide a structure-based immune-determining region against RVFV.

Interestingly, there is also evidence that isotype-specific Fc effectors enhance antibody-mediated protection in vivo.^635^ In a proof-of-concept study, compared with IgG1 mAbs, IgG2a mAbs had an increased capacity to induce effector functions and conferred better protection against RVFV challenge in a lethal mouse model. Overall, this study showed that Fc mediated functions are a critical component of humoral protection from RVFV.

An equine immunoglobulin F(ab’)_2_ fragment demonstrated an in vitro neutralization effect and reduced mortality in infected mice.^636^ Another study revealed that llama-derived single-domain antibody (VHH) building blocks were assembled into highly efficient neutralizing complexes using bacterial superglue technology, and this multimeric complex showed protective effects in mice.^637^

## Nipah virus disease

### Etiology, epidemiology, and pathogenesis of Nipah virus disease

Nipah virus (NiV) is a viral zoonotic pathogen that was first reported in Singapore and Malaysia in 1998.^638^ NiV is a member of the genus *Henipavirus*, family *Paramyxoviridae*, it is a negative-sense, single-strand, nonsegmented, enveloped RNA virus possessing helical symmetry.^10^ The fusion glycoprotein (F) and the attachment glycoprotein (G) of the genome are responsible for the cellular attachment of virus particles and subsequent entry into the host cell.^639,640^ Several species of fruit bats are natural hosts of NiV, while pigs are intermediate hosts of NiV.^641,642^ NiV is transmitted to humans by direct contact or through contaminated food.^643^ In humans, NiV infection results in severe and often fatal respiratory and neurological manifestations, presented as interstitial pneumonia, systemic vasculitis, and meningitis.^644^ A human mortality rate of Nipah virus disease was up to 75% have been reported.^645^ The outbreak of NiV has been reported in South and Southeast Asia, including Bangladesh, Cambodia, Timor-Leste, Indonesia, India, Malaysia, Papua New Guinea, Vietnam and Thailand, and poses a potential threat to global health security.^646^ Sporadic NiV outbreaks, human-to-human transmission and zoonotic transmission have been associated with hundreds of deaths over the past decades, posing a significant threat to domestic animals and humans. At disease onset, NiV is first detected in bronchial epithelial cells. Respiratory epithelial cells are also infected thereby triggering dysregulation of the levels of inflammatory factors, leading to the recruitment of immune cells and ultimately resulting in the development of acute respiratory distress syndrome (ARDS)-like disease.^647^ Later in the disease, the virus spreads from respiratory epithelial cells to lung endothelial cells. Subsequently, the virus enters the blood and spreads to the target organ either by free transmission or by binding to host leukocytes, leading to multiorgan failure.^648^ Viruses can enter the central nervous system through two routes: the hematogenous route and/or the olfactory route.^649^ Symptoms of NiV disease include fever, difficulty breathing, cough and headache. Encephalitis and seizures are associated complications.^650^

### Animal models for Nipah virus disease

Animal models for Nipah virus disease have been established in mice, hamsters, guinea pigs, ferrets, pigs, dogs, cats and NHPs. (Table 11) Wild-type mice were insensitive to NiV infection by IN or IP inoculation, whereas mice intracerebrally injected with 10^5^ PFU of NiV died within 6 days.^651^ In contrast, the pathogenesis of NiV was studied in IFNAR-KO mice, which are highly susceptible to NiV infection regardless of the route of administration.^651^ Intraperitoneally infected IFNAR-KO mice exhibited weight loss, agitation, impatience, dyskinesia, head tilt, and paralysis. Moreover, IFNAR-KO mice developed vasculitis, meningitis, and bronchial interstitial pneumonia. NiV was detected in multiple organs, including the brain, lungs, spleen, and liver, with the highest titers detected in the brain and lungs.^651^ Consequently, IFNAR-KO mice are a suitable small animal model for treating NiV. Other innate signaling immunodeficient mice were also used in pathogenesis studies, which highlighted the role of pathogen recognition in sensing NiV infection and protection. Specifically, the host adapter mitochondrial antiviral signaling protein (MAVS) and myeloid differentiation primary response 88 (MyD88) mediate the control of NiV infection. Moreover, the cGAS/STING pathway plays synergistic roles in host protection.^652,653^ To investigate the molecular mechanisms of NiV-associated ALI in humans, a human lung xenograft humanized mouse model was developed.^654^ NiV replicated efficiently in transplanted mice, targeting both the endothelium and epithelium and triggering inflammatory cytokines and chemokines in lung tissue. Unfortunately, this model did not present obvious signs of disease.

**Table 11:** Animal models of NiV

Animal species	Animal model	Strains	Max. Dose	Route	Lethality	Signs of disease	Strengths/Weaknesses	References
Mouse	C57BL/6 mice	UMMC1	10^5^ PFU	IP	0	No	Challenge route dependent lethality/Inability to mimic natural infection routes	^651^
				IC	100%	–		
	IFNAR-KO mice		10^6^ PFU	IP	100%	Weight loss, agitation, impatience, dyskinesia, head tilt, paralysis, vasculitis, meningitis, bronchial interstitial pneumonia	Lethal encephalitis similarly to human infections/Inability to mimic natural infection routes	
				IC	100%			
				IN	60%	Neuronal signs, breath difficulties		
	MyD88/TRIF/MAVS/STING KO mice	Malaysia	10^6^ PFU	IP	100%	Posture, prostration, and paralysis	Applicable to the study of pathogenesis and immune correlates/In applicable to extensive usage	^652^
	MyD88/MAVS-KO mice				66%	Posture, prostration, and paralysis		^653^
	Human Lung Xenograft	Malaysia	10^5^ TCID_50_	Intragraft injection	0	No	Facilitate study of pathogenesis and virus-host interactions/No obvious signs of disease	^654^
Hamster	Syrian hamsters	Malaysia/Bangladesh	10^7^ PFU	IP/IN	100%	Hyperreflexia, ataxia, irregular breathing	Suitable animal model for acute NiV infection/Differential time to death in different challenge routes and no parenchymal fibrinoid necrosis in the lungs	^910^
		Malaysia	6 × 10^5^ PFU	IN	100%	Imbalance, paralysis of limbs, lethargy, muscle twitching and respiratory distress		
		Cambodian	13500 PFU	SC	100%	Paralysis, limb tremors, respiratory distress, unsteady gait; weight loss, hypothermia		^660^
Guinea pig	Guinea pig	Malaysia	10^7^ PFU	IP	0	Brief fever and weight loss but recovered.	Reproductive infection model/Variable clinical symptoms between different NiV isolates and routes	^659^
			6 × 10^4^ PFU	IP	100%	Vascular necrosis and inflammation, bladder and female reproductive tract infections evident		^660^
Ferret	Ferret	Malaysia	5 × 10^4^ TCID_50_	IN	100%	Fever, depression, lethargy, loss of appetite, difficulty breathing	Ability to exhibit complex behaviors for clinical assessment/-	^662,674,681^
Pig	Pig	Malaysia	5 × 10^3^ TCID_50_	SC	66%	Fever, unconsciousness, coughing, and ataxia	Marked respiratory and neurological features, age-associate lethality/Ununiform lethality	^663,928^
				IN/OR	0–31%	No		
Dog	Dog	Malaysia	5 × 10^3^ TCID_50_	SC/OR	0–100%	Fever, respiratory distress, nasal discharge and conjunctivitis	Similar pathological changes to those in human patients/In applicable to study dissemination mechanisms	^664^
Cat	Cat	Malaysia			50–100%	Fever, increased respiratory rate, inappetence, depression	High susceptibility to NiV/In applicable to study dissemination mechanisms	^665,666,929^
Nonhuman primates	African green monkey	Malaysia	1.3 × 10^6^ PFU	IT/IN/Aerosol	100%	Fever, anorexia, respiratory disease, lethargy, rash, depression, behavioral changes, vasculitis, hemorrhage, edema, thrombocytopenia, meningitis	Mimic serious clinical diseases in humans/inaccessibility and ethical issues	^667,668,930^
		Bangladesh	5 × 10^5^ PFU	IT/IN/Aerosol	100%			^668,669,671^
	Squirrel Monkey	Malaysia	10^7^ PFU	IN	0	Anorexia, weight loss, depression, respiratory disease, uncoordinated motor movements, acute neurologic illness		^673^
				IV	75%			
	Cynomolgus Monkeys	Bangladesh/Malaysia	5× 10^5^ PFU	IT/IN	0	Self-limiting/asymptomatic	Study of the immune response and organism after infection/mild or asymptomatic infection	^672^

*IN* intranasal, *IP* intraperitoneal, *IC* intracerebral, *SC* subcutaneous, *IT* intratracheal, *IV* intravenous, *NiV* Nipah virus, *PFUs* plaque-forming units, *TCID*_*50*_ median tissue culture infective dose, *MyD88* myeloid differentiation primary response 88 (MyD88), *TRIF* TIR-domain-containing adapter-inducing interferon-b, *MAVS* mitochondrial antiviral-signaling protein, *STING* stimulator of interferon genes, – not applicable

The Syrian hamster is another small animal model for treating NiV. After NiV infection, the Syrian hamsters exhibited pathological changes and died of acute encephalitis.^655,656^ IP infection in hamsters was associated with the rapid onset of symptoms within a day, and the animals died at 5–9 dpi.^657^ In contrast, hamsters infected by the IN route showed progressive deterioration of disease; animals developed disequilibrium, limb paralysis, lethargy, muscle twitching, and respiratory distress; and died in 9–15 days.^657,658^ Compared to other organs, the brain is the most severely affected organ in terms of vascular and parenchymal lesions. In addition, NiV-infected hamsters develop myocardial infarcts similar to those observed in humans.^659^ Animals infected with high doses of NiV died of acute episodes of severe respiratory distress.^660^ In contrast, animals infected with low doses of NiV developed respiratory disease and neurological signs.^647,661^ In guinea pigs, intraperitoneal inoculation of 6 × 10^4^ PFU of NiV resulted in a mortality rate of 92.86%, the majority of which died at 4–8 dpi.^660^ The spleen, lymph nodes, bladder, ovaries, uterus, and brain were the most extensively infected organs. Pathology revealed mild to severe histiocytosis, lymphocytic meningitis, meningeal vasculitis, and lymphohistiocytic meningoencephalitis. NiV-infected ferrets presented fever, depression, lethargy, loss of appetite, and labored breathing and eventually succumbed to infection. The gross pathology results included thrombocytopenia, lymphopenia, rash, severe congestion and hemorrhage in the lungs, and liver and spleen damage.^662^

Pigs are natural hosts of NiV. NiV-infected pigs exhibit acute and self-limiting clinical disease, including barking cough and, occasionally, muscle spasms, myoclonus, trembling, fever, and respiratory and neurological features, and typically do not result in fatal outcomes.^641^ The lethality of NiV infection in pigs was challenge route-related, SC challenge resulted in 66% mortality, while oral and IN infection caused asymptomatic or mild infections. Young pigs were relatively more susceptible to NiV.^663^ Overall, NiV induced respiratory and neurological syndrome consistent with that observed in Malaysian pigs.

Domestic dogs and cats were also included as models of NiV infection. In dogs, NiV infection results in respiratory distress, fever, nasal discharge and conjunctivitis. Pathology revealed findings resembling those of infected humans, including heavy lungs with visible congestion, mottling, and consolidation of all lobes in addition to reddening of the renal capsules and cortices.^664^ Experimentally, it has been demonstrated that NiV is not transmitted between dogs.^664^ For domestic cats, NiV infection via SC challenge resulted in 100% lethality, while NiV infection via nasal challenge caused 50% lethality. Clinical symptoms included fever, tachypnea, anorexia, and depression. Histopathological lesions in the lungs included bronchitis and alveolar hemorrhage, as did meningitis. Horizontal transmission of NiV between cats has not been recorded, while vertical transmission of NiV has been noted in pregnant cats.^665,666^

NiV-infected African green monkeys developed neurological disease, severe respiratory lesions, and systemic vasculitis.^667–669^ A uniform severe ARDS-like disease developed.^670^ To mimic the natural infection route, African green monkeys were infected with NiV-Bangladesh via the IN route, which resulted in uniform lethality.^668^ Under the same conditions, NiV-Bangladesh was more pathogenic than was NiV-Malaysia in the AGM.^671^ In contrast to African green monkeys, Cynomolgus monkeys were asymptomatic or had mild symptoms of NiV infection.^672^ Similarly, squirrel monkeys intravenously infected with NiV developed acute neurologic and respiratory illnesses, whereas monkeys infected intranasally exhibited milder clinical signs and recovered from the disease at 3–7 dpi.^673^

### Medical countermeasures for Nipah virus disease

Several vaccine candidates for Nipah virus disease have been tested in preclinical studies (Table 12). A VSV based vaccine protected nonhuman primates and hamsters against NiV infection.^674^ Another recombinant vaccine was produced using the vaccinia virus strain LC16m8 expressing the NiV glycoprotein (G) or fusion protein (F). It was also demonstrated that this recombinant vaccine protects hamsters from lethal NiV infection and generates high levels of neutralizing antibodies in hamsters.^675^ One VLP vaccine consisting of three NiV proteins, G, F and M, was shown to induce a neutralizing antibody response in mice.^676^ In addition, an APC-targeting vaccine has also been developed by fusing humanized anti-CD40 mAbs with conserved peptides. It showed positive immunogenicity, cross-neutralization of NiV and Hendra virus (HeV), and promising protection in an African green monkey model.^677^ A novel approach is to add a cholesterol moiety to the C-terminal heptapeptide repeat sequence (HRC) of F proteins to facilitate membrane targeting and fusion of the peptide, increasing the antiviral effect.^678^ Favipiravir has been shown to be effective in treating Nipah virus in hamster models.^679^ The National Institute of Allergy and Infectious Diseases launched an early clinical trial to evaluate the mRNA-1215 Nipah virus vaccine produced and developed by Moderna Corporation, which has entered phase I clinical trials. The VSV-NIVG vaccine developed by public health vaccines has also entered Phase I clinical trials. The vaccine is a multivalent vaccine that expresses glycoproteins of Nipah virus and Ebola virus. Phylex BioSciences announced the second-generation nanoparticle mRNA vaccine technology will be directly used in the development of Nipah virus vaccines. The expected advantages of nanoparticle vaccines are excellent immune responses, long-term protection against cell-mediated immunity, and prevention of virus transmission in the brain.^680^

**Table 12:** Representative medical countermeasures for Nipah

Classification	Name	Platform/Strategy	Stage	Efficacy/Benefic	References
Vaccines	VSV-F/G	Vesicular stomatitis virus vectors expressing NiV F or/and G	Clinical	Immunogenic and protected ferrets	^674,680^
	LC16m8-F/G	Vaccinia virus LC16m8 expressing NiV F/G	Preclinical	Protects hamsters	^675^
	Viral like particles	Formulate VLPs of NIV by expressing G, F and M protein	Preclinical	Immunogenic in mice	^676^
	CD40 vaccine	Fusing a humanized anti-CD40 monoclonal antibody with conserved peptides	Preclinical	Protect African green monkey	^677^
	mRNA-1215	/	Clinical	/	/
Antibodies	m102.4	Human monoclonal antibody targeting G	Clinical	Effective in NHPs and ferrets	^681–683^
	Hu1F5	Human monoclonal antibody targeting F	Preclinical	Effective in NHPs	^931^
	NiV41	Human monoclonal antibody targeting F	Preclinical	Cross-reactive to NiV and Hendra virus and protect hamsters	^684^
Small molecular drugs	Fapiravir	RdRp inhibitor	Preclinical	Effective in hamsters	^679^
	Ribavirin	Broad spectrum antiviral drug	Preclinical	Used in clinical in Kerala in South India	^649^

*NiV* Nipah virus, *G* glycoprotein, *F* fusion protein, *M* matrix protein, *VLPs* viral like particles, *RdRp* RNA dependent RNA polymerase, *NHPs* nonhuman primates

No specific drug is currently approved for the treatment of NiV disease. However, ribavirin was used as an anti-Niv drug in Kerala, South India.^649^ Several mAb therapies have been evaluated in animal models. A fully humanized mAb targeting the viral G protein, m102.4, has shown protective effects in ferret model of Niv disease.^681^ Furthermore, m102.4 was also evaluated in a nonhuman primate model and showed positive therapeutic effects.^682^ Recent studies have shown that a mAb against the prefusion conformation of the F glycoprotein protects AGMs from severe NiV attacks more effectively than m102.4.{Zeitlin, 2024 #6503} In Phase I clinical trial, m102.4 was tolerated and safe.^683^ Based on a naïve human phage-displayed Fab library, two neutralizing antibodies targeting G proteins, NiV41 and NiV42, were screened. One of them, NiV41, is cross-reactive to NiV and HeV and protects hamsters from lethal NiV infection.^684^ Existing treatments are targeted at the early stages of viral infection, including the use of neutralizing virus-specific antibodies and blocking the fusion of membranes with peptides binding the fusion protein of the virus.^685^

## Zika virus disease

### Etiology, epidemiology, and pathogenesis of Zika virus disease

Zika virus (ZIKV) is the causative agent of Zika virus disease and is a mosquito-transmitted RNA virus with an 11 kb single-stranded genome.^686^ The viral genomic RNA is composed of a single open reading frame that encodes a long polyprotein that is cleaved by host and viral proteases into 10 proteins. These proteins included three structural proteins, capsid (C), premembrane/membrane (pr-M/M), and envelope (E), as well as seven nonstructural proteins (NS1, NS2A, NS2B, NS3, NS4A NS4B, and NS5).^687^ Structural proteins play a role in the viral entry to target cells, virus assembly, and virion secretion.^688^ Nonstructural proteins play a crucial role in the replication of viral RNA, the assembly and release of virions, and the evasion of innate immunity.^689,690^ Previous studies have shown that ZIKV NS4A and NS4B induce autophagy by suppressing multiple target proteins in signaling pathways including the PI3K-AKT pathway. However, the inhibition of ZIKV in this pathway could result in defective neurogenesis.^691,692^ Furthermore, ZIKV NS1, NS2A, NS2B, NS4A, NS4B, and NS5 can target important factors to interfere with signaling pathways and IFN induction to evade the innate immune response.^693–695^ Moreover, NS1 contributes to the pathophysiology of disease by causing vascular leakage and endothelial cell injury, as well as by generating autoimmune reactions.^696^ It also functions as an immune evasion factor by delaying complement-dependent lysis of infected cells.^697^ Researchers have shown that the ubiquitin E3 ligase HRD1 can directly interact with NS4A and ubiquitinate a conserved lysine residue for ER-associated degradation to prevent the excessive accumulation of NS4A and balance the homeostasis of viral proteins.^698^

In 1969–1983, ZIKV was detected in India, Indonesia, Malaysia, and Pakistan. The virus also caused epidemics in French Polynesia, Easter Island, the Cook Islands, and New Caledonia in 2013–2014.^699^ The ZIKV outbreak in French Polynesia caused more than 30,000 cases. An outbreak caused by infection with African strain of Zika virus in Cape Verde in 2015–2016 involved thousands of cases. In the following year, mosquito-borne ZIKV transmission was documented in 84 countries or territories worldwide, including 61 countries or territories where ZIKV was firstly introduced in 2015.^700^ The WHO has declared it a global health emergency.^701^ Zika can be transmitted through mosquito bites, blood transfusion, sex, and from pregnant woman to fetus.^702,703^ Zika virus is transmitted to humans by the bite of an infected mosquito from the Aedes genus, primarily *Aedes aegypti*, in tropical climates. This mosquito spreads dengue, chikungunya, and yellow fever. The majority of Zika virus infections result in either no symptoms at all or very minor symptoms. The most typical Zika symptoms include fever, rash, headache, joint pain, red eyes, and muscle pain, which can last for several days to a week. However, pregnancy-related ZIKV infection can have negative effects, including fetal death, congenital microcephaly, or other severe brain abnormalities.^704^ In addition, ZIKV infection has also been associated with Guillain-Barré syndrome (GBS), encephalopathy, meningoencephalitis, myelitis, uveitis, paresthesia, and severe thrombocytopenia.^705^ Currently, no vaccines or drugs are approved for treating Zika virus infection.

### Animal models of Zika virus disease

#### Mice

Wild-type mice are insensitive to ZIKV (Table 13).^706,707^ ZIKV can antagonize the type I interferon response; more precisely, the NS5 protein of ZIKV promotes the degradation of STAT2, a transcription factor that mediates signaling through the type I interferon receptor IFNAR.^708,709^ However, NS5 was unable to promote the degradation of STAT2 in mice, which may explain the insusceptibility of mice to ZIKV.^708^

**Table 13:** Animal models of Zika virus infection

Animal Species	Animal model	Pathogens	Max. Dose	Route	Lethality	Signs of Disease	Strengths/Weaknesses	References
Mice	Neonatal mice	ZIKV	–	IC or SC	–	Lethargy, ataxia, and paralysis	Neurological manifestations/Pathological changes confined to nervous system	^710,711^
		PRVABC59	2 × 10^3^ PFU	SC	20%	Ataxia, and seizures		
	IFN-α/βR^−/−^ mice	MP1751	10^6^ PFU	SC	100%	Weight loss, elevated temperature	Systemic symptoms/No reproductive lesions	^713^
		ZIKV	10^3^ FFU	Retro-orbital	–	Ruffled fur, and lethargy	Obvious neurological manifestations/Lack of typical clinical features	^714^
		DAK AR D41525	6.4 log_10_ PFU	SC	40%	Weight loss, and severe neuropathologic changes	Obvious brain lesions/Need IFN I blockade	^715^
				IP	100%			
		ZIKV	10^5^ PFU	FP/IP	100%	Weight loss, lethargy, hunched posture	Brain pathology/Lack reproductive symptoms	^716^
		H/PF/2013	10^3^ FFU	SC	–	Fetal demise, IUGR, and microcephaly,	In utero transmission/No neurological signs in survived animals	^722^
	BALB/c mice	PRVABC59	6×10^6^ TCID_50_	IP	66.7/100%	Weight loss, dyspnea, lethargy, and ruffled fur	Immunosuppressed mice for studying long-term infection/Need dexamethasone for immunosuppression	^719^
	C57BL/6 mice	H/PF/2013	10^3^ FFU	SC	–	No	Obvious male reproductive tissue injury/Antibody treatment needed	^717^
		ma Dakar 41519	10^6^ FFU	SC	–			
		H/PF/2013	10^3^ FFU	SC	–	IUGR	In utero transmission model/Antibody blockade of Ifnar1 signaling	^722^
		SZ01	3×10^5^ PFU	IP	–	Fetal mice exhibited a reduced cavity of lateral ventricles and a discernable decrease in surface areas of the cortex	Vertical transmission model/Ventricular changes are contrary to that in human disease	^932^
	SJL mice	ZIKV	2×10^2^/8×10^9^/2×10^11^ PFU	IV	–	Weight loss, whole-body growth delay or IUGR	Presenting brain malformations/-	^723^
Syrian golden hamsters	–	ZIKV	500 TCID_50_	SC	37%	Weight loss	Vertical transmission/Lack of typical clinical features	^724^
			50 TCID_50_		42%			
		ArD 41525	5.4 log_10_ PFU	SC	–	Lethargy	-/Mild signs of disease	^725^
				IP				
Guinea pigs	–	ZIKV	10^5^/10^6^ PFU	SC or IN	–	Fever, lethargy, hunched posture, ruffled fur, and decrease in mobility	Contact transmission model, Viral replication in brain/No observed signs of disease	^726^
								^728^
Ferrets	Ferrets	PRVABC59	10^6^ TCID_50_	SC	28%	Fever, brains deformities, and hypertrophied vessels.	Neurological symptoms/Presenting inconsistent symptoms	^731^
NHPs	Rhesus monkey	Rio U-1/2016	10^4^ PFU	SC	–	Fetal loss and demise	-/Inconsistent signs of disease	^933^
		PRABC59	10^5^ FFU	SC	–	Decreased fetal- placental weight ratio, malaise, ataxia, and deficits in visual, cardiomyopathy, seizure activity	Uteroplacental pathology/No obvious fetal signs of disease	^934^
								^746^
								^742,748^
		ZIKV	300 TCID_50_	IR	–	Lymphoid- and neuro-tropism and histopathologic abnormalities	High incidence (78%) of spontaneous pregnancy loss/Lack of other typical symptoms	^739^
		DAK	10^8^ PFU	SC	–	Fetal loss	Vertical transmission/Dose dependent	^740^
		Brazil	10^3^ PFU	SC	–	Fetal loss, smaller brain size, and histopathologic brain lesions	Presenting the hallmarks including vascular compromise and neuroprogenitor cell dysfunction.	^743^
		Brazil	5.0 log_10_ PFU	IV and IA	–	Fetal death	Developing gross neuroanatomical abnormalities/No gross microcephaly	^744,747^
	Cynomolgus macaque	ZIKV PRVABC59	2×10^6^ FFU	IV	–	Marginal change in blood cells, including PLT, RBC, and WBC counts	Intravaginal inoculation model/No detectable viremia or clinical symptoms	^759^
		BeH815744/PF13/251013–18/ZKA-16-291	5×10^6^ PFU	IV/SC	–	Fever/Weight loss	Systemic inflammation/Lack of neurological symptoms	^754^
	African green monkey	ArD 41525	4.5 log10 PFU	SC, IV, or IA	–	Lymphadenopathy	Sexual transmission model/No clinical symptoms	^760^
	Marmoset monkey	MR766	10^4^ PFU	IM	–	Drowsiness, and weight loss	Viral transmission by the oral route/Lack of apparent clinical symptoms	^764^
		SPH2015	2.5×10^5^ PFU	IM	–	Spontaneous pregnancy loss, and fetal neurocellular disorganization	Vertical transmission/Lack of apparent clinical symptoms	^767^
	Baboon	ZIKV	10^6^ PFU	SC	–	Rash, conjunctivitis	Similar to human ZIKV infection/No obvious neurological symptoms	^769^
		French	10^4^ FFU	SC	–	Fetal death, weight loss, rash and conjunctivitis	Vertical transmission model/No gross microcephaly	^770,772^
			10^6^ FFU	SC	–			
		ZIKV	10^7^ PFU	SC	–	Intermittent rectal bleeding, rash	Substantial injury to fetal brain/Subtle injury pattern without microcephaly	^762^
		FSS13025	10 PFU	SC	–	Abnormal fetal autopsy	–	^763^

*ZIKV* Zika virus, *SC* subcutaneous, *IC* intracutaneous, *PFU* plaque-forming unit, *FFU* focus-forming unit, *TCID*_*50*_ 50% tissue culture infective dose, *IP* intraperitoneal, *FP* foot pad, *IUGR* intrauterine growth restriction, *IV* intravenous, *IN* intranasal, *IR* intrarectal, *IA* intraarterial, *IM* intramuscular, – not applicable

##### Neonatal mice

Neonatal mice are susceptible to ZIKV due to their immature immune system. ZIKV infection in 7- to 8-day-old mice damages the central nervous system and triggers tremors, ataxia, and epilepsy, ultimately leading to lethal infection.^706,710,711^ In a proof-of-concept study, ZIKV infections were induced in four different species of mice.^712^ The results showed that neonatal Kunming, ICR and C57BL/6 mice were fatally susceptible to ZIKV infection while BALB/c mice were resistant to ZIKV infection. In C57BL/6 neonates, a dose-dependent viral infection pattern was observed. In these susceptible mouse species, microcephaly and neuronal symptoms were observed. According to pathologic and immunofluorescent results, ZIKV infected different areas of the CNS and replicated in multiple organs, including the liver and testis. Thus, ZIKV infection in newborn mice can be used to determine pathogenesis and assess long-term neurodevelopmental factors and behavioral sequelae associated with ZIKV infection during brain maturation.

##### Immunodeficient mice

Mice genetically deficient in the type I interferon signaling pathway exhibited increased susceptibility to flavivirus infection. After ZIKV infection, mice deficient in the IFNAR1 gene develop severe disease, including hind limb weakness, paralysis, and death.^713–715^ Blocking with an anti-IFNAR1 mAb had similar effects. ZIKV-infected IFNAR1−/− mice developed disease in an age-dependent manner, and aged ZIKV-infected IFNAR1^−/−^ mice exhibited greater survival than young ZIKV-infected mice.^706,707,713^ Mice lacking both type I and type II IFNRs were more susceptible to ZIKV.^707,716^ ZIKV caused uniform lethality in AG129 mice, even at doses as low as 1 PFU.^713^ In addition, immunodeficient mice exhibit some of the typical symptoms of ZIKV infection in humans, including conjunctivitis, uveitis, and abnormal manifestations in the reproductive system, such as hematospermia and prostatitis.^717,718^ Immunodeficient mice can be used to study the pathogenesis of ocular and reproductive diseases associated with ZIKV infection.

Mice with acquired immunodeficiency are also susceptible to ZIKV infection.^719^ When mice were given the immunosuppressive steroid dexamethasone before and after ZIKV challenge, the infected mice exhibited weight loss, viremia, and disseminated infection, with viral RNA and antigens detected in many tissues. The discontinuation of dexamethasone after 9 days of infection resulted in rapid deterioration of the mice. This study demonstrated that exogenous type I interferons improve clinical outcomes, and a dexamethasone-induced immunosuppression model could be used to investigate the mechanisms of host immune response-related damage and countermeasures against ZIKV infection.

Much attention has been given to ZIKV infection in pregnant women and the corresponding fetal microcephaly and other associated diseases.^720,721^ IV or SC infection of ZIKV-infected pregnant IFNAR1^−/−^ C57BL/6 mice caused placental infection, fetal brain injury, and fetal death.^722^ When IFNAR1 female mice were mated with WT sires, the resulting fetuses were considered IFNAR1 heterozygotes. Severe consequences were observed despite the ability of the fetus to respond to type I interferon, suggesting that the type I interferon response in the fetus was not sufficient to protect the fetus from ZIKV-induced damage. Likewise, pregnant wtC57BL/6 mice were infected with ZIKV after receiving the anti-IFNAR1 mAb. The fetus exhibited intrauterine growth restriction with high levels of ZIKV infection in the placenta and fetal head.^722^ Thus, a gestational fetal ZIKV pathogenic model was successfully established in mice, which could be exploited to determine the cellular tropism and mechanisms of transplacental transmission of ZIKV to the fetus. IV inoculation of pregnant WT mice with the ZIKV strain caused fetal abnormalities,^723^ which enabled us to investigate the mechanisms of ZIKV teratogenicity in more immunocompetent animals. Direct injection of ZIKV into the cerebroventricular space of fetuses developing in WT ICR or C57BL/6 mice resulted in decreased brain size, thinning of cortical layers, reduced numbers of cortical neural progenitors, and death of immature and mature neurons within 3 to 5 dpi.

#### Syrian golden hamsters

ZIKV infection of STAT2 knockout hamsters resulted in morbidity and mortality rates of 37 and 42%, respectively. Viruses were observed in the uterus, placenta, brain, spinal cord, and testicles. ZIKV has been detected in several placentas and fetal brains of hamsters.^724^ However, immunocompetent hamsters inoculated intraperitoneally with the ArD 41525 strain did not exhibit any clinical signs other than viremia or ruffled fur.^725^

#### Guinea pigs

Guinea pigs are susceptible to ZIKV infection and harbor viral antigens in multiple tissues, including the brain and parotid glands.^726^ After inoculation with ZIKV, the guinea pigs developed clinical signs, including fever, lethargy, a hunched back, ruffled fur, and decreased mobility. ZIKV RNA was detected in the serum and whole blood at 2 dpi. Viremia and antigenaemia were observed in infected animals.^727^ No infectious virus was detected in any of the infected animals. ZIKV replication was observed in the spleen and brain, and the brain harbored the highest viral load. Increases in the levels of IL-5, IL-12 (p70), G-CSF, MCP-1, TNF-α, G-CSF, MCP-1, MIP-1α, LIX, fractalkine and VEGF were observed at 2 dpi.^728,729^ In pregnant guinea pigs, ZIKV was detected at low levels in reproductive and placental tissues and caused fetal infection.^730^

#### Ferrets

ZIKV infection has been investigated in pregnant ferrets. On the 21st day of pregnancy, the animals were infected with ZIKV-PRVABC59. Infected animals developed clinical signs such as fever, brain deformities, and hypertrophied vessels, and 28.57% (2/7) of the infected ferrets died. However, the outcomes were variable within and across litters and ranged from the absence of observable abnormalities to prominent changes.^731^

#### Nonhuman primates

##### Rhesus macaques

Rhesus macaques have shown susceptibility to ZIKV.^732–734^ Infected macaques developed rash, fever and conjunctivitis. ZIKV infection causes acute and chronic inflammatory and proliferative changes. The virus initially targets the intestinal tract, spleen, and parotid glands and persists in multiple tissues, such as neuronal, spleen, lymphoid, and joint/muscle tissues and reproductive tissues.^735–737^ ZIKV is vertically transmitted to macaque fetuses at a very low dose during pregnancy and causes maternal viremia, placental dysfunction, and fetal death in utero. ZIKV has been detected in a variety of brain regions, including the caudate nucleus, parietal lobe, cortex, and amygdala.^738^ ZIKV infection during pregnancy results in early pregnancy loss, fetal loss and fetal and neonatal developmental abnormalities; this disease is known as congenital Zika syndrome (CZS).^739–741^ Symptoms of CZS include cerebral and cranial abnormalities, neurodevelopmental delays, seizures, joint contractions, hearing loss and visual impairment.^742^ Histopathologic brain lesions included microcalcifications, hemorrhage, necrosis, vasculitis, gliosis, and apoptosis of neuroprogenitor cells.^743^ Central nervous system abnormalities associated with virus replication and local neuroinflammatory responses were also observed in ZIKV-infected fetal brains, and these abnormalities impacted brain development over time.^744–748^ In addition, ZIKV infection during early pregnancy impacts fetal retinal development and causes congenital ocular anomalies.^749^ ZIKV infection via a needle results in systemic symptoms and virus dissemination. Infection via mosquito bite causes mild or asymptomatic disease, which is similar to the disease observed in human patients; delayed ZIKV replication; and virus present only in hemolymphatic tissues, female reproductive tract tissues, the kidney, and the liver.^750^

##### Cynomolgus macaques

Cynomolgus macaques have been used to study the pathogenesis of and evaluate medical countermeasures for ZIKV infection.^751,752^ During mosquito bite infection, ZIKV RNA loads peak at 4~8 dpi and range from 3 × 10^3^–6.4 × 10^5^ copies/mL in cynomolgus macaques.^753^ After infection, sole fever and mild weight loss were observed.^736,754^ Animals infected with ZIKV-FSS13025 developed peak viremia at 3–4 dpi but showed a limited ability to spread the virus to mosquitoes, even during peak viremia.^755^ The clinical manifestations of ZIKV infection in cynomolgus macaques were strain- and challenge route-dependent. Macaques infected with ZIKV IBH30656 (of the African lineage) develop mild erythema or even asymptomatic lesions. Compared to animals challenged with Asian lineage strains, African lineage strain-inoculated animals did not produce productive virus in serum. Moderate viral loads of less than 300 copies/mL were detected in saliva and urine samples of animals challenged with Asian lineage strains. In addition, the highest viral loads were detected in testis samples, which supported the transmission of ZIKV by sexual contact.^756^ Peak viral RNA titers were significantly greater in macaques infected with ZIKV-SG than in those infected with ZIKV-Brazil or ZIKV-FP, whereas the ZIKV-Brazil and ZIKV-FP strains elicited more innate immune cells.^754^ SC challenge decreased the level of the growth factor VEGF in colorectal and cervicovaginal tissues.^757^ ZIKV infection via the intravaginal or intrarectal route was high in cynomolgus macaques, in which 3.5 log_10_ PFU/mL and 4.8 log_10_ PFU/mL of ZIKV, respectively, were detected; moreover, ZIKV infection significantly changed clinical laboratory parameters such as blood urea nitrogen (BUN), aspartate transaminase (AST), alanine transaminase (ALT), alkaline phosphatase (ALP), erythrocytes, monocytes, and eosinophils. When mosquito–human transmission is absent, ZIKV transmission through sexual intercourse can serve as a virus maintenance mechanism and may increase the likelihood of ZIKV establishment and transmission in areas that are free of the virus.^758^ Furthermore, repeated intravaginal inoculations of ZIKV induced potent protective neutralizing antibodies in cynomolgus macaques.^759^ Vaginal challenge causes proinflammatory responses in all mucosal tissues in the later stage of chronic infection.^757^ Compared with SC infection, ZIKV infection via the IV route induced greater serum viremia and cytokine and chemokine responses.^754^

##### Other NHPs

Other NHPs have also been applied for ZIKV infection.^760^ In male pigtail macaques, ZIKV replicates efficiently and shows broad tissue tropism.^761^ Intermittent rectal bleeding and rash developed in some individuals, while other typical symptoms were absent.^761^ In the absence of microcephaly, ZIKV induces substantial brain injury.^762^ Peripheral ventricular lesions developed within 10 dpi, with asymmetric changes in the occipital parietal lobe. Fetal autopsies revealed ZIKV in the brain, hypoplasia of white matter in the brain, hypergliosis of the periventricular white matter, and axonal and outdoor injuries.^762,763^

In marmoset monkeys, ZIKV causes rapid, high, acute viremia with neuroinvasion in peripheral and central nervous tissue but developed no observable lesions or clinical symptoms.^764–766^ After infection, the virus persists in lymphoid, neurological and reproductive tissues and body fluids such as semen and saliva for a long period of time.^764–766^ The virus can invade the placenta and fetal neural tissue, and viremia is detected in pregnant marmosets, causing spontaneous fetal loss and neurodevelopmental abnormalities.^767^ There are also reports that fetal brain damage is associated with a high maternal antibody titer, which underlines the potential pathogenesis of fetal brain damage.^768^

In baboons, ZIKV infection can cause viremia, rash and conjunctivitis. Viral shedding was detected in the mucosa and cerebrospinal fluid. High levels of ZIKV-specific IgM and IgG antibodies and increased IL2, IL6, IL7, IL15, and IL16 were observed in infected animals.^769^ ZIKV has been detected in the fetal cerebral cortex and other tissues and can be transferred from maternal to fetal through the placenta,.^770^ Placental pathology includes disruption of syncytiotrophoblast layers, delayed villous maturation, partially or fully thrombosed vessels, calcium mineralization and fibrin deposits.^771^ ZIKV infection caused fetal CNS lesions in pregnant baboons. The reproductive organs of male baboons can also be infected, characterized by the presence of ZIKV in sperm progenitor cells, penetration of spermatogenic tubular macrophages, and an increase in tumor necrosis factor alpha (TNF-α), especially in the vascular wall.^772^ Viruses can be transmitted by semen and are detected in the lymph nodes of pregnant animals.

### Medical countermeasures for treating Zika virus disease

Several ZIKV vaccines, including inactivated vaccines, DNA vaccines, mRNA vaccines, and protein subunit vaccines, have entered clinical trials. In addition, live attenuated virus vaccines and AdV-, MVA- and MeV-based viral vector vaccines were also under preclinical investigation (Table 14). Representatively, the Walter Reed Army Institute of Research developed a purified formalin-inactivated Zika virus vaccine (ZPIV), which was safe in mice and NHPs.^773^ In Phase I clinical trial, ZPIV was found to be safe and immunogenic when coupled with an aluminum hydroxide adjuvant.^774^

**Table 14:** Representative medical countermeasures for treating Zika virus infection

Classification	Name	Platform/Strategy	Stage	Efficacy/Benefic	References
Vaccines	Inactivated vaccines	Formalin-inactivated Zika virus	Clinical	Safe and immunogenic in humans	^774^
	GLS5700	DNA vaccine encoding prM-E sequence of African and Asian/American strains	Clinical	Immunogenic in mice and NHPs	^775^
	VRC5288/VRC5283	DNA vaccine encoding chimeric prM-E or prM-E	Clinical	Safe, tolerate and immunogenic	^776,777^
	mRNA vaccine	Non-self-amplified mRNA and nonintegrated vectors	Preclinical	Protect mice and NHPs	^778,779^
		Self-amplified mRNA	Preclinical	Immunogenic in mice	^780^
	MeV-prM-ENV	Measles virus vector expressing prM-ENV	Clinical	Immunogenic in mice and NHPs	^781,782^
	RHAd52-prM-ENV	RHAd52 vector expressing prM-ENV	Preclinical	Immunogenic in NHPs	^774^
Antibodies	ZIKV-117	Human monoclonal antibody targeting a unique quaternary epitope on the E protein	Preclinical	Broadly neutralized ZIKV African and Asian-American lineages and effective in mice	^784^
	ZKA78	Human monoclonal antibody targeting EDIII	Preclinical	Protect mice	^785^
Small molecular drugs	BCX 4430	RdRp inhibitor	Preclinical	Effective in mice	^789^

The following acronyms are used: membrane and envelope proteins (prM-E); rhesus monkey adenovirus serotype 52 (RHAd52); envelop protein domain III(EDIII); RNA dependent RNA polymerase (RdRp); nonhuman primates (NHPs)

Three DNA vaccines are in clinical trials. GLS5700 was developed by Inovio Company while VRC5288/VRC5283 was developed by NIAID. GLS5700 is designed to express prM-E sequences from African and Asian/American strains. GLS5700 was immunogenic in mice and NHPs.^775^ A Phase II clinical trial of GLS5700 is ongoing. Based on French Polynesia and early Brazilian ZIKV isolates, VRC5288 is designed by exchanging 98 amino acids from transmembrane and stem regions in the Japanese encephalitis virus (JEV) envelope protein with corresponding regions in the ZIKV E protein and expressing codon-modified ZIKV/JEV chimeric prM-E. In addition, VRC5283 contains an optimized complete ZIKV prM-E gene structure. Both VRC5288 and VRC5283 vaccines were immunogenic in mice and NHPs. VRC5283 is more effective than VRC5288 in preventing viremia and can produce a strong neutralizing antibody and T-cell immune response.^776^ In Phase II clinical trials, both vaccines were safe, well tolerated, and immunogenic.^777^

At least two strategies have been involved in the development of ZIKV mRNA vaccines. In one case, modified nucleotides and codon optimization were performed using non-self-amplified mRNA and nonintegrated vectors. This method increases the scale and duration of vaccine production by improving the stability of the ZIKV RNA vaccine and reducing the detection of intracellular innate immune sensors.^778^ In this manner, the ZIKV mRNA vaccine combined with lipid nanoparticles efficiently protected mice and NHPs after a single dose of vaccination.^779^ In other cases, self-amplified mRNA was applied, which enables the rapid production of a large number of transcripts and vaccine antigens and significantly activates innate sensors.^780^

Measles virus-vectored ZIKV vaccine expressing prM-ENV has entered Phase I clinical trial. It has been proven to be immunogenic in mice and NHPs.^781,782^ Using Rhesus monkey adenovirus serotype 52 (RHAd52) as a vector, a recombinant vaccine was developed by expressing prM-ENV of ZIKV, namely RHAd52-prM-ENV. It was immunogenic in rhesus monkeys and provided effective protection after a single dose of vaccination.^774^ One study compared four replication defective gorilla adenovirus (Chadox1) vector based ZIKV vaccine candidates. The results showed that the defective adenovirus vaccine expressing both prM and E without the transmembrane region, which obtained 100% protection and a lasting anti-envelope immune response in mice immunized without adjuvants.^783^

A panel of human mAbs was isolated from recovered ZIKV patients. A subset of antibodies recognizes diverse epitopes on the envelope (E) protein and exhibits potent neutralizing activity. One of the most effective antibodies, ZIKV-117, which broadly neutralizes African and Asian-American ZIKV lineages, was selected. ZIKV-117 recognized a unique quaternary epitope on the E protein dimer-dimer interface. In pregnant and non-pregnant mice, ZIKV-117 treatment markedly reduced tissue pathology, placental and fetal infection, and mortality.^784^ In another study, antibodies against E protein domain I/II (EDI/II) were shown to cross react between ZIKV and DENV but were less effective. The most potent neutralizing antibodies were ZIKV-specific and targeted EDIII or quaternary epitopes on infectious viruses. An EDIII-specific antibody, ZKA78, protected mice from lethal ZIKV infection.^785^ Interestingly, a subset of antibodies isolated from dengue virus infected patients also potently neutralizes Zika virus. These mAbs are complex with the conserved epitope in the envelope protein of Zika virus.^786^

Drugs for ZIKV have been developed based on the following directions: repurposing clinically approved drugs, viral replication-based phenotypic screening for inhibitors, and targeted drug discovery of viral proteins.^787,788^ Emricasan targets caspase 3 (caspase-3) to prevent cell death. The Z2 peptide targets ZIKV for entry into cells. 25-Hydroxycholesterol and chloroquine interfere with lipid homeostasis and autophagy, thus destroying the release of viral particles after endocytosis. Temoporfin, nitazoxanide, niclosamide and viperin block the activity of the NS2B/NS3 protease to prevent viral replication. Sofosbuvir, Merimepodib, N-(4-hydroxyphenyl) retinamide, 7-deaza2’-C-methyladenosine, NITD008, BCX4430 and ribavarin block the activity of ZIKV NS5 polymerase to prevent virus replication. Ribavarin and merimepodib block hypoxanthine nucleotide dehydrogenase. Niclosamide, EGCG, and cavinafungin block CDKs to prevent virus replication. For example, BCX 4430 is a selective inhibitor of viral RdRp. It acted on NS5 polymerase and promoted the synthesis of viral RNA. In AG129 mice BCX 4430 significantly reduced viremia and protected 87.5% of the mice.^789^ The compound is currently in the Phase I clinical trial.

## Gaps, directions, and prospects

While remarkable progress has been made in the prevention and control of WHO high priority pathogens, much more issues need to be noted and addressed. The most direct bottleneck in studying WHO high priority diseases is the availability of animal models, as currently available animal models suffer from several limitations and challenges. In addition, the selection of animal models is a critical step, and may even determine the direction of the outcomes. To obtain effective prophylactic and therapeutic approaches, a prerequisite is the clarification of immune correlates of protection. Preferably, to accomplish a better connection between correlates of protection across animal experiments and clinical studies. Similarly, accurate and targeted therapies require a better understanding of disease mechanisms. Given the abundance of subtypes and variants, as well as the different levels of disease severity, comorbidities, and sequelae involved in WHO high priority diseases, corresponding countermeasures should be taken. Novel technologies may aid and accelerate breakthroughs in the prevention and control of infectious diseases. Here, we discuss these aspects of gaps, directions, and prospects in detail.

### Limitations and challenges of currently available animal models

One of the paramount challenges in acquiring animal models is the differences in physiology, genome and immune system between animals and humans. These differences contribute to the varied pattern of host interactions, which hinders research into pathogenesis and immune-based prevention and treatment strategies.^790^ Moreover, susceptibility to specific pathogens is determined by functional receptors. For WHO high-priority pathogens, NHPs, in some cases ferrets, are naturally susceptible animals.^15–18^ However, most if not all, accessible, economically abundant experimental animals, such as guinea pigs, hamsters, and mice, are insusceptible to these pathogens. Therefore, additional techniques, including immunodeficiency, virus adaptation, receptor knock-in, and humanization, should be adapted to establish effective infections and diseases.^15–18^ Although the aforementioned approaches have enabled lethal infections and recapitulation of certain aspects of WHO high-priority disease in rodents, there are significant differences. For instance, most rodent models are solely susceptible to EBOV via the IP route, which differs from what has been observed in humans, indicating a distinct pattern of infection, immune response, and disease progression.^15^ Hence these rodent models may lead to misleading medical countermeasures. For example, due to differences in the function and distribution of the ACE2 receptor, the efficacy of viral vector-based mucosal COVID-19 vaccines tested in mice was opposite to that of NHPs or humans.^460,791–794^ These results underscore the significance of selecting appropriate animal models under specific conditions.

Another challenge in extrapolating human clinical conditions from animal models is complex genetic and environmental factors in humans.^795^ Generally, experimental animals have a clear genetic background, are raised in a barrier environment, and are free from pathogenic microorganisms. However, there are significant racial, genetic, and environmental differences between populations. In addition, factors such as the economy, region, and climate further exacerbate these differences. Humans are constantly exposed to microbial environments, and the immune system is tightly regulated.^796^ These issues complicate the interpretation of preclinical data in animal models. In preclinical studies, abundant vaccines and antibodies have been proven to be effective and offer potential benefits. However, only a limited number of products have entered human clinical trials, and few have been approved.^797^ This could be largely attributed to the poor connection between preclinical data and clinical outcomes. To address this issue, immune correlates of protection against specific pathogens should be established between animal models and humans. Ideally, the establishment of immune correlates of protection in animal models would allow for immune bridging, which would improve the prediction and interpretation of the target product in humans.^798^ However, animal models that provide early warning signs of VAED and ADE effects are also urgently needed. This would be significant for timely responses to potential side effects or hazards caused by medical countermeasures.

To accomplish effective infection, novel sensitization strategies are involved, which may introduce additional concerns. Specifically, immunodeficient animals are expensive and need to be maintained in a barrier environment. These animal models are inapplicable for vaccine evaluation and corresponding immune correlates of protection. According to the virus adaptation model, the adaptation process may introduce additional mutant sites than the original virus, which hinders the translation and predictability of the virus for the treatment of the corresponding human disease.^353–358,799^ In addition, despite the transgene model enabling the knock-in of functional receptors, the precision and distribution of receptors should be carefully explained.^333–337^ In contrast, several naturally susceptible small animal models of WHO high-priority pathogens, such as ferrets of filoviruses, beta-coronavirus infections, and minks/hamsters of beta-coronaviruses, were found. These small animal models present promising alternatives to NHPs, given the adequate characterization of simulated human diseases. NHPs are the only animal species available that can recapitulate the stated aspects and are thus considered the gold standard. Unfortunately, NHPs are expensive, inaccessible and associated with ethical issues. Most of the major works in animal ethics strongly oppose the current use of NHP animals for research, ranging from advocating a restrictive stance to advocating abolition.^800^ Four key issues that come to the core in animal ethics are autonomy or self-determination, harm and benefit, justice, and vulnerability. Moreover, delicate differences between NHP species should be sufficiently documented in terms of specific pathogens.

Moreover, there are abundant subtypes or variants of WHO high-priority pathogens. These subtypes or variants exhibit distinct pathogenicity, transmission capacity, and clinical disease severities in humans.^1,801^ However, no animal models fully recapitulate the subtype or variant-associated disease severity in humans. In this regard, resource imbalances exist, and models for some viruses are more developed than those of others.^15^ For example, animal models for EBOV infection have been fully investigated, but limited models are available for TAFV infection. In the near future, the development and characterization of animal models for less known filovirus subtypes is needed.

Overall, accurate animal models and their indications are used to clarify the correlates of protection, subtypes or variants, possible adverse effects, disease severity, duration and recovery period, risk of reinfection, and complications. The above aspects have important implications for patient management, serotherapy, vaccine design, and drug administration during recovery.^802^ Here, we dissect these aspects of the application and deployment of animal models.

### Selection of animal models

According to the “two-animal rule” guidlines of the FDA, to better support the approval or licensure of medical countermeasures, the efficacy of a candidate product should be tested in “more than one animal species expected to react with a response predictive for humans”.^803^ Therefore, any candidate product should have potential benefits in at least two animal models, preferably a small animal model in addition to an NHP model, to determine the overall outlook for the safety and efficacy of the target product. More specifically, there is some evidence and data needed to demonstrate the effectiveness of a product when human efficacy studies are not ethical or feasible. I. A reasonable understanding of the pathological and physiological mechanisms of biological substances and prevention or significant reduction effectiveness of products. II. Animal study endpoints are obviously related to the expected benefits to humans, usually to improve survival or reduce the major incidence rate. Product kinetics and pharmacodynamics data or information should allow the selection of effective doses for humans. III. Animal efficacy studies should substitute for human efficacy trials, following the practice of adequate and well-controlled human efficacy studies, and their endpoints prove to have important clinical benefits. The effect is demonstrated in more than one animal species expected to react with a response predictive for humans, unless the effect is demonstrated in a single animal species that represents a sufficiently well-characterized animal model (meaning the model has been adequately evaluated for its responsiveness) for predicting the response in humans. Owing to the balance between susceptibility, cost, accessibility, and feasibility, mice, hamsters, and guinea pigs often serve as preliminary animal models for initial experiments. These small rodent models are available and can be easily manipulated. Subsequently, NHPs could be used as secondary animal models, which would provide further insights into the candidate product.

**Fig. 2:**
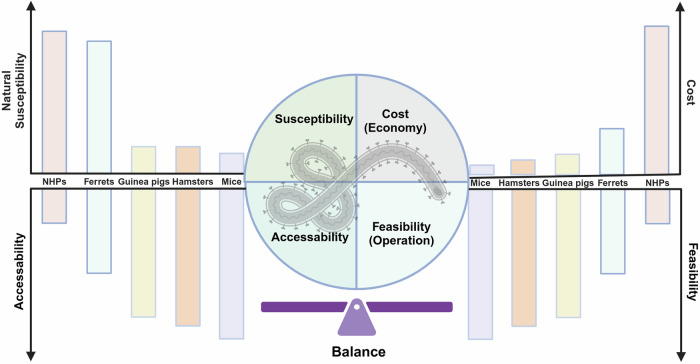
Choice of animal model. Animal models for filoviruses are taken as examples, and the susceptibility, cost, accessibility, and feasibility of nonhuman primates (NHPs), ferrets, guinea pigs, hamsters, and mice are presented. (Created in BioRender)

Consequently, the choice of preliminary or secondary model largely depends on the purpose of the study, carefully weighing accessibility, economy, and the provided conditions (Fig. 2). Overall, naturally susceptible animal models have contributed to the exploration of pathogenesis, transmission, and clinical manifestations, while receptor-transduced models help clarify receptor-ligand interactions and interspecies transmission. Other models established by novel approaches also reproduce aspects of pathogen-specific issues, which warrant further definitions.

### Clarification of correlates of immune protection

**Fig. 3:**
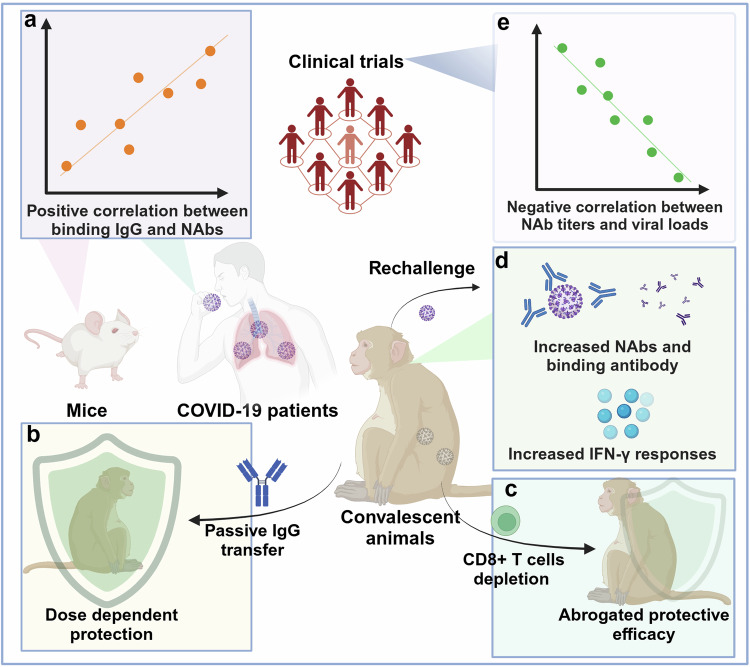
The determination of immune correlates in animal models and immune correlates in COVID-19 patients are taken as examples. **a** In animal models and coronavirus disease 2019 (COVID-19) patients, a positive correlation between severe acute respiratory syndrome coronavirus 2 (SARS-CoV-2) was confirmed. **b**, **c** In COVID-19 convalescent animals, passive transfer of serum IgG protects naïve animals in a dose-dependent manner, while CD8^+^ T-cell depletion abolishes this protective effect to some extent. **d** SARS-CoV-2 rechallenge in convalescent animals increased neutralizing antibody (NAb), virus-specific binding antibody and IFN-γ responses. **e** In large-scale human clinical trials, a negative correlation between NAb titers and viral loads was noted. Taken together, these findings suggest that NAbs and virus-specific IgG are responsible for improved protection against COVID-19, while cellular immunity partially contributes to this protection. (Created in BioRender)

The concept of immuno-bridging requires standardized immunological assays to quantify correlates of protection across animal experiments and clinical studies. Immunobridging would simplify and expedite the expansion of approved vaccines to larger groups of people without costly or lengthy efficacy studies. It will also assist in determining other aspects of the immunization program, drug administration and prognosis. In fact, correlates of protection are always more complicated than expected, rendering a universal correlate of protection difficult to achieve.^804^ In this process, animal models are valuable for validating correlations of protection through depletion or passive transfer studies of antibodies or T cells, which offer an in-depth examination of immune parameters (Fig. 3).

Representatively, animal models of beta-CoV infection have fostered the understanding of immune correlates. It has been described in an animal model that neutralizing antibodies, which are positively correlated with virus-specific IgG, are responsible for a better prognosis and protection in patients with COVID-19.^433,805–808^ Moreover, CD8^+^ T cells may also contribute to protection if antibody responses are suboptimal.^804^ However, a dysregulated cellular immune response, particularly cytokine storms, contributes to lethal outcomes.^809^ Similarly, in EBOV infection, massive lymphocyte apoptosis has been proven to be a marker of poor prognosis in mice, NHPs, and humans and is characterized by actively activated proinflammatory cytokines.^66,67,139,810,811^ In both mice and NHPs, lymphocyte activation, increased CD44^+^ T cells, and increased lymphocyte numbers were biomarkers observed at later stages of infection. That is, mice and NHPs infected with EBOV have obviously similar immunoreactive characteristics to those of humans. These results help to elucidate disease progression, biomarkers, and immune parameters, and help validate correlates of protection or dysregulated immunity that correspond to severe disease in humans. Furthermore, when data from large-scale clinical trials are well excavated, connected and modeled with results from preclinical studies, animal models could better predict the clinical efficacy of candidate medical interventions.

### Better understanding of disease mechanisms

One of the basic considerations in developing animal models is their potential to recapitulate disease pathogenesis. In this regard, the biases of each model should be fully considered. To a large extent, diseases caused by WHO high-priority pathogens are attributed to virus-host interactions, overwhelming viral multiplication, followed by dysregulation of immune parameters and subsequent acute injury and multiorgan failure.^13,812^ In cases that immunodeficient animals facilitate and enable sensitive infection and symptom onset, these models may provide a better understanding of disease mechanisms in terms of antiviral signaling pathways. On the other hand, naturally susceptible animal models can provide an overall profile of disease mechanisms in humans. In contrast, transgenic and humanized models offer insights into functional receptor-associated virus-host interactions, as well as comprehensive immune systems and patterns comparable to those of humans. For instance, to determine the physiological expression of a series of immune components with regard to quantity, location and time in humans, MISTRG629 mice were engineered based on homologous gene replacement approaches between humans and mice.^813–819^ When engrafted with human hematopoietic stem/progenitor cells (HSPCs), these mice have a comprehensive immune system comparable to that of humans.^817,820^ For humanized mice, implantation of human tissue could further expand the tropism of human pathogens. When lung-implanted mouse was incorporated into bone marrow/liver/thymus humanized mice, this model supported infection with a broad range of human pathogens and enabled robust adaptive immunity following pathogen infection.^821^

Vaccine-associated enhanced disease (VAED) and antibody-dependent enhancement (ADE) are two major issues of concern in the development of medical responses to infectious diseases. These adverse events severely affect individuals infected with a pathogen after receiving a prior vaccine or infection-induced antibodies, resulting in enhanced infection via FcγR-expressing cells.^822,823^ There has been in vitro evidence of ADE for EBOV, MARV and beta-coronavirus.^824–826^ ADE was also observed for EBOV mAbs at subneutralizing concentrations, and this effect was not epitope restricted or independent of the neutralizing capacity or subclass of the mAbs.^827^ Fcγ receptor blockade reduced but did not abolish the ADE. A mAb that interacts with Fc receptors at a low dose partially protects mice. These results suggest that ADE disturbs antibody-mediated protection and facilitates the dissemination of filovirus infections. Some studies with NHPs suggest the possibility of ADE. Treatment of EBOV-infected macaques with macaque-origin convalescent serum failed to protect the animals and resulted in greater viral titers at the time of death/moribund conditions.^828,829^ Although protection with extremely high doses of antibodies was achieved, the failure of EBOV treatments with moderate antibody doses and increased viral loads suggest the possibility of ADE.^830,831^

Several studies have noted that RBD-specific SARS-CoV-2 monoclonal antibodies can cause ADE in vitro, which enhances Fcγ-mediated entry of SARS-CoV-2 into cell lines. The ADE effects could be eliminated by introducing an LALA mutation at the FC end.^832,833^ In addition to RBD-specific antibodies, N-terminal domain (NTD)-specific antibodies also have ADE effects in vitro via a mechanism different from that of RBD-specific antibodies. NTD-specific antibodies do not depend on the FCγR. Instead, the “open” conformation of the RBD is increased by affecting the NTD-RBD interaction, which facilitates the binding of the S protein to ACE2.^834^ The above results collectively showed that the adverse effects of the SARS-CoV-2 antibody involved at least two mechanisms: FC-FCγRII and S-ACE2 interaction-dependent. Interestingly, neither RBD nor NTD-specific antibodies, such as those against increased viral load or inflammation, had obvious adverse effects on mice or NHPs. In contrast, these antibodies had certain protective effects, indicating that in vitro ADE results cannot represent comprehensive clinical results in vivo.^835^ Although SARS-CoV-2 may enter macrophages through FC-mediated ADE antibodies in vivo, it may not replicate effectively in macrophages. In addition, FC mediated effector functions may also play a protective role, but the specific mechanism involved is still unclear.

Preclinical data indicating increased eosinophilic infiltration and pulmonary response in mice after treatment with SARS-CoV and MERS-CoV inactivated vaccines.^836,837^ In a ferret model, immunization with a modified vaccinia virus Ankara-based SARS-CoV vaccine led to enhanced hepatitis in ferrets.^390^ To avoid these effects, strong T-helper 1 (Th1) CD4-mediated responses are preferable.^838^ Moreover, alum-adjuvanted and formaldehyde-inactivated whole-virus vaccines were utilized to address evidence of VAED. In rhesus macaques, there was no evidence of enhanced lung pathology, and a rapid increase in NAbs was observed after challenge with SARS-CoV-2 following formalin-inactivated vaccine inoculation.^839^ In hamsters and ferrets, mild transient enhanced disease was observed at 7 dpi, which was resolved by day 15. Notably, hamsters showed suboptimal NAb levels and protection against challenge because formaldehyde treatment of SARS-CoV-2 reduces exposure of the RBD, and their lung cytokines were markedly skewed toward Th2 cells.^839^ Moreover, in K18-hACE2 mice immunized with a very impure formalin-inactivated SARS-CoV-2 preparation and an aluminum hydroxide-based adjuvant, the onset of SARS-CoV-2 replication and disease occurred earlier than that in naïve control groups or mRNA-vaccinated animals.^840^

**Fig. 4:**
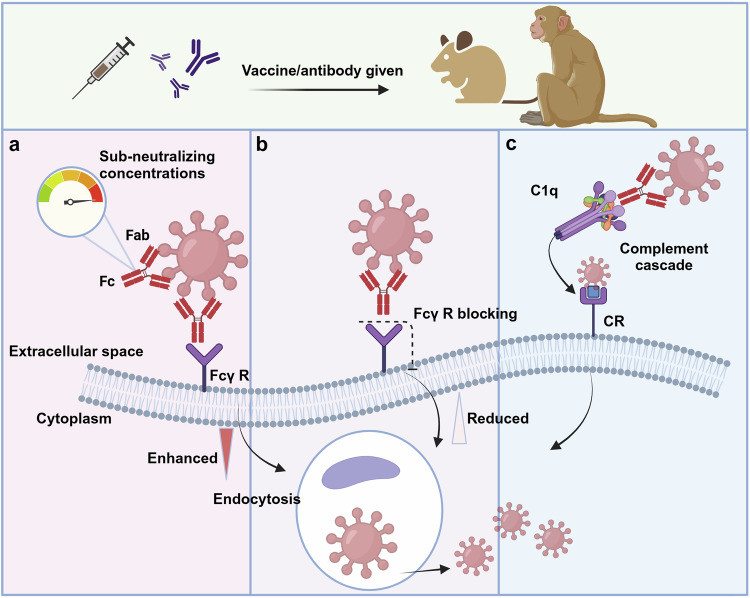
Validation of potential vaccine-associated adverse effects (VADEs) and antibody-dependent enhancement (ADE) in animal models. **a** Antibodies at subconcentrations facilitate virus entry through Fc-FcR recognition. **b** Fcγ receptor blockade reduced virus entry and endocytosis. **c** Deposition of the complement cascade (C1q) facilitates virus entry and endocytosis. (Created in BioRender)

Taken together, these results on ADE and VADE highlight that a tight combination should be achieved between in vitro results and in vivo performance in animal models (Fig. 4). Together with a thorough dissection of the underlying mechanism, these adverse effects could be better handled during the R&D process for WHO high-priority pathogens.

### Concerns of WHO high-priority pathogen subtypes and variants

WHO high-priority pathogens include a large group of viruses of the genus Filovirus, coronavirus, bunyavirus, etc., which can be further divided into several subtypes. For example, the Ebola virus family consists of six members.^1,841,842^ They exhibit varied clinical signs of disease and severity in humans and NHPs. Moreover, beta-CoVs are highly susceptible to the accumulation of mutants. For SARS-CoV-2, more than five variants of concern (VOCs) have been noted.^842^ Additionally, these variants exhibited altered transmission transmissibility and pathogenicity. To distinguish between these subtypes and variants, specialized animal models are needed.

As a natural model of Ebola virus infection, members of the Ebola virus family were evaluated in ferrets. EBOV- and SUDV-infected ferrets exhibit symptoms and lesions typical of humans.^96–98,100^ Compared with EBOV- and SUDV-infected animals, ferrets infected with BDBV exhibit a prolonged disease course, which is consistent with what has been observed in humans.^96,98^ However, despite asymptomatic infection in humans, RESTV is highly lethal to ferrets, humanized mice, and NHPs.^77,101,102^ Like those of EBOV and SUDV, the symptoms of RESTV infection in ferrets showed similar signs of disease and outcomes.^103^ Additionally, RESTV-infected macaques were similar to those infected with EBOV or SUDV and died at 8–10 dpi.^146–148^ Notably, RESTV even caused an outbreak in cynomolgus macaques in 1996 in the Philippines.^145^ In contrast to lethal infections in humans and NHPs,^149^ TAFV infection can be mild or nonlethal in ferrets.^104^ Additionally, infections with MARV or the ravn virus (RAVV) did not cause obvious signs in either adult or naïve ferrets.^105,106^ The above results suggested that disease severity is altered in ferrets and even in NHPs for some filoviruses. Fortunately, humanized mice may help address this issue, as case fatality rates of different Ebolavirus subtypes in humans have been recapitulated. As described, huNSG-A2 mice were significantly less susceptible to Makona virus than to Mayinga virus. These results suggest that humanized mice are a putative model for distinguishing the pathogenicity of various filovirus subtypes.^77^

For SARS-CoV-2, VOCs show enhanced transmission ability in comparison with the original SARS-CoV-2 strain in hamsters, ferrets, and white-tailed deer, which is similar to what has been observed in humans.^801,843,844^ This could be attributed, at least partially, to the presence of key substitutions in the spike protein, such as N501Y, which improve the receptor‒ligand affinity. This aromatic N501Y substitution is associated with increased transmission in humans but also allows infection in wild-type mice via the mouse ACE2 receptor, which was confirmed in SARS-CoV-2-adapted mouse models.^845^ These results open the possibility of using wild-type mice as a potential animal model of SARS-CoV-2 infection, as these mice are insusceptible to the original SARS-CoV-2 strains. Moreover, the possibility of interspecies transmission should be closely monitored since emerging variants may expand their tropism toward other animal species and eventually become resistant to ancestral viral strains, becoming novel secondary viral reservoirs.^846^ Fortunately, none of these variants seemed to show enhanced virulence in hamsters or NHPs, although increased proinflammatory cytokines were observed in hamsters infected with the alpha strain.^847^ In rhesus macaques and African green monkeys, beta-strain infection resulted in milder symptoms than infection with the ancestral B.1 and alpha strains.^413^ Conversely, studies in nonnaturally susceptible animals, such as K18-huACE2, revealed that infection with the beta VOC resulted in enhanced infectivity and quicker disease progression than infection with one of the ancestral variants (B.1). This increase in infectivity could be largely due to increased expression of interferon antagonist proteins induced by some VOCs.^848^ One study showed reduced fitness in response to beta VOCs in mice in competition trials. The increase in mutations leading to increased processing and fusion by the S protein of SARS-CoV-2 may be attributed to these differences.^849^ Overall, animal models of VOCs mimicked the characteristics of VOCs in humans and helped reveal associated mutation sites and potential mechanisms for enhanced transmissibility. Similarly, animal models will be essential for evaluating variant-associated pathogenicity and transmissibility and for dissecting and warning of potential risk. Currently available SARS-CoV-2 animal models show a limited duration of virus replication; therefore, we explored whether, in particularly, drug-resistant variants may emerge with suboptimal doses of either mAb or small molecule antiviral. It will therefore be important to develop SARS-CoV-2 infection models in which the virus replicates sufficiently high titers for extended periods of time without causing severe pathology, allowing for the assessment of the emergence of resistant variants against vaccines or therapies.

A concern with COVID-19 is reinfection with VOCs and virus shedding, which increases the possibility of asymptomatic infection and transmission. In this regard, hamsters reinfected with VOCs were indeed shown to shed SARS-CoV-2 for a number of days.^850–852^ However, infected cats do not shed enough virus for transmission to cohoused naïve animals.^853^ These results are in agreement with the clinical findings that although breakthrough infection could occur in vaccinated individuals, the transmission of delta VOCs from these individuals may be substantially reduced in comparison with that in nonvaccinated individuals. Importantly, virus shedding in hamsters, ferrets, and NHPs was reduced by IN vaccination,^854,855^ suggesting the potential value of mucosal vaccines for controlling VOC expansion.^101,102^

### Focusing different levels of disease severity, comorbidities, and sequelae

In the clinic, WHO high-priority pathogens present different degrees of disease severity, which are determined by the virus strain involved, host genetics and environmental factors. For example, the clinical course of COVID-19 can range from asymptomatic to severe.^842^ Pending the identification of animal models, one of the main prerequisites remains the clarification of animal models that recapitulate a given degree of disease severity. Hamsters have been defined as mild to moderate models of COVID-19 in humans; these individuals develop respiratory disease and display some important clinical hallmarks, such as anosmia, neurotropism, and vascular inflammation, after SARS-CoV-2 infection.^856–858^ However, there are disconnections between pathological examination and clinical signs. Severe interstitial pneumonia but mild to moderate clinical signs were observed in SARS-CoV-2-infected hamsters. Moreover, male hamsters exhibit more severe lung lesions than females and less efficient antibody responses.^378,859^ Indeed, male COVID-19 patients exhibit greater levels of proinflammatory cytokines and reduced T-cell-mediated immunity than female patients.^860^ Mouse-adapted SARS-CoV-2 and ACE2 transgenic mice also develop pathological signs of pneumonia that range from mild to severe. In some instances, mice also develop anosmia, a common manifestation of human COVID-19. SARS-CoV-2 is highly transmissible among minks via direct contact and respiratory droplets.^401–403^ Importantly, SARS-CoV-2 variants have emerged in minks, and relevant transmission from minks to humans has been confirmed.^404^ Among all COVID-19 animal models, minks are the most susceptible due to their functional receptor ACE2.^405,406^ The original SARS-CoV-2 strain replicates extensively in both the upper and lower respiratory tracts, leading to severe pathological injury and causing up to 20% weight loss.^403^ Additionally, mink intratracheally challenged with 10^6^ TCID_50 of_ SARS-CoV-2 Omic mimicked the pathological features of severe COVID-19. Surprisingly, the amount of viral RNA reached 7.15 log_10_ RNA copies/mL in nasal lavage fluid and 6.73 log_10_ RNA copies/mL in throat swabs at 1–2 dpi.^408^

Defining animal models for different levels of disease severity caused by other pathogens is also needed. These models could accurately support preclinical research into prophylaxis and therapy for different groups of people, guide administration in clinical practice, and uncover disease mechanisms and biomarkers.

WHO high-priority pathogens are always coupled with typical signs of disease, comorbidities, and sequelae. For instance, LASV causes SNHL, while ZIKV leads to congenital microcephaly. In response, animal models recapitulating these comorbidities/sequelae would provide benefits in terms of uncovering potential underlying mechanisms and evaluating medical interventions. STAT1^−/−^129Sv mice infected with LASV presented typical SNHL, similar to the clinical outcome of patients infected with LASV/LF2384 and LASV/LF2350.^241,245^ The STAT1^−/−^ model is the only small animal model of SNHL available. Histological examination revealed severe damage to the inner ear with a marked reduction in the number of outer hair cells. The inner hair cells remained intact. Hearing-bearing mice also exhibit impairment of the auditory nerve, while the nearby facial nerve remains intact. There was marked vacuolization of the spiral ganglion, thinning of the stria vascularis, expansion of Reissner’s membrane, and blood cell infiltration into the iliac crest. Viral antigens are present in vascular-rich areas where there is also significant infiltration of CD3^+^ lymphocytes, suggesting an immunopathological mechanism of hearing loss.^245^

For COVID-19, aged people are more vulnerable to SARS-CoV-2 infection, which correlates with immune senescence characterized by suppressed type I IFN responses, antigen presentation, and reduced T-cell responses, which leads to delayed viral clearance.^500,861^ There are abundant animal models that reflect age-dependent COVID-19 in humans. The severity of mouse-adapted SARS-CoV-2 infection in C57BL/6 and BALB/c mice is age dependent.^357^ Young mice are resistant to SARS-CoV infection, which is regulated by a STAT1-dependent but interferon receptor-independent mechanism, while aged mice exhibit greater weight loss, clinical signs, and pathology under the same conditions.^357,862^ The positive age-dependent severity of SARS-CoV infection was attributed to, at least to a large extent, increased inflammation in the lung, which has also been observed in humans.^863,864^ In ferrets, aged animals exhibit prolonged fever and more obvious weight loss than do their young counterparts.^400^ More severe lung pathology, higher viral titers, and a higher risk of transmission were observed. These phenomena were confirmed with findings in older patients. Age-related COVID-19 was also observed in rhesus macaques, baboons and cynomolgus macaques and presented as persistent viral pneumonia, impacting antibody responses.^431^ Consequently, there is increased viral shedding, more severe lung pathology, greater levels of proinflammatory cytokines, and greater body weight loss.^430^ Due to the high degree of biological similarity between NHPs and humans, NHPs could be powerful tools for revealing the mechanism by which age results in delayed or impaired antiviral immune responses and deficiency in immune homeostasis.^865^ Cardiovascular disease (CVD) and diabetes mellitus (DM) are the two most common chronic comorbidities that increase the severity and mortality of COVID-19. To better understand the potential underlying mechanism and assist corresponding countermeasures, animal models that recapitulate comorbidities of COVID-19 were established. After hACE2 transduction and SARS-CoV-2 infection, preexisting CVD development results in enhanced inflammation, viral invasion, and apoptotic pathway activation. Viral infection increased fasting blood glucose levels and reduced the insulin response in a DM model.^866^ There are also approaches to induce comorbidities, such as diabetes and obesity, through changes in diet. Diet-induced obesity in mice resulted in more severe disease upon SARS-CoV-2 infection.^845^ Finally, to respond to groups of people with comorbidities who are at high risk of severe disease, animal models with comorbidities are needed to dissect the role of infection versus comorbidity in disease severity. These efforts focus on multiple directions based on clinical needs and ultimately expedite timely and adequate responses to public health emergencies.

### Emerging technologies for prevention and control of infectious disease

Classic animal models are always concerned with ethical issues and lack the ability to replicate human genetic variation or study human-specific pathogens. On the other hand, in vitro standards, cell lines, do not recapitulate the complex microenvironment or disease process that occurs throughout the organism or at multiple organ levels.^867^ Therefore, novel models have been developed to bridge the gap between transformed cell lines and animal models based on the principle of “reduction, replacement and refinement”. Derived from pluripotent and tissue stem cells, organoid models provide ex vivo insights into pathogenesis, the host response, and the features necessary to develop preventive and therapeutic treatments.^867^ Currently, organoid models recapitulate many characteristics of respiratory, gastrointestinal, and neuronal host–microbe interactions.^868–870^ Studies of receptor identification and distribution, tropism, and local epithelial response in SARS-CoV-2-infected lung organoids have been performed. The lung organoid model is permissive to SARS-CoV-2 infection and shows robust induction of chemokines upon SARS-CoV-2 infection.^868^ Colonic organoids that express ACE2 and are susceptible to SARS-CoV-2 infection have also been developed. These organoid models enabled high throughput screening of FDA-approved drugs and identified entry inhibitors of SARS-CoV-2. Organoids also help reveal CNS targeting of ZIKV and the connection between infection and clinical manifestations of microencephaly. Using neurospheres and brain organoids, it has been shown that ZIKV targets human brain cells, reducing their viability and consequently abrogating neurogenesis during human brain development.^871^ In another study, forebrain organoids, which showed preferential, productive infection in neural progenitors, were used to model ZIKV exposure.^872^ Increased cell death and reduced proliferation resulted in decreased neuronal cell layer volume, which resembles microcephaly. Subsequently, Watanabe et al. described more susceptibility receptors for ZIKV and screened various compounds for ZIKV infection using organoid methods.^873^ Interestingly, the neuroinvasive capacity of SARS-CoV-2 was confirmed in brain organoids, characterized by clear infection of neuronal and neural stem cells, followed by neuronal cell death in both target cells and neighboring cells.^874^ These versatile platforms are essential for modeling human organ development and disease, and for compound testing including potential antiviral drugs against WHO high-priority disease. However, there are still many challenges facing the use of organoids. Since the human body is an integrated organism, all organs are regulated by network-like activities through signaling through hormones, cytokines, and other signaling molecules. However, in independent organoids, it is difficult to simulate active in vivo conditions although increasing cultural complexity has already been accomplished by adding stroma, interorgan communication, and the microbiome.^875^ In addition, high cost, variability and tissue heterogeneity further complicate the in vitro manipulation of organoids.

Concerted interdisciplinary efforts would be another direction for disease models. More recently, AI has been actively involved in screening antiviral drugs, diagnostics and synthetic biology.^876,877^ Additionally, AI has been used in the field of infection biology in infectious diseases. Viral pathogens trigger a complex host reactions, in which the viral load, host immunity, intervention methods, and other factors determin the progression of infection.^878^ In this regard, supervised machine learning models have been used to analyze structured and unstructured datasets of nucleic acids, proteins, carbohydrates, and cell phenotypes to identify key features and molecular networks involved in host pathogen interactions and immune responses.^879–881^ Both supervised and unsupervised machine learning models, including random forest classifiers and complex language models, have been applied to identify gene and protein interactions related to host cell changes, predict immunogenicity, evaluate pathogen eradication ability, and evaluate host cell adaptation. Interestingly, a conceptual bridge between natural language and viral evolution has been reported, which modeled viral escape with machine learning algorithms originally developed for human natural language. In this model, potential escape mutations of the SARS-CoV-2 S protein were identified using sequence data alone.^879^ Overall, machine learning has made significant contributions to the analysis of large and often complex datasets in infectious disease research.^876^ By integrating high-throughput datasets with detailed mechanistic studies, experiments, and infection models, the problems of low throughput and limited universality of AI guidance methods can be solved. For example, experiments that systematically obtain and analyze large-scale datasets in different infection backgrounds through comprehensive CRISPR screening, RNA sequencing, and mass spectrometry techniques will promote the development of AI models that go beyond data analysis tools and can propose generalizable hypotheses and reasoning. Parameterizing these efforts with biological sequences or chemical structures will provide adjustable methods for studying infections. In addition, machine learning has effectively processed microscopic datasets related to infection biology. Similarly, AI can improve and optimize the selection and design of experimental animal models by analyzing large amounts of data, such as predicting the response of disease models under specific genetic backgrounds or phenotypes, and guiding the construction of more accurate humanized models.

The use of AI in vaccine and drug design were more extensive. The core role of AI in vaccine design is to dissect immunogenic viral proteins by examining their complex structure, followed by determination of components that are most likely to trigger a robust and broad-spectrum immune response.^882,883^ Among them, immune negative selection is an important algorithm. It does not require prior knowledge and can effectively defend against unknown human invasion patterns. For drug design, AI has enabled the discovery and validation of molecular targets. The deep knowledge of coding can be designed and evaluated using machine learning algorithms, which can be fully applied to the traditional single target drug discovery project. For drug mining, AI has integrated independent professional knowledge in the fields of medicine, physics, or material science.^884^ Through deep learning optimization, rapid and pertinent organization and connection to this knowledge were accomplished. AI can automatically identify correlations and propose corresponding drug candidates and further screen molecular structures effective for some diseases, thus facilitating the development of new drugs. Compound screening refers to the method of selecting compounds with high activity for specific targets from a large group of compounds through standardized experimental methods, which has proven to be time consuming.^885^ Atomwise, an AI company in Silicon Valley, has developed the artificial intelligence molecular screening (AIMS) project, which aims to accelerate drug screening.^886^ The atomnet system was developed based on convolutional neural networks, which have learned a lot of chemical knowledge and research data. This system analyzes the structure-activity relationship of compounds, determines the basic modules in medicinal chemistry, and is used for new drug discovery and new drug risk assessment. At present, atomic systems have mastered much chemical knowledge and research data. In 2015, Atomnet simulated two promising compounds for Ebola virus treatment in only one week. In addition, the absorption, distribution, metabolism, excretion and toxicity (ADMET) property is the most important reference index for measuring the drug properties of compounds.^887^ Compound ADMET prediction is important in contemporary drug design and drug screening. The early ADMET properties of drugs mainly involved the use of human or humanized tissue functional proteins as drug targets. In vitro research combined with computer simulation was used to study the interaction between drugs and biophysical and biochemical barrier factors in vivo. To further improve the accuracy of ADMET property prediction, some enterprises have explored the effective extraction of structural features through deep neural network algorithms, which accelerated the early detection and screening process of drugs and greatly reduced the R&D investment and risk.^888^ Polymorphism is a phenomenon in which a substance can exist in two or more different crystal structures.^889^ For chemical drugs, almost all solid drugs have polymorphisms. Because changes in the crystal form can change many physical and chemical properties of solid chemical drugs, several drugs fail to be marketed due to crystal form problems. Therefore, crystal form prediction is of great significance in the pharmaceutical industry. With AI, the effective dynamic configuration of drug crystal forms can be used to predict all possible crystal forms of small molecule drugs. In addition, crystal form prediction technology greatly shortens the development cycle of crystals and more effectively selects the appropriate drug crystal form. Drug repositioning was deemed to be an effective strategy for improving the efficacy of existing drugs, finding new indications and using them to treat other diseases.^890^ Based on the powerful literature reading and cognitive reasoning ability of AI, the best matching order could be selected and ranked in a few minutes from large pools of drugs. Taken together, these novel technologies may complement or replace traditional animal models in the future, which could facilitate the development of more effective countermeasures against these infectious diseases.

## Conclusion remarks

Given the urgent circumstances of potential social and economic impacts caused by WHO high-priority pathogens, a timely response should be adopted in terms of pathogenesis, transmission mechanisms, and medical countermeasures. More importantly, “Disease X”, an infectious disease caused by unknown pathogens that could lead to a global pandemic, highlights the urgent need for preparations. In this process, the establishment of accurate animal models that reflect the authentic situation in humans is a prerequisite. Traditional approaches, together with novel technologies such as CRISPR and surrogate models, would provide and enrich the choices of animal models. However, many more issues need to be addressed in the future due to the scarcity and ethics of NHPs, the scarcity of BSL-3/4, the insusceptibility of common laboratory animals to WHO high-priority pathogens, and the discrepancy between humans and other species. Similarly, for their prevention and control, vaccines, antibodies, small molecular drugs, and other therapies have been developed based on existing and emerging technologies, and benefit from interdisciplinary cooperation. For example, organoid models offer humanized three-dimensional structures and functions for infection that are similar to those of organs in vivo. This would replace, reduce and in some cases, refine the use of traditional animal models in the study of WHO high-priority diseases. In addition, AI and machine learning can be used to build high-throughput databases for host-pathogen interactions, which can further optimize model selection, vaccine design, drug discovery, and antibody screening. Consequently, further technological advances, applied innovations and interdisciplinary cooperation are needed to gain better knowledge and consolidate the line of defence against WHO high-priority pathogens.
